# Metabolic and Morphotypic Trade-Offs within the Eco-Evolutionary Dynamics of Escherichia coli

**DOI:** 10.1128/spectrum.00678-22

**Published:** 2022-09-28

**Authors:** Nikola Zlatkov, Moa Elsa Cecilia Näsman, Bernt Eric Uhlin

**Affiliations:** a Department of Molecular Biology, Umeå Centre for Microbial Research (UCMR), Umeå University, Umeå, Sweden; Forschungszentrum Jülich GmbH

**Keywords:** *Escherichia coli*, ExPEC, NMEC, citrate utilization, filamentation, metabolomics, phase variation, proteomics

## Abstract

Escherichia coli arbitrarily encompasses facultative anaerobic, rod-shaped bacteria with defined respiratory and fermentative types of metabolism. The species diversification has been further advanced by atypical strains whose features deviate from the essential species-specific morphological and metabolic cutoff. The morphological cutoff is exemplified by bacterial filamentation. E. coli filamentation has been studied from two different perspectives: the first considers filamentation as a result of adaptive strategies and response to stress, while the second is based on findings from the cell division of E. coli’s conditional mutants. Another cutoff is represented by E. coli’s inability to use citrate as a sole carbon and energy source. In this study, we compared two atypical E. coli strains that belong to the same neuroinvasive ecovar but exhibit either of the two phenotypes that deviate from the species’ features. While E. coli RS218 exists in the form of filaments incapable of growth on citrate, strain IHE3034 is represented as normal-sized bacteria able to ferment citrate under oxic conditions in the presence of glucose; in this paper, we show that these two phenotypes result from a bona fide trade-off. With the help of comparative proteomics and metabolomics, we discovered the proteome required for the upkeep of these phenotypes. The metabolic profiles of both strains reveal that under aerobic conditions, RS218 undergoes oxidative metabolism, while IHE3034 undergoes anaerobic respiration. Finally, we show that the use of citrate and filament formation are both linked in a trade-off occurring via a c-di-GMP-dependent phase variation event.

**IMPORTANCE** Aerobic use of citrate and filamentous growth are arbitrary cutoffs for the Escherichia coli species. The strains that exhibit them as stable phenotypes are called atypical. In this study, we compare two atypical neuroinvasive E. coli strains, which alternatively display either of these phenotypes. We present the proteome and metabolome required for the maintenance of filamentous growth and show that anaerobic nitrate respiration is the main requirement for the use of citrate. The fact that the two phenotypes are differentially expressed by each strain prompted us to check if they are part of a trade-off. Indeed, these atypical characters are reversible and result from a c-di-GMP phase variation event. Thus, we revealed hidden links between stable morphological and metabolic phenotypes and provided information about alternative evolutionary pathways for the survival of E. coli strains in various host niches.

## INTRODUCTION

On contemporary time scales, ecology is bound up with evolution in the form of eco-evolutionary dynamics (1, 2). Such interactions may diversify the basic morphological and metabolic characters of individuals, leading to an increased heterogeneity of their population. This diversification may, on its own, bring forth an evolutionary and/or ecological change (3). A species whose representatives are subject to strong eco-evolutionary feedback is often recognizable by the diversity of its populations and the type of change these populations introduce in the environment (1).

Escherichia coli is one such group of versatile saprophytic biovars which have become accommodated in the habitats of a great many biotic (animal-associated) and abiotic (aquatic-related) environments. Even though predominantly commensal, some of the biovars have branched out into global intestinal and extraintestinal pathogens (4–9). As a result of the eco-evolutionary history of this species, E. coli’s populations have been classified into seven main phylogenetic groups, A, B (B1 and B2), C, D, E, F, and S (10, 11). The variety of E. coli’s populations is caused by the different ways novel fitness traits are mixed, matched, and integrated with the species’ life history traits. Resolved by natural selection, these complex events are aided by genetic (e.g., the mutator phenotype and gain and/or loss of genetic material) and physiological (e.g., phase variation) processes (10, 12, 13). Trade-offs make up a major feature of this integration of phenotypes; they take place when a fitness benefit in one trait has a negative impact on another. Within the eco-evolutionary framework of E. coli, trade-offs may result from nutrient deprivation; such is the case with the loss of the global stress regulator RpoS that triggers nutrient competence at the expense of the general stress resistance and with the metabolic trade-off between phosphate and glucose uptake (14, 15). Trade-offs can also stem from antibiotic stress—for example, the quinolone- and ciprofloxacin-resistant E. coli strains are less virulent than their sensitive counterparts—or be presented in the form of a reduced fitness as a result of social interactions due to phage resistance (16–20). Studies on the molecular mechanisms of life history trade-offs in E. coli are important for understanding the emergence of new strains, as well as the evolution of new traits at a higher species level.

E. coli also includes many “atypical” strains that display phenotypes that transcend the range of variation that is generally considered to define this species (6, 21). Such a pattern relies on shared morphological, physiological, and genetic characteristics that resonate with the past, present, and future experience of every species population, and under no circumstances will such a pattern be found in any other bacterial species. How such atypical phenotypes may influence environmental interaction, and contribute to E. coli’s evolution, is not yet understood. The first group of atypical phenotypes has a metabolic basis. Such phenotypes often result from the evolution of innovation, best exemplified by E. coli’s use of citrate. This trait is unlikely to evolve vertically on contemporary time scales due to the large number of generations required for adaptive evolution (22). Another group of atypical phenotypes is associated with filamentation, a well-known response of bacteria to stress, associated with the nonmutagenic events of the SOS stress response (23–29). Filamentation naturally occurs in the presence of DNA damage that the DNA repair machinery cannot cope with; it also presents itself in the form of a morphologic switch in the intracellular bacterial community’s (IBC) developmental program of strains that belong to uropathogenic E. coli (UPEC) (29–32). While SulA and DamX have been suggested as acting as effectors that trigger filamentation in IBCs, the eco-evolutionary drivers involved in the formation of this bacterial heterogeneity have yet to be described (29–32).

UPEC belongs to the group of extraintestinal pathogenic E. coli (ExPEC) (33). ExPEC is a vast ecological group within E. coli, the representatives of which may exist as “commensals” in the gastrointestinal tract (GIT), or, upon departing from the gut, they can invade almost every organ or system of the host, causing various rare or common (and potentially lethal) diseases in humans and other animals (8, 34–38).

Bracketed together with UPEC in the group of ExPEC are the pathogenic variants of neonatal meningitis-causing E. coli (NMEC). Besides NMEC’s ecological tendencies, which are intimately linked to their host and lifestyle transitions, the neuroinvasive E. coli, arguably the deadliest of all the neonatal pathogens, has long intrigued microbiologists and clinicians. The epidemiology, severity, relapse, long-term consequences, and outbreaks of the meningoencephalitis and early-onset sepsis disease its strains may cause have been the focus of research in this area for well over half a century (39–51). In addition to the physiology of their hosts, the fixed estimated recurrence of NMEC isolates in different geographical regions over time has also been attributed to their unusual metabolism (52). In short, the UPEC and NMEC representatives of ExPEC have close eco-phylogenetical links to each other, as they share some common virulence traits and survival strategies for host invasion, which suggests an ongoing evolution among them (53–55).

In this study, we explored NMEC strains IHE3034 and RS218, two neuroinvasive E. coli isolates that, together with some UPEC isolates, belong to the most metabolically diverse subphylogenetic group, B2 (56). Even though they belong to the same ecotype, these strains are part of two irreversibly divergent lineages, epitomizing the morphologic and metabolic borderlines of the E. coli species which may have evolved vertically. We found that RS218 exists in the form of filaments on solid media, while IHE3034 does not. Rather, as we previously reported (57), IHE3034 bacteria are able to ferment citrate under aerobic conditions when the cosubstrate glucose is provided. With the aid of comparative proteomics and metabolomics approaches, we investigated the molecular mechanisms governing their formation and maintenance. We also contrasted the filamentous morphotype exhibited by RS218 with the metabolic capability of IHE3034 for growth on citrate. In this paper, we show that these two atypical phenotypes may morph into each other in a phase variation manner, as in the case of IHE3034.

## RESULTS

### The cellular proteome that is required for the maintenance of filamentation.

**FIG 1:**
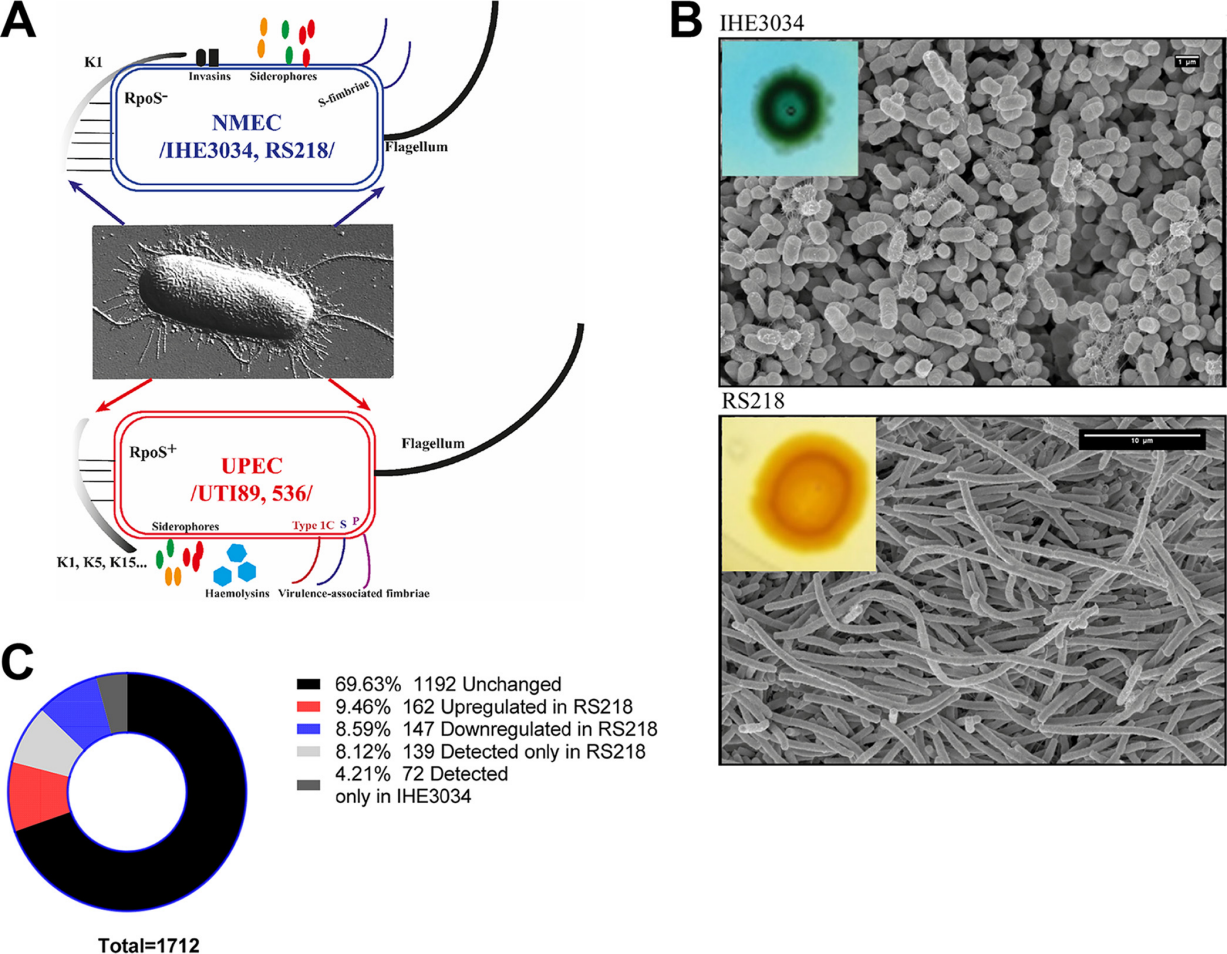

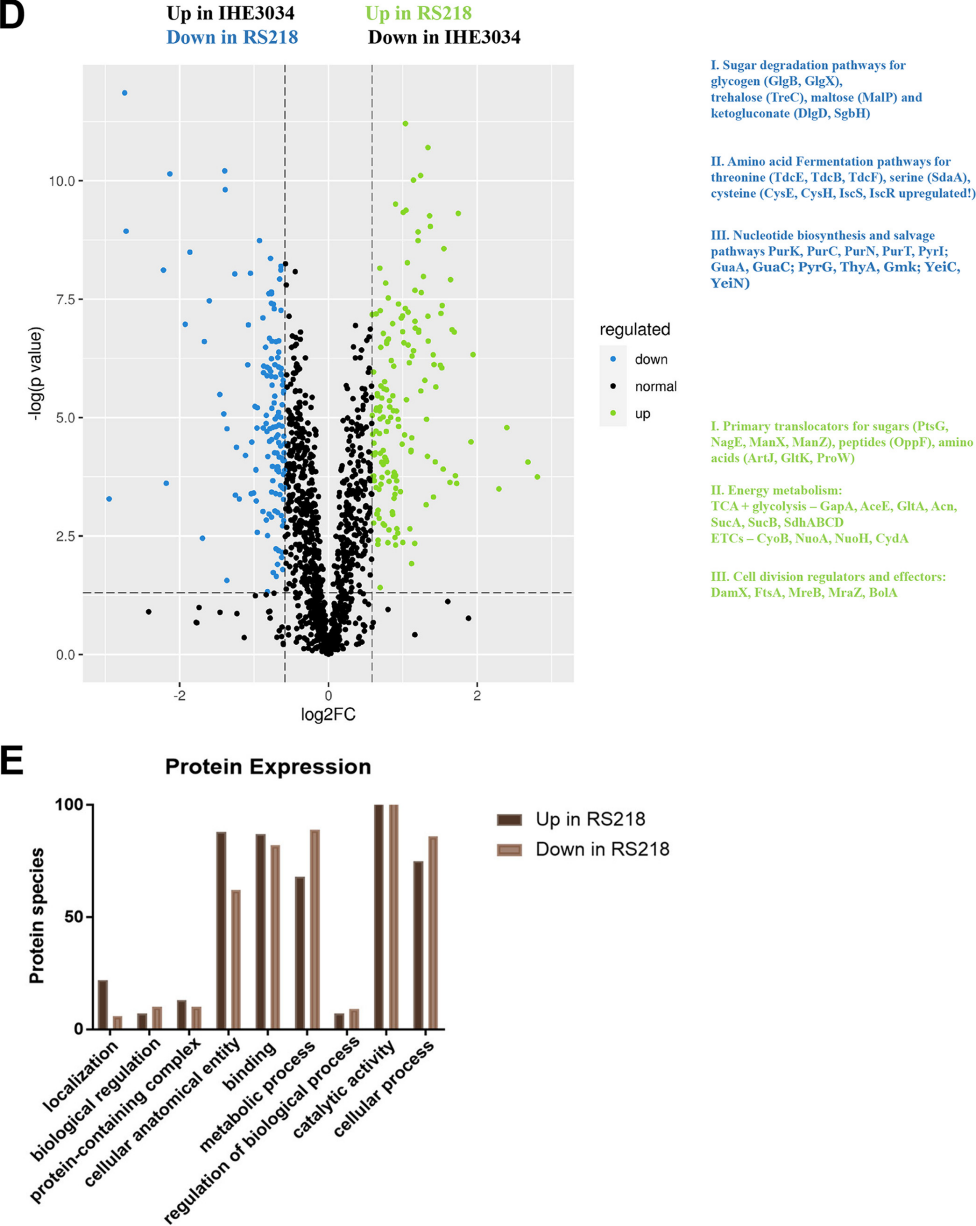
Comparison between NMEC strains RS218 and IHE3034. (A) Schematic representation of NMEC (RS218 and IHE3034) and UPEC (UTI89 and 536) in the group of ExPEC. While the UPEC strains have quite a diverse arsenal of virulence factors, common fitness factors of NMEC strains RS218 and IHE3034 encompass the K1 capsule, production of siderophores, S-fimbrial biogenesis, invasins, and inactive RpoS (the general stress response sigma subunit). Of note, RS218 is beta-hemolytic. (B) Macro- and micromorphological differentiation between strains IHE3034 and RS218. On solid media, the bacteria of IHE3034 exist in the form of normal-sized bacteria (scale bar, 1 μm), while the bacteria of RS218 exist as filaments (scale bar, 10 μm). When grown on modified Simmons's agar in the presence of glucose, the bacteria of IHE3034 are capable of citrate utilization (blue colony), while the ones of RS218 cannot use citrate (yellow colony). (C) General representation of the detected protein species and their distribution in RS218 and IHE3034. (D) Volcano plot depicting the upregulated (green) and downregulated (blue) proteins in RS218 compared to ones of IHE3034 and depicting the normally distributed proteins (in black) between these two strains. (E) Functional classification of the differentially expressed proteins detected in RS218.

Escherichia coli strains IHE3034 and RS218 are typical neonatal isolates that share similar, more specific virulence factors (compared to the UPEC) and the O18:K1:H7 serotype, as outlined in Fig. 1A. We recently discovered that the bacteria of IHE3034 are capable of aerobic citrate use in the presence of glucose, a capability not displayed by RS218; see Fig. 1B and reference 57. Besides this metabolic difference, we have also found that cells of the two strains are morphologically distinct in a way that is revealed when grown on solid media: the cells of IHE3034 are short (ca. 1.1 to 1.5 × 1 to 2 μm) rods, typical for the E. coli species, whereas the RS218 bacteria exist in the form of filamentous cells, longer than 10 μm (Fig. 1B). Since the two strains share a high percentage of chromosomal sequence identity (99.98%; see Fig. S1A in the supplemental material) and amino acid identity (99.52%; see Fig. S1B), we investigated whether these phenotypic observations reflect part of a metabolic-morphotypic trade-off that may drive the NMEC adaptation.

To begin, we undertook a comparative, label-free proteomics approach, which resulted in the detection of 1,712 proteins. Hierarchical clustering (Fig. S1C) showed a clear grouping of the protein species extracted from both strains. The level of 69.63% of the proteins we detected remained unchanged in both strains; 9.46% of the proteins were upregulated in RS218, and 8.59% were downregulated (Fig. 1C to E; Table S1). A total of 139 protein species were detected only in RS218 and 72 only in IHE3034 (Fig. 1C and Table 1). Figure 1D displays a volcano plot of the differentially expressed proteins, which are also summarized in Table S1. As shown in Fig. 1D and E, the majority of the differentially expressed proteins in RS218 belong to two main groups of physiological processes linked to metabolism and the maintenance of the cellular anatomy.

**TABLE 1:** Protein species detected only in one of the two strains

Protein species^*a*^	IHE3034	RS218
Virulence factors		
Yersiniabactin receptor	−	+
Fe^3+^ enterobactin outer membrane transporter	−	+
Colibactin biosynthesis acyltransferase	−	+
Enterotoxin TieB protein; plasmid-based gene	−	+
Type II toxin CcdB	−	+
Hypothetical protein	−	+
Complement resistance protein	−	+
Iron transporter	−	+
Cytolethal distending toxin type IV subunit A	+	−
Cytolethal distending toxin type IV subunit B	+	−
Cytolethal distending toxin type IV subunit C	+	−
Morphology		
MinD	+	−
MinE	+	−
MreC	−	+
Metabolism		
Putative sulfoquinovose aldose-1-epimerase	−	+
Succinate/quinone oxidoreductase	−	+
l-Fucose isomerase	−	+
Cytochrome *bd-I* ubiquinol oxidase subunit II	−	+
Xylose isomerase	−	+
Fructose-1,6-bisphosphatase	−	+
Formate dehydrogenase O subunit β	−	+
Fumarate reductase subunit C	−	+
Long-chain acyl-CoA thioesterase III	−	+
l-Glyceraldehyde 3-phosphate reductase	−	+
3-Isopropylmalate dehydratase small subunit	−	+
Putative transcriptional repressor for genes of catabolism of sulfoquinovose	+	−
l-serine deaminase III	+	−
1-Deoxy-d-xylulose 5-phosphate reducto-isomerase	+	−
Anaerobic dimethyl sulfoxide reductase chain B	+	−
NADH:quinone oxidoreductase subunit M	+	−
l-Serine deaminase II	+	−
α-Amylase	+	−
Periplasmic endochitinase	+	−
Hexitol phosphatase A	+	−
2-Oxoglutarate decarboxylase	+	−

a +, detected; −, not detected.

**FIG 2:**
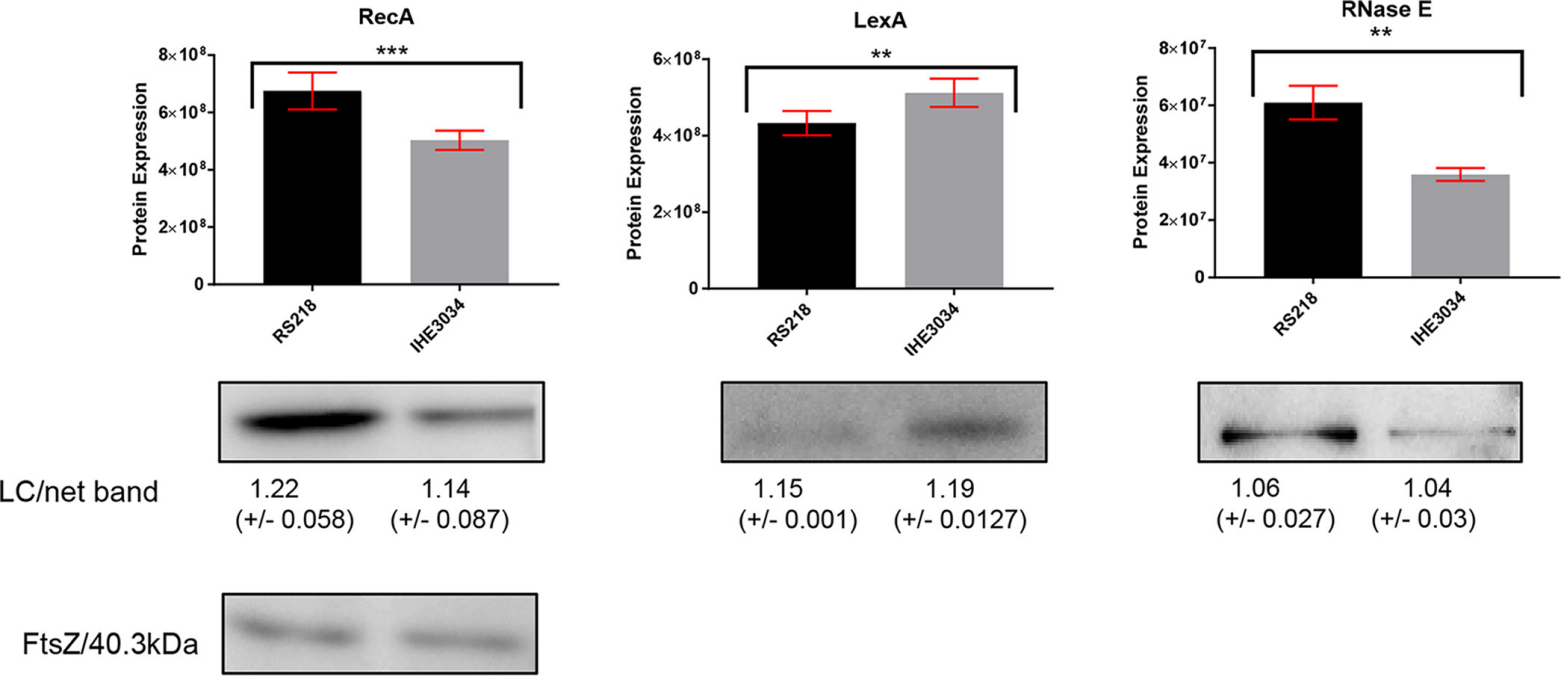
Validation of the protein expression of RecA, LexA, and RNase E. The protein levels obtained from the proteomics study were compared to the levels calculated from the intensity resulting from immunoblotting.

To delineate a possible molecular mechanism by which RS218 maintains the filamentous morphotype, we compared the expression levels of proteins known to be either regulators or direct participants in cell elongation. We investigated whether the filamentation could be initiated by the canonical activation of the SOS stress response via the cleavage of LexA through the action of RecA (23, 58). The proteomics data, as well as the immunoblotting experiments, demonstrated decreased levels of LexA and increased levels of RecA, suggesting a more active SOS response in RS218 than IHE3034 (Fig. 2). We also detected changes in the RNase E levels, suggesting a differential regulation of mRNA levels. The nonvalidated effectors of cell morphology and degradosome components are shown in Fig. S1D, and the list of proteins at levels below detection in one of the two strains is shown in Table S2.

Finally, we screened a collection of ExPEC pyelonephritis (Fig. S1E), cystitis (Fig. S1F), and meningitis (Fig. S1G) isolates for any links between the use of citrate and their morphology. As we previously showed (57), most of the pyelonephritis isolates we tested were citrate positive (all except IHE1041) and exhibited normal cellular morphology. On the other hand, the cystitis isolates included in our study tested negative for use of citrate but also exhibited normal cell size. Finally, while all our meningitis strains were able to use citrate, only IHE3074 displayed pleomorphism, which highlighted that RS218 has a rare, atypical morphotype.

### Trade-off between citrate utilization and filamentation in NMEC resulting from a phase variation event.

In addition to RS218’s inability to use citrate, we found that IHE3034 mutants deficient in the c-di-GMP phosphodiesterases (PDEs) SfaY and YcgG2 exhibited a stronger growth-on-citrate phenotype on modified Simmons’s plates (57). This finding led us to test whether the overproduction of SfaY, whose gene is part of a polycistronic operon, may participate in a trade-off between the use of citrate and cell elongation.

**FIG 3:**
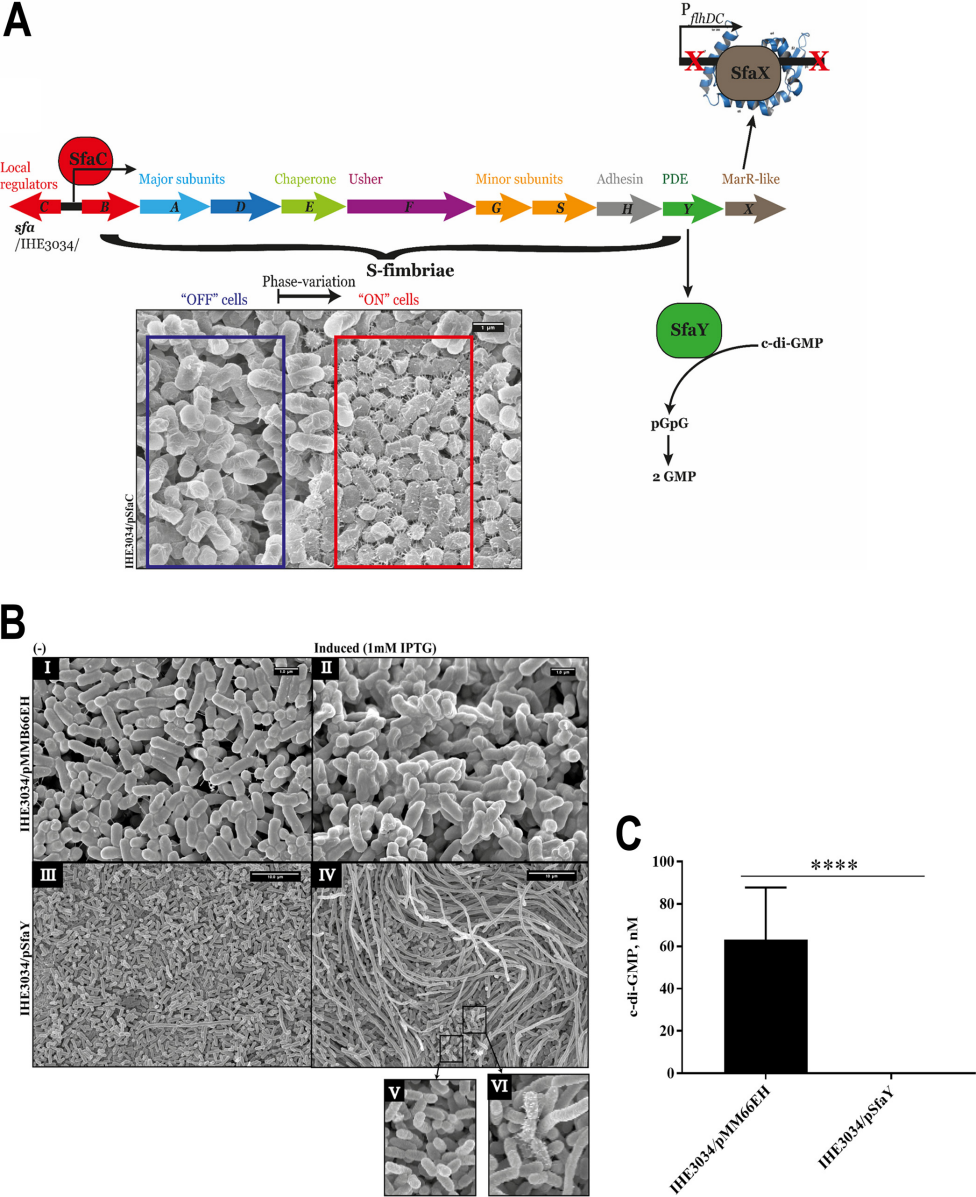
SfaC/SfaY-dependent phase variation events. (A) Schematic representation of the genetic determinants of the S-fimbriae. Phase variation is aided by the interaction of the local regulator SfaC (shown in red) with the global regulator Lrp (not shown) and, thus, the bacteria transition from “off” to “on” states (shown in the blue and the red framed areas, respectively, in the micrograph below the *sfa* operons). Additionally, the roles of the two regulatory genes *sfaY* and *sfaX* are highlighted. (B) Overproduction of SfaY in IH3034. Box I depicts wild-type bacteria in the absence of the inducer IPTG, and box II indicates its presence (scale bar, 1 μm). The wild-type bacteria carrying pSfaY in the absence of the inducer are shown in box III, and those in its presence are shown in box IV (scale bar, 10 μm). The presence of heterogeneous bacterial population of IHE3034/pSfaY is broken down to filamentous bacteria (box IV), normal-sized, nonfimbriated bacteria (box V), and fimbriated bacteria (box VI). (C) Effect of SfaY overproduction on c-di-GMP levels. Results from measurements of c-di-GMP extracted from bacteria of strains IHE3034/pMMB66EH and IHE3034/pSfaY.

The *sfaY* gene belongs to the *sfa* gene cluster of E. coli IHE3034, consisting of operons that code for the S-fimbrial biogenesis (Fig. 3A). The expression of this gene cluster is subject to a phase variation event, which is dependent on epigenetics, as well as on the activity of global (CRP, Lrp, and H-NS) and local (SfaC) regulators (Fig. 3A).

We decided to clone the *sfaY* gene in the low-copy-number plasmid pMMB66EH (empty vector [EV]) for the overexpression of SfaY in strain IHE3034 and to microscopically examine the effect on the bacterial cells. When SfaY was overproduced, the bacteria were depleted of c-di-GMP (Fig. 3C), and when incubated on lysogenic agar (LA) plates, a subpopulation of the cells gave rise to a filamentous morphotype similar to that observed in RS218 (Fig. 1B and Fig. 3B, box IV), whereas the rest of the population was overrepresented by small fimbriated and nonfimbriated bacteria (Fig. 3B, boxes V and VI).

Since only a minor fraction of the population of bacteria became filamentous, we tested whether the observed event would depend on phase variation, which controls the expression of the *sfa* gene cluster. We tested if the frequency of filamentation would be altered when the main promoter of the *sfa* gene cluster was locked in its “on” phase. For this purpose, we used the established means by overproducing the SfaC/PapI protein from a plasmid construct (59, 60).

**FIG 4:**
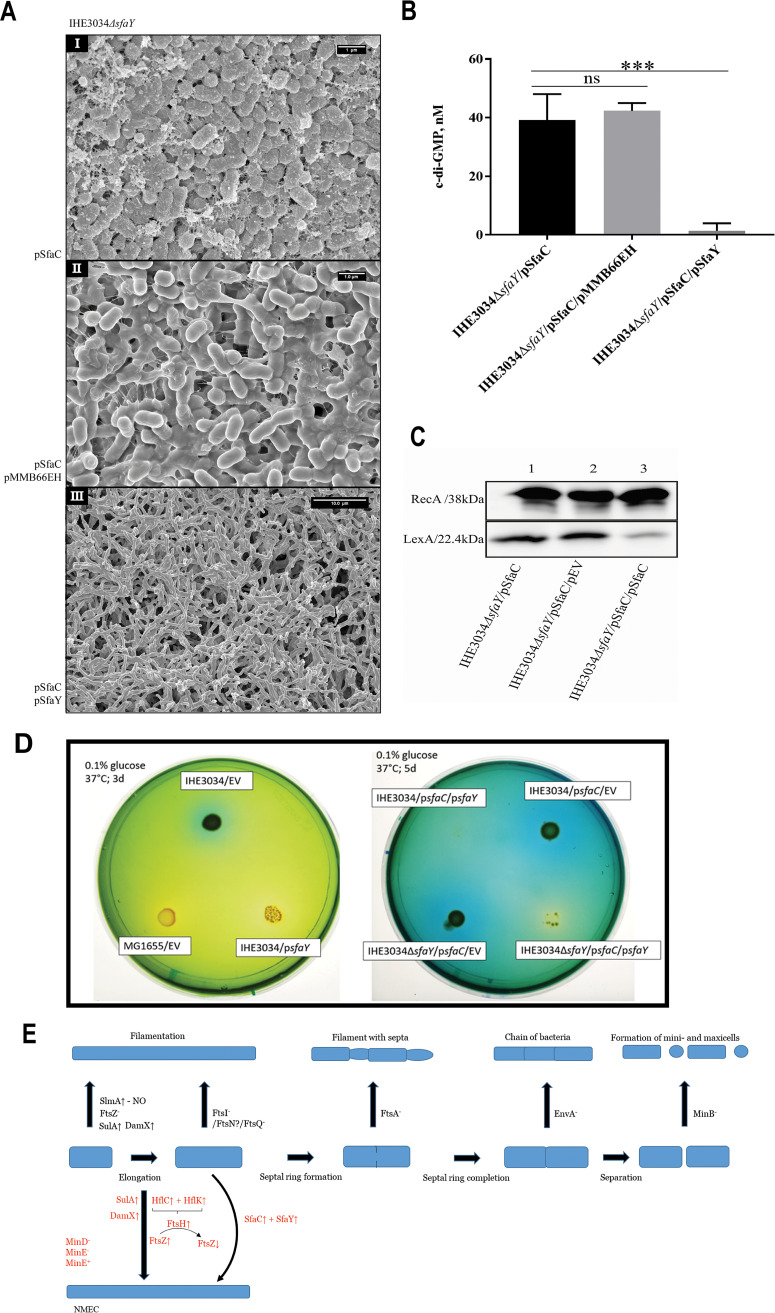
Metabolic and morphotypical outcomes of the phase variation of the *sfa* gene cluster. (A) SEM images of the IHE3034*ΔsfaY* mutant bacteria expressing pSfaC and therefore induced to be in their “on” state for transcription of the *sfa* gene cluster (box I). Box II shows IHE3034*ΔsfaY*/pSfaC/pMMB66EH bacteria, and box III shows IHE3034*ΔsfaY*/pSfaC/pSfaY (scale bar for box I and box II, 1 μm; scale bar for box III, 10 μm) (B) Measurements of c-di-GMP extracted from IHE3034*ΔsfaY* derivatives without or with SfaY and induced by SfaC to be in the “on” state. (C) Assessment of the RecA and LexA levels in IHE3034*ΔsfaY* “on” state, IHE3034*ΔsfaY* derivatives without or with SfaY induced by SfaC to be in their “on” state. Immunoblotting results obtained with antisera raised against RecA and LexA, respectively. (D) Conditional citrate utilization assay (Simmons’s solid media embedded with 0.1% glucose). (D, Left) Colonies exhibiting a blue halo are considered positive for citrate uptake, i.e., wild-type strain carrying the empty vector plasmid, IHE3034/EV. The wild-type E. coli K-12 strain MG1655 carrying the vector plasmid was used as citrate negative control and displayed a yellow halo. (D, Right) Derivatives of IHE3034 induced by SfaC to be in their “on” state. (E) Schematic summary of the present findings about filamentation of the NMEC bacteria and a comparison with the different types of the earlier described filamentous bacterial morphotypes (filaments, septated filaments, chains of bacteria, and the maxi-cells) resulting from genetic studies on bacterial cell division.

SfaC is the main positive local regulator of the *sfa* gene cluster and to keep the strains in an “on” phase (Fig. 3A), we overproduced SfaC in *trans* together with SfaY. The new strains were incubated on LA plates. The results revealed an increase in filamentation, reaching more than 50% of the cells in the observed population (Fig. 4A, box III). The c-di-GMP levels were also decreased in the SfaY-deficient “on” strain (Fig. 4B), and the extracellular matrix of the bacterial population was diminished (Fig. S2A). We also observed the same trend for filamentation exhibited by the wild-type IHE3034 “on” bacteria (Fig. S2B).

After learning that the induced filamentation of IHE3034 bacterial cells is dependent on the phase variation event and mediated by SfaY, we wanted to unravel the downstream part of this molecular mechanism. Since previous reports have shown that the SOS stress response is an important part of the UPEC pathogenesis and that filamentation can also result from DNA-damaging signals, one plausible hypothesis would be that the observed filamentation had been triggered via SOS stress response mimicry. To test this reasoning, we checked the levels of the main participants in the SOS response LexA and RecA in the cells of the induced “on” strains. The RecA levels remained the same in all strains, but the level of the repressor protein LexA was much reduced in the filamentous cells (see Fig. 4C and Fig. S2C for quantification). We also investigated whether the absence of the main repressor of the *sfa* gene cluster, H-NS, could result in bacterial filamentation. Figure S2D shows the “on” strains deficient in H-NS. Interestingly, the bacteria started elongating only upon induction of the SfaC, and even though they remained elongated when pSfaY was introduced, the levels of c-di-GMP remained the same (Fig. S2D, box I).

On the other hand, the presence of SfaC or SfaY in RS218 did not alter their morphotypes (Fig. S2E), nor did the absence of SfaY in the RS218 deletion mutant (Fig. S2F), which suggests a fixed morphological phenotype. When we introduced a deletion in the *sulA* gene of RS218, we observed a highly reduced number of elongated bacteria (Fig. S2G), which confirmed that the RS218 bacteria maintained an active SOS response.

Having succeeded in making the IHE3034 bacteria filamentous with the help of SfaY, to assess the possible connection between the morphological and metabolic properties as a trade-off, we tested the ability of the constructed strain to use citrate (Fig. 4D). While the IHE3034 bacteria carrying the empty vector (IHE3034/EV) yielded a positive result for citrate use, the bacteria that overexpressed SfaY (IHE3034/p*sfaY*) displayed retarded growth (Fig. 4D, left). The effect of overexpressed SfaY became more pronounced in the case of the IHE3034 “on” strains with the concomitant overexpression of SfaC (Fig. 4D, right). While the wild-type and SfaY-deficient “on” strains could use citrate, the overexpression of SfaY in their corresponding “on” strains resulted in a failure to use this substrate and in retarded growth of IHE3034*ΔsfaY*/pSfaC/pSfaY, whereas a synthetic lethal phenotype was evident in the case of IHE3034/pSfaC/pSfaY (Fig. 4D).

Next, we were interested in how conserved this switch would be among the various ExPEC strains. We created “on” strains expressing SfaY in UTI89 (a cystitis isolate) and in IHE3030 (a meningitis isolate); see Fig. S2H and I. In comparison to IHE3030, the presence of SfaC on its own triggered filamentous differentiation in UTI89 (Fig. S2H) but also deprived IHE3034 of citrate utilization (Fig. S2I).

Overall, our findings show that these NMEC strains may undergo a metabolic-morphotypic trade-off that provides the bacteria with certain adaptive traits such as filamentation. Figure 4E summarizes the aggregated data from the proteomics and microscopy experiments and puts the data in the framework of what researchers have experimentally proven over the decades about E. coli bacterial cell division. We should note, though, that while experimental studies using the commensal type of E. coli have involved conditionally lethal mutants, the naturally occurring filamentation of NMEC can be considered an adaptive and reversible trait exhibited preferentially only on a solid medium.

### Metabolic profiling reveals that RS218 bacteria tend to use oxidative energy metabolism, whereas IHE3034 bacteria exploit anaerobic respiration.

We next investigated RS218 filamentation and the physiological capacity for its upkeep; accordingly, we undertook a comparative, untargeted metabolomic approach to compare the metabolic status of RS218 to that of IHE3034.

To investigate the global variations in both the RS218 and IHE3034 metabolomes, we performed supervised regression modeling to analyze the hits acquired in electrospray ionization (ESI)-negative and ESI-positive modes. Figure S3 summarizes the primary data obtained from the ESI-negative mode (Fig. S3A to C) as well as from the ESI-positive mode (Fig. S3D to F). The base peak intensity chromatograms of a representative from each group are shown in Fig. S3A and D for the ESI-negative and ESI-positive modes, respectively.

**FIG 5:**
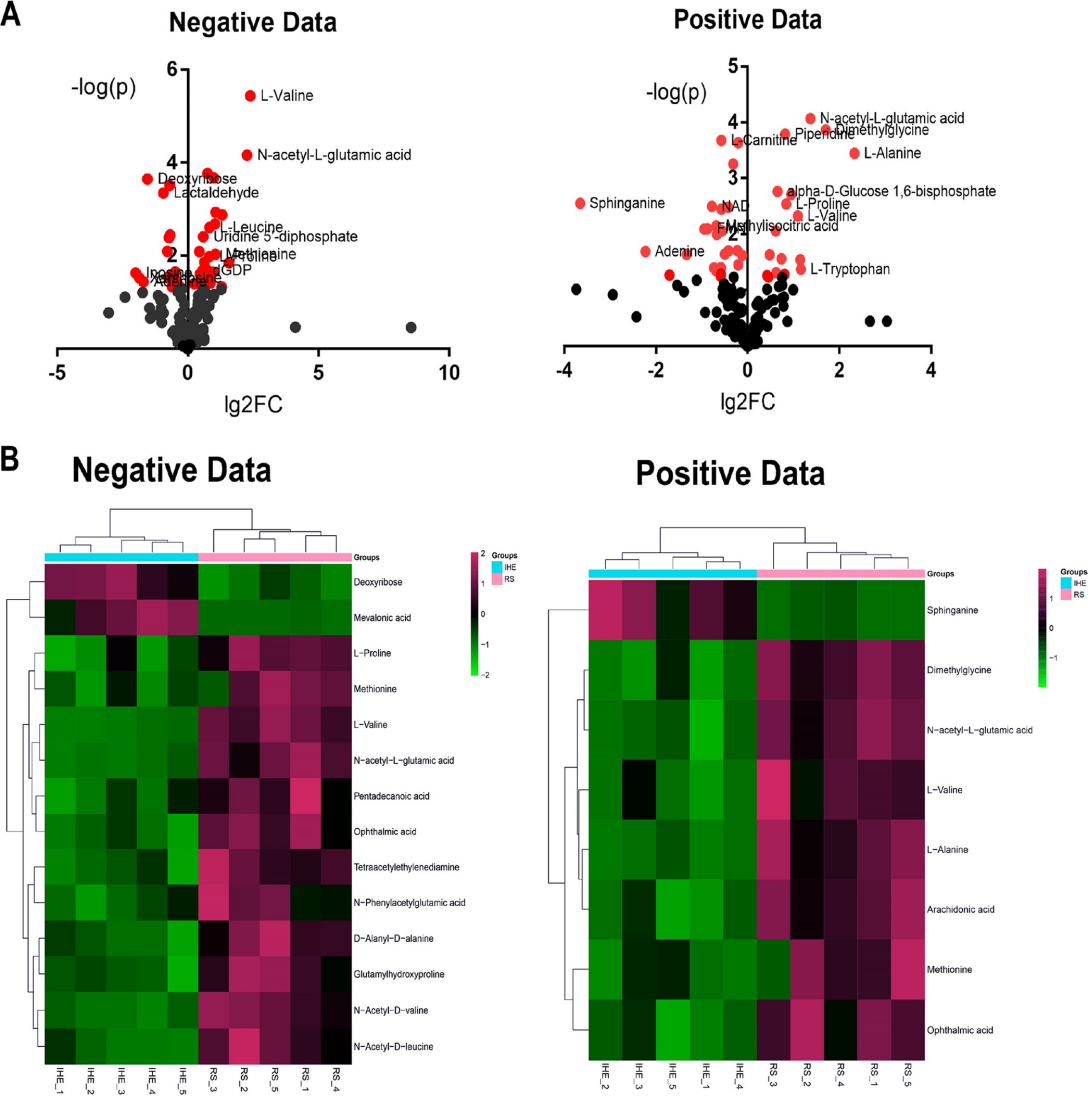
Comparative metabolomics of the NMEC strains RS218 and IHE3034. (A) Volcano plots of the perturbed metabolites of RS218 obtained from the ESI-negative mode, negative data, and ESI-positive mode, positive data, and normalized against the metabolites extracted from IHE3034. (B) Heatmaps of the most significantly perturbed metabolites. The 5 replicates of IH3034 (from IHE_1 to IHE_5) clustered separately from the group of RS218 (RS_1 to RS_5).

The clear grouping of the RS218 and IHE3034 samples in the partial least-squares discriminant analysis (PLS-DA) and orthogonal projections to latent structures discriminant analysis (OPLS-DA) score plots are shown in Fig. S3B and E for negative and positive data, respectively. The untargeted metabolomics identified and verified 154 metabolites in the ESI-positive mode and 168 metabolites in the ESI-negative mode (Table S3). Subsequently, the significantly altered metabolites between the two groups were filtered (variable importance in projection (VIP) > 1.5, fold change [FC] > 2 and < 0.5, *P* < 0.05) and plotted (Fig. S3C and F and Fig. 5A). The overall metabolic map of RS218 is displayed in Fig. S4.

**FIG 6:**
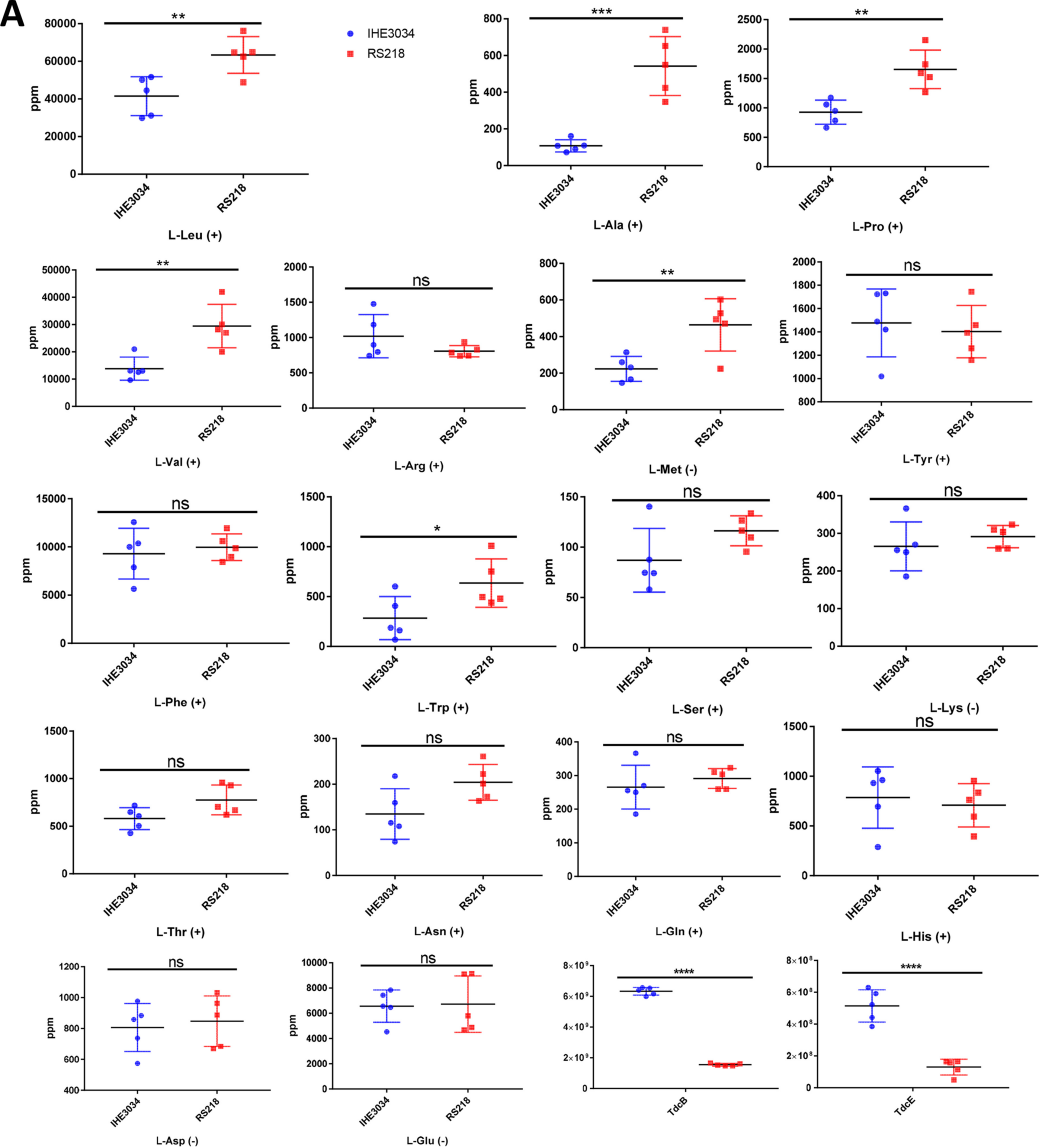

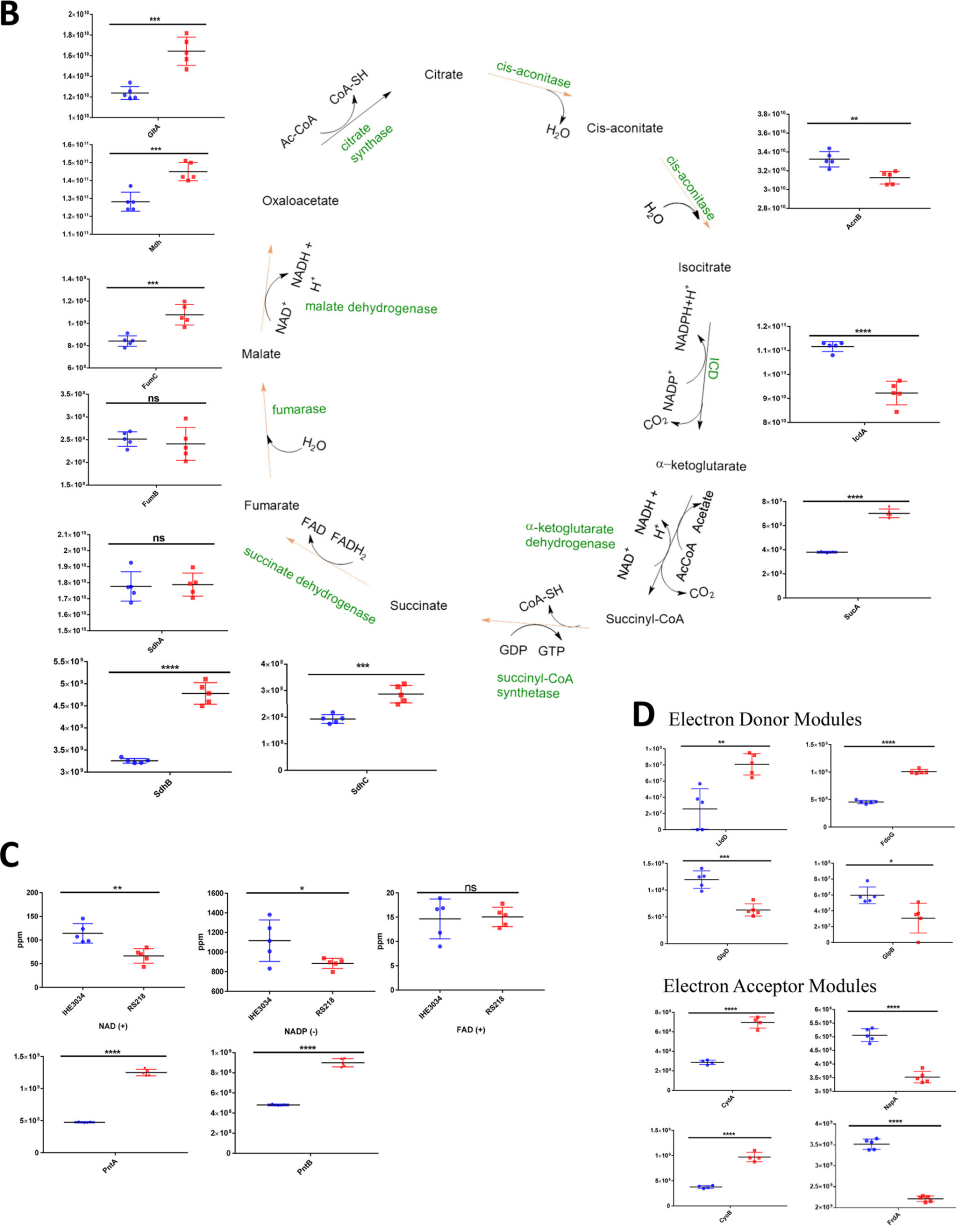
Relative abundance of the detected amino acids and nucleotides involved in redox reactions as well as of the detected protein species linked to the NMEC central metabolism. (A) Quantification of the detected amino acids and their distribution in RS218 and IHE3034 (the mode of detection is indicated as “+” or “−” after the name of the compound). Additionally, the levels of TdcB and TdcE are displayed in relative expression units. (B) Relative abundance of the TCA enzymes detected in this study (the levels of detected enzymes in samples from RS218 [in red] and the ones from IHE3034 [in blue]; the reversible reactions from the TCA cycle are marked with orange arrows; ICD, isocitrate dehydrogenase). (C) Quantitative analysis of NAD, NADP, and FAD detected in RS218 and IHE3034 (the mode of detection is indicated as “+” or “−” after the name of the compound). Additionally, the levels of PntA and PntB are displayed in relative expression units. (D) Relative abundance of the detected electron donor and acceptor modules in this study (proteins isolated from RS218 in red and the ones from IHE3034 in blue).

The first observed RS218 metabolic pattern, indicating differences from that of IHE3034, was the higher concentration of the nonpolar amino acids l-Leu, l-Val, l-Ala, l-Pro, and l-Met (Fig. 5A and B and Fig. 6A). This observation is consistent with the proteomics data, which show that the enzymes that metabolize l-Lys and l-Arg (DapB, DapD, and LysA); l-Cys (CysE, CysH, and IseS); l-Asp and l-Asn (IaaA and AsnA); and l-Trp, l-Tyr, and l-Leu (TrpB, TrpE, and TyrB) were downregulated in RS218 (Table S1). Additionally, those enzymes that participate in the fermentation of l-Thr and l-Ser (TdcB and SdaA) also exhibited lower levels of expression (Table S1). The higher levels of the ABC transporters for l-Arg (ArtJ), l-Glu/l-Asp (GltK, GltL, and GltJ), l-Gln (GlnP), and l-Gly/l-Pro/betaine (ProW) suggest that RS218 bacteria rely on the import of amino acids and peptides (OppF) from the host rather than their *de novo* synthesis, as may be the case for IHE3034 (Table S1).

We also detected further changes in the protein species of RS218, which participate in the transport of sugars (PtsG, NagE, ManX, and ManZ), whereas the IHE3034 upregulated proteins mainly participate in sugar degradation pathways for glycogen (GlgB and GlgX) and nucleotide biosynthesis and salvage pathways (PurK, PurC, PurN, PurT, and PyrI; GuaA and GuaC; PyrG, ThyA, and Gmk; and YeiC and YeiN); see Table S1. Furthermore, the most significantly increased metabolites, as illustrated in Fig. 5B, suggest that RS218 filaments maintain their turgor pressure via osmolytes (such as dimethylglycine), which is intimately linked to RS218 adaptation to oxidative stress, exemplified by the upregulation of YbgL, BtuE, OsmC, CueO, MsrP, and KatG (Table S1) and with the presence of ophthalmic acid (Fig. 5B).

The proteomics and metabolomics data also revealed that RS218 bacteria may undergo oxidative to nonoxidative metabolism; the latter is undergone by IHE3034. Evidence for the tendency of RS218 for oxidative metabolism is the upregulation of the membrane-bound transhydrogenase PntAB (Fig. 6C), which has been previously shown to be the central pathway, together with the pentose phosphate pathway, for the generation of NADPH under aerobic, glucose-rich conditions (61).

Indeed, the levels of GltA, Mdh, FumC, SdhB, and SdhC from the reductive part of the tricarboxylic acid (TCA) cycle were increased, while the levels of AcnB and IcdA from the oxidative TCA part were decreased, as were the levels of SucA (alpha-ketoglutarate dehydrogenase); see Fig. 6B. Integral to the TCA cycle are the modules for electron transport. The proteomics data demonstrated that, again, the RS218 bacteria tended to undergo oxidative over anaerobic metabolism. The elevated levels of LldD and FdoG (which use lactate and formate, respectively, as electron donors) and the levels of CydA and CyoB (terminal cytochrome oxidases, part of the oxygen-dependent acceptor module) are a case in point (Fig. 6D). In contrast to RS218, we detected, in the IHE3034 cells, a higher expression of GlpD and GlpB, which use glycerolphosphate as an electron donor, and of NapA and FrdA, which are part of the electron-acceptor module for nitrate and fumarate anaerobic respiration, respectively (Fig. 6D).

To verify whether anaerobic respiration in the case of IHE3034 metabolism takes place aerobically, we incubated the wild-type IHE3034 bacteria in Koser’s medium in the presence and absence of 0.1% glucose. We included 15 mM nitrate, 15 mM fumarate, or both. After overnight incubation, we measured the residual levels of citrate left in the medium. Figure S5A displays the concentration of the remaining citrate in the medium. While the presence of fumarate did not trigger any change in the citrate metabolism, the presence of nitrate did.

Finally, although such use is atypical, we aimed to explore how frequent the use of citrate is among different intestinal pathogenic E. coli strains. We screened representatives from the groups of enterohemorrhagic (EHEC), enteroaggregative (EAggEC), enterotoxigenic (ETEC), enteroinvasive (EIEC), enteropathogenic (EPEC), and adherent-invasive (AIEC) E. coli (Fig. S5B to G). Interestingly, the EHEC and AIEC strains involved in this study tested positive for citrate on Simmons’s medium with glucose (Fig. S5B and G), and most of the representatives of EAggEC, ETEC, and EPEC were also prone to use citrate (Fig. S5C, D, and F). Only the representatives of EIEC were not able to take in citrate (Fig. S5E).

## DISCUSSION

### Trade-offs within the eco-evolutionary dynamics of Escherichia coli.

Besides phenotypic variations due to natural selection, trade-offs (also known as antagonistic pleiotropy), too, play an active role in the eco-evolutionary cycles of E. coli. Our findings show that they can constrain the evolution among certain phenotypes; promote phenotypic plasticity, such as phase variation; or trigger evolutionary transitions when one component of the environmentally induced trade-offs becomes a life history trait that has been permanently fixed.

Our studies also show that these negative covariances, in addition, can be dynamic. Ensuing from phase variation, in the case of IHE3034, such metabolic and morphotypic transitions are part of a reversible ecological trade-off. We have demonstrated that proteins acquired from horizontally acquired regulatory genes, i.e., SfaC and SfaY, can trigger filamentation with the loss of citrate utilization, which is aided by the SOS response and the c-di-GMP signaling system of this strain. The trade-offs’ dynamics are further enhanced by the findings that the negative covariances can be permanently fixed as a phylogenetic trait. As illustrated by the RS218 filamentous morphotype, the constitutively active SOS response has triggered morphological, physiological, and regulatory reorganization of these bacteria. Such kinds of temporal-to-permanent transitions allow different components of each trade-off to be independently (from its negative phenotypic counterpart) integrated, maximized, and evolved as a phylogenetic trait. Eventually, these events may bring about strains with novel life history traits that are atypical for the species.

### Maintenance of filamentation.

This study illuminates how two NMEC strains exhibit atypical stable phenotypes, and it provides insights into their regulation. In the case of RS218, the filamentation is upkept by the rearrangement of cytoskeletal proteins and the presence of oxidative metabolism. For IHE3034, its anaerobic nitrate respiration can enable aerobic growth on citrate. The c-di-GMP-mediated phase variation, shown in this study to be part of the IHE3034 *sfa* regulon, can serve as a switch between filamentation and citrate use.

Based on the proteomics data presented above, we conclude that NMEC filamentation is shaped via the integrated activities of the SOS stress response. A plausible explanation may be that due to the active SOS response, the levels of DamX and SulA are elevated, which leads to the inhibition of the divisome formation, an effect that is further enhanced by the increased levels of the FtsZ protease FtsH and its main regulators, HflK and HflC (for a summary, see Fig. 4E). Also, this phenotype could be reversely reconstituted in IHE3034 with the aid of SfaY and SfaC.

Furthermore, the fact that we could not detect MinE and MinD protein species (see Fig. S1D in the supplemental material, Table 1, and Table S2) suggests the rod cell shape-determining protein MreC, which counteracts the Z-ring formation of the cell poles (62), may also be involved in the maintenance of RS218 filamentation via the sequestering of the FtsZ monomers.

After considering the RS218 filamentation with metabolic capacity for its maintenance and the present proteomics and metabolomics data, we found that RS218 bacteria tend to undergo oxidative metabolism as exemplified by the increased levels of enzymes from the TCA cycle, as well as the components of the oxygen-dependent electron transport chains. Whether the detected ophthalmic acid, structurally similar to glutathione, is an indicator of adaptation to oxidative stress or is a biomarker of changes in the RS218 sulfur metabolism is an open question that will be a topic of interest for further analyses since little is known about the role of ophthalmic acid in bacteria.

### Trade-off between metabolism and morphology mediated via SfaC/SfaY phase variation.

We previously reported that, due to the loss of RpoS (the general stress response sigma factor), the NMEC strain IHE3034 is capable of aerobic growth on citrate in the presence of glucose. This capability results from a competition between RpoD and RpoS sigma factors that is resolved in favor of RpoD (57). In anaerobic environments and in the presence of a cosubstrate (such as glucose), E. coli can feed on citrate (63, 64). Under these special conditions, the CitT transporter is expressed, and the cosubstrate catabolism provides the reduced equivalents, which supply the malate dehydrogenase and fumarate reductase, the enzymes required for the fermentation of citrate to succinate (63, 64). The combined data of our experiments suggest that, unlike RS218, the bacteria of IHE3034 exhibit anaerobic respiration even under aerobic conditions (see Fig. 6D for comparison of electron donor and acceptor modules, and for aerobic citrate uptake in the presence of nitrate and glucose, see Fig. S5A).

The overproduction of the c-di-GMP phosphodiesterase SfaY impinged on IHE3034’s capacity for citrate fermentation. We also discovered a new role of the phase variation event which led to filamentation in a subpopulation of NMEC E. coli; this filamentation took place in a c-di-GMP-regulated manner triggered by SfaY. The *sfaY* gene is part of the *sfa* gene cluster, and its expression is maintained by the regulators involved in *sfa* gene expression. The regulation of the *sfa* gene cluster is achieved, upon proper methylation of the proximal promoter GATC site, by the local regulator SfaC, which binds to the proximal site together with Lrp (a global regulator), which, in turn, stabilizes the “on” phase of the gene cluster (65–68). Strains overexpressing SfaC/PapI have been reported to be the most common cause of NMEC outbreaks (48). We constructed phase “on” strains that overproduced SfaC, and we tested whether SfaY overproduction might change their morphology. When we coexpressed SfaC together with SfaY, the bacteria of the whole population became filamentous. The link between filamentation and the SOS response is achieved by the presence of both SfaC and SfaY proteins, as demonstrated via immunoblotting. While the overexpression of SfaC on its own did not prevent citrate fermentation, its coexpression with SfaY resulted in a synthetic lethal phenotype displayed by the wild type. In short, this work also uncovered an ecological trade-off; when the IHE3034 cells transitioned from normal-sized bacteria to filaments, they lost their ability to grow on citrate.

### Filamentation and ExPEC.

The filamentation described above may be likened to the phenomenon observed with UPEC, first reported from studies of the cystitis E. coli isolate UTI89 in a murine infection model (29, 69) and later detected in patients with urinary tract infections (70, 71). During its intracellular lifestyle in the bladder surface cells, many bacteria from the IBCs exist as long filamentous bacteria together with small motile and nonmotile bacterial variants (29). This differentiation is essential for the infectious potential of the strain because the filamentous bacteria disrupt the host cells, propelling the small, contagious bacteria and thus spreading the infection. Because of their size, they also protect the small bacteria from being phagocytosed by macrophages (29, 31).

Several metabolic requirements were found for the UPEC filamentation, active gluconeogenesis, the TCA cycle, and amino acid metabolism (72). Interestingly, these metabolic requirements are identical to those for RS218 filamentation, as suggested by our proteomics and metabolomics studies. Even though the filamentation of UTI89 was recently discovered to depend on the type of urine and its pH (73), UTI89 filaments have been observed only intracellularly, and researchers have been unable to reconstitute the IBCs *in vitro* to date. So far, the factors that trigger this morphological plasticity have been identified as the activities of DamX and SulA (29, 32, 69). Although the groups have discovered the role of these effectors, the selective mechanism that decides on a subpopulation level which bacteria need to become elongated has remained elusive. We suggest that the mechanisms revealed to be behind the NMEC filamentation, as shown in this study for IHE3034 (phase variation, combined with c-di-GMP signaling, leading to activation of the SOS stress response), represent a feasible explanation.

### The role of atypical phenotypes that transcend accepted ranges of variation for a species in species delineation.

Biologists currently view Escherichia coli as a complex species with semidefinite boundaries and a clonal population structure, the lineages of which exhibit miscellaneous levels of divergence ensuing from mosaic-sympatric and sympatric stages of delineation (74, 75). In this work, we have demonstrated cryptic metabolic/physiological diversity exhibited by the pathogenic variants of E. coli. Although our phenotypic studies showed a decoupling between strains’ phylogeny and metabolism, the grouping of the pathogenic variants by the aerobic use of citrate in the presence of glucose revealed certain tendencies. While the group of EIEC tested negative for the use of citrate, all the EHEC and AIEC strains we examined were positive. We also found a correlation between the use of citrate and the microniche from which the ExPEC strains we tested were isolated. All but one of the pyelonephritis isolates elicited a positive result for citrate uptake, whereas all the cystitis isolates tested negative. Altogether, these findings further underline the extent to which genotypic cohesiveness may be decoupled from metabolism.

Based on Cohan’s theory, bacterial species are monophyletic, ecologically distinct populations joined by forces of genetic cohesion, such as recombination and natural selection (76). We showed that NMEC strain IHE3034 can undergo phase variation for the maintenance of dynamic phenotypes. Thus, not only is phase variation an additional cohesive force but it can also trigger metabolic and morphotypic transitions that transcend the accepted ranges of variation for the E. coli species. When this cohesive force is selected against (i.e., when a subpopulation remains fixed in one phase), the lack of phase variation may lead to a permanent morphotypic divergence, as in the case with RS218, the bacteria of which exist as long filaments maintained by fixed SOS response; in other words, the populations of RS218 can be represented as an ecotype of E. coli under speciation.

Whether the atypical phenotypes, such as filamentation and the use of citrate, can transcend the bacteria outside the accepted range of the E. coli species has yet to be shown. Yet the metabolic and morphotypic characters described in this study are versatile phenotypes, and if not controlled by phase variation, they can set off microevolutionary events.

## MATERIALS AND METHODS

### Bacterial strains and growth conditions.

All bacterial strains (listed in Table S1 in the supplemental material) were cultured aerobically at 37°C unless stated otherwise in the particular section. Cells were cultured in lysogenic broth (LB) or on agar plates (LA) with the appropriate amount of the following antibiotic(s) or inducer when needed: carbenicillin, 100 μg/mL; tetracycline, 15 μg/mL; and 1 mM IPTG (isopropyl-β-d-thiogalactopyranoside; Sigma). To test for the ability to use citrate, we used modified Simmons’s medium, as previously described in reference 57. One representative of three independent experiments is shown. Incubation in Koser’s citrate broth (Condalab) was done overnight, and where necessary, 0.1% glucose, 15 mM nitrate, and 15 mM fumarate were included. The citrate levels were measured with a citrate assay kit (Sigma-Aldrich), following the manufacturer’s instructions.

### Genetic experiments.

All plasmids and oligonucleotide primers used in this work are listed in Tables 2 and 3. The sequences of E. coli IHE3034 (GenBank accession no. NC_017628) and E. coli RS218 (GenBank accession no. CP007149) chromosomes were used for performing all genetic experiments in this study. The whole-chromosome alignment was done by BLAST (https://www.ncbi.nlm.nih.gov/). The amino acid sequence identity was calculated by the AAI calculator (http://enve-omics.ce.gatech.edu/aai/) developed by Rodriguez-R and Konstantinidis (77).

**TABLE 2:** Bacterial strains and plasmids used in this work

Escherichia coli isolates and plasmids	Description/relevant characteristic	Source or reference
IPEC isolates		
EHEC		
4732/98	O145:H^−^	Iwanaga^*a*^
4602/97	O26	Iwanaga
EAggEC		
O111-11	O111:H21	Iwanaga
O126-60	O126:H16	Iwanaga
O127a-3	O127a:H21	Iwanaga
O151-2	O151:H2	Iwanaga
O44-20	O44:H16	Iwanaga
ETEC		
O115-23	O115:H5	Iwanaga
O126-4	O126:H12	Iwanaga
O128-15	O128:H12	Iwanaga
O128-37	O128:H12	Iwanaga
O136-1	O1236:H5	Iwanaga
EIEC		
O136-5	O136:H51	Iwanaga
O146-2	O146:H14	Iwanaga
O146-9	O146:H14	Iwanaga
O164-16	O164	Iwanaga
O28ac-1	O28ac:H7	Iwanaga
EPEC		
O111-2	O111:H27	Iwanaga
O111-5	O111:H6	Iwanaga
O111-23	O111:H9	Iwanaga
O119-4	O119:H4	Iwanaga
O124-12	O124:H21	Iwanaga
AIEC		
LF82	Crohn’s disease isolate	84
LF9	Crohn’s disease isolate	84
LF15	Crohn’s disease isolate	84
LF16	Crohn’s disease isolate	84
LF31	Crohn’s disease isolate	84
ExPEC isolates		
UPEC		
IHE1041	Pyelonephritis isolate, O1:K1:H7	85
IHE1049	Pyelonephritis isolate, O1:K1:H7	85
IHE1190	Pyelonephritis isolate, O18:K5:H7	85
IHE1268	Pyelonephritis isolate, O18:K5:H7	85
IHE1402	Pyelonephritis isolate O6:K2:H1	85
IHE1431	Pyelonephritis isolate, O6:K2:H1	85
CCUG 55212	Cystitis isolate, O17:K52:H18	CCUG^*b*^
CCUG 41427	Cystitis isolate, O4:K3:H5	CCUG
CCUG 24T	Cystitis isolate, O1:K1:H7	CCUG
UTI89	Cystitis isolate, O18:K1:H7	86
NZ111	UTI89/pHMG94	This work
NZ112	UTI89/pHMG94, pMMB66EH	This work
NZ113	UTI89/pHMG94, pAES22	This work
NMEC		
IHE3034	O18:K1:H7	85
NZ73	IHE3034/pMMB66EH	This work
AES153	IHE3034*ΔsfaY*	This work
NZ76	AES153/pMMB66EH	This work
NZ77	AES153/pAES22	This work
NZ105	AES153/pHMG94	This work
NZ106	AES153/pHMG94, pMMB66EH	This work
NZ107	AES153/pHMG94, pAES22	This work
AES22	IHE3034*Δhns*	This work
NZ108	IHE3034*Δhns/*pHMG94	This work
NZ109	IHE3034*Δhns/*pHMG94, pMMB66EH	This work
NZ110	IHE3034*Δhns/*pHMG94, pAES22	This work
IHE3035	O18ac:K1:H7	85
IHE3039	O18ac:K1H7	87
IHE3040	O18ac:K1:H7	85
IHE3041	O18acK^−^:H^−^	85
NZ128	IHE3040/pHMG94	This study
NZ129	IHE3040/pHMG94, pMMB66EH	This study
NZ130	IHE3040/pHMG94, pAES22	This study
IHE3047	O18ac:K1:H7	85
IHE3070 (RK295)	R:K1:H7	87
IHE3074 (RK299)	R:K1:H7	87
IHE3079 (RK304)	O18ac:K1:H7	87
IHE3080	O18ac:K1:H7	85
RS218	O18:K1:H7	88
NZ134	RS218/pHMG94	This study
NZ135	RS218/pHMG94, pMMB66EH	This study
NZ136	RS218/pHMG94, pAES22	This study
NZ146	RS218*ΔsfaY*	This study
NZ153	RS218Δ*sulA773::aph*	This study
Plasmids		
pMMB66EH	Cb^r^, cloning vector, p*_tac_* promoter	89
pBR322	Cb^r^, Tc^r^, *rep* ori (pMB1), cloning vector	90
pKD3	Cb^r^, Cm^r^ FRT-cm-FRT	91
pKD4	Cb^r^, Km^r^ FRT-kan-FRT	91
pSIM6	Cb^r^, ori pSC101, temp sensitive, carries the α-red recombinase genes, induced at 42°C	92
pKM208	Cb^r^, ori pSC101, temp sensitive, carries the α-red recombinase genes, IPTG inducible	93
pKO3	Cm^r^, ori M13, *sacB, repA*^ts^, suicide vector	79
pHMG94 (pSfaC)^*c*^	pBR322, *papI* clone	59
pAES22 (pSfaY)	pMMB66EH, *sfaY* gene from the *sfa* gene cluster	94
pCP20	*repA101*, λ repressor, FLP	95

a From the laboratory collection of Masaaki Iwanaga, Department of Bacteriology, University of Ryukyus, Okinawa, Japan.

b From Culture Collection, University of Göteborg (CCUG), Department of Clinical Bacteriology, Göteborg, Sweden.

c PapI and SfaC are virtually identical and functionally exchangeable (53).

**TABLE 3:** Oligonucleotide primers used in this study

Oligonucleotide primer	Sequence (5′→3′)
*hns*mutup	CCTGGCTATTGCACAACTGAATTTAAGGCTCTATTATTAGTGTAGGCTGGAGCTGCTTC
*hns*mutdw	GTCTTAAACCGGACAATAAAAAATCCCGCCGATGGCGGGCATATGAATATCCTCCTTAG
*hns*upFw	TAACTATTCACAATCTTTAACCTGTTGCGCATGTA
*hns*dwRv	GCGGTAATAAATTAGGTTACATGCAGGC
EAL6up	GCGGATCCGGCGGTAATGATATTAC
EALdelendup	CTCTATATTGAGACTCTGCAGCAGGGATAATCCTTTTTCACAGAC
SfaII-3	CGGTCGACGGATCAGCATCACTAGG
EALdelstartdo	CTGCTGCAGAGTCTCAATATAGAGTGACATTACTCCTCCGG
Fw*sfaH*dw	GTGACTTTTAGCTACAACTAGAATGCAG
dd*sfaX*Rv	ATGCGGCCGCTGATAATCAATATCATTTAGCAAAAGAAAAAGCAA
*sulA*pKDFw	TGTACATCCATACAGTAACTCACAGGGGCTGGATTGATTATGTACGTGTAGGCTGGAGCTGCTTC
*sulA*pKD4Rv	AAGTTCCAGGATTAATCCTAAATTTACTTAATGATACAAATTAGACATATGAATATCCTCCTTAG
up*sulA*Fw	AAAATAGAGTTGATCTTTGTCGTCACTGGA
dw*sulA*Rv	AAATTAGTCACGACTGAAAGCATTGGC

The molecular genetic experiments were performed essentially as described by Sambrook et al. (78). The *sfaY* gene in the genome of RS218 was targeted by a PCR-generated deletion fragment cloned into the suicide pKO3 plasmid as previously described for the construction of the E. coli IHE3034*ΔsfaY* mutant strain (57). The construct was electroporated into the RS218 cells, and after obtaining heterogeneities, the following steps of mutagenesis were done as described by Link et al. (79).

The deletion of *hns* in the genome of IHE3034 was achieved with the help of pKM208, which expresses the lambda red recombinase upon IPTG induction. IHE3034/pKM208 bacteria were induced with 1 mM IPTG when they reached a growth optical density (OD) of 0.01 at 30°C and then grown until mid-exponential phase. After reaching the log phase, the cells were shifted to 42°C and incubated for 15 min, followed by 10 min incubation on ice. The *Δhns* deletion fragment was created via PCR with *hns*mutup and *hns*mutdw primers and the pKD3 plasmid as a template. One microgram of the deletion fragment was used for electroporation of electrocompetent cells, which were subsequently recovered after overnight incubation in LB aerobically at 37°C. The mutant cells were selected on LA with 25 μg/mL chloramphenicol (Cm^25^), and the genetic region of the *hns* deletion was verified via sequencing using hnsupFw and hnsdwRv primers. The antibiotic resistance cassette was removed with the help of the Flp recombinase expressed from the pCP20 plasmid.

The deletion of *sulA* in the genome of RS218 was achieved with the help of pSIM6, which expresses the lambda red recombinase upon induction at 42°C. IHE3034/pSIM6 bacteria were shifted from 30°C to 42°C when they reached a growth OD of 0.5 and then grown for another 30 min. The *Δhns* deletion fragment was created via PCR with *sulA*pKD4Fw and *sulA*pKD4Rv primers and the pKD4 plasmid as a template. One microgram of the deletion fragment was electroporated into the electrocompetent cells, and the cells were recovered overnight in LB aerobically at 37°C. The mutant cells were selected on LA with kanamycin at 50 μg/mL (Km^50^), and the genetic region of the *sulA* deletion was verified via sequencing using up*sulA*Fw and dw*sulA*Rv primers.

### Bacterial transformation.

The transformation of bacteria with plasmid DNA by electroporation was performed essentially as described by Fiedler et al. (80). In short, after the dilution of bacteria from overnight cultures in LB, the cells were grown to the exponential phase of growth; 50-mL samples of each bacterial strain were then harvested by centrifugation and washed with 25 mL of ice-cold, 10% aqueous glycerol. After three washing steps, 300 μL of the cell suspension was electroporated (2,500 V, 200 Ω, 25 μF) upon the addition of 10 ng of plasmid DNA (or 5 ng of each when bacteria were cotransformed with two different plasmids). Then, the cells were recovered for 1 h in LB aerobically at 37°C and plated on LA supplemented with the proper amount of antibiotic(s).

### Label-free proteomics analyses of RS218 and IHE3034 with nano-LC-MS/MS.

Protein extracts from five biological replicates of each strain were prepared as follows: cells from a colony were suspended in phosphate-buffered saline (PBS) and adjusted to 10^9^ bacterial cells in 1 mL; the cells were harvested by centrifugation and then washed three times with PBS buffer before resuspension in 1 mL of extraction buffer (150 mM NaCl, 0.5% sodium deoxycholate, 50 mM Tris-HCl, pH 8, and 5 μM E64 protease inhibitor). The cell suspensions were then subjected to freeze-thawing, with 3 cycles of deep freezing in liquid nitrogen, and each cycle was followed by a rapid thawing at 42°C. After the lysis, cell debris was pelleted by centrifugation (30 min, 15,000 × *g*, 4°C), and 1 mL of the supernatant was recovered for analysis.

A total of 200 μg of proteins from each sample was precipitated with the help of methanol and chloroform and then dissolved in 2 M urea aqueous solution, followed by denaturation with 10 mM dithiothreitol (DTT) at 56°C for 1 h and alkylation with 50 mM iodoacetamide. Finally, the proteins were digested by trypsin. One μg of protein from each sample was used for identification and quantification by nano-liquid chromatography-tandem mass spectrometry (nano-LC-MS/MS). The nano-LC separation was performed with an UltiMate 3000 nano-ultrahigh-performance liquid chromatography (UHPLC) system (Thermo Scientific). The mobile phase was composed of buffer A (0.1% formic acid in water) and buffer B (0.1% formic acid in acetonitrile), with a total flow rate as follows: 250 nL/min and an LC linear gradient from 2% to 8% buffer B for 3 min, from 8% to 20% buffer B for 60 min, from 20% to 40% buffer B for 33 min, and then from 40% to 90% buffer B for 4 min.

The full scan was performed between 300 and 1,650 *m/z* at a resolution of 60,000 at 200 *m/z*; the automatic gain control target for the full scan was set to 3E-6. The MS/MS scan was operated in top 20 mode using the following settings: resolution of 15,000 at 200 *m/z*; automatic gain control target, 1E-5; maximum injection time, 19 ms; normalized collision energy at 28%; isolation window of 1.4 Th; charge state exclusion unassigned, 1, and > 6; and dynamic exclusion, 30 s.

### Untargeted metabolomics analyses of RS218 and IHE3034 with UPLC-MS.

Metabolites from five biological replicates of each strain were extracted as follows. Cells from a colony were suspended in PBS and adjusted to 10^9^ bacterial cells in 1 mL; they were then harvested by centrifugation and washed three times with PBS. Then, the cells were resuspended in 200 μL of dichloromethane/methanol/water (1:3:1 ratio) at 4°C, vigorously mixed, and rocked for 1 h at 4°C. Finally, the suspensions were centrifuged (30 min,15,000 × *g*, 4°C), and then the supernatants were placed in fresh tubes and vacuum dried. Samples were thawed and dissolved with 400 μL of 80% methanol. All samples were next extracted at 4°C with ultrasound for 30 min and kept at −20°C for 1 h. Next, samples were vortexed for 30 s, kept at 4°C for 30 min, and centrifuged at 12,000 rpm and 4°C for 15 min. Finally, 200 μL of supernatant and 5 μL of dl-*o*-chlorophenylalanine (1 mg/mL) were transferred to a vial for LC-MS analysis.

Quality control (QC) samples were then used to evaluate the methodology. The same amount of extract was obtained from each sample and mixed as QC samples. The QC samples were prepared using the same sample preparation procedure. Separation was performed by the UltiMate 3000 LC machine combined with Q Exactive MS (Thermo) and screened with ESI-MS. The LC system consisted of an Acquity UPLC HSS T3 (100 by 2.1 mm by 1.8 μm) with Ultimate 3000 LC. The mobile phase consisted of solvent A (0.05% formic acid water) and solvent B (acetonitrile), with a gradient elution (0 to 1 min, 95% A; 1 to 12 min, 95% to 5% A; 12 to 13.5 min, 5% A; 13.5 to 13.6 min, 5% to 95% A; and 13.6 to 16 min, 95% A). The flow rate of the mobile phase was 0.3 mL min^−1^. The column temperature was maintained at 40°C, and the sample manager temperature was set at 4°C. The raw data were acquired and aligned using Compound Discover (version 3.0; Thermo) based on the *m/z* value and the retention time of the ion signals. When necessary, further confirmation was achieved through comparisons with authentic standards, including retention times and MS/MS fragmentation patterns.

### Western immunoblotting.

Procedures for gel electrophoresis and immunoblotting were performed as described in reference 57. Protein extracts of bacterial colonies grown on LA with or without antibiotics and inducer were used. To detect the levels of LexA, a polyclonal rabbit antibody was used against the LexA DNA binding region (Abcam; catalog no. ab174384). To detect RecA levels, a polyclonal rabbit antibody against full RecA (Abcam; catalog no. ab63797) was used. The RNase E antiserum used in this study was raised in rabbit (81). A representative replica of three independent repetitions of the Western blotting is shown. Quantification was done with the inverted pixel density of the targeted band from three independent experiments and is presented as a loading control/band-of-interest ratio.

### c-di-GMP measurements.

The total amount of cyclic nucleotides was extracted from about 1.6× 10^10^ bacteria grown aerobically on LA at 37°C overnight supplemented with the proper antibiotic(s) and inducer, according to the method described in reference 82. The measurements were done in triplicate and are presented in molar concentrations.

### Scanning electron microscopy.

The scanning electron microscopy (SEM) experiments were performed at the Umeå Core Facility for Electron Microscopy (UCEM). When necessary, the bacterial colonies were fixed with 2.5% glutaraldehyde (GA) in 0.1 M phosphate (PB), left overnight at 4°C, and then washed with 0.1 M PB buffer. Sedimentation was performed for 1 h on poly-l-lysine-coated glass coverslips (for liquid suspension), and then the samples were dehydrated in a series of graded ethanol (70 to 100%). Sample drying was done with a Leica EM CPD300 and the coating (platinum, 5 nm) with sputter coater (Quorum Technologies Q 150T ES). Samples were imaged via field-emission scanning electron microscopy (FESEM; Carl Zeiss; Merlin), with an accelerating voltage of 4 kV, probe current of 120 pA, and in-lens or in-chamber secondary electron detection.

### Statistical analyses.

**(i) Label-free proteomics.** Using MaxQuant (version 1.6.2.6) 10 raw MS files were analyzed and searched against the Escherichia coli K1 database based on the species of the samples. The parameters were set as follows: the protein modifications were carbamidomethylation (C) (fixed) and oxidation (M) (variable), the enzyme specificity was set to trypsin, the precursor ion mass tolerance was set to 10 ppm, and MS/MS tolerance was set to 0.5 Da. Only peptides identified with high confidence were chosen for downstream protein identification analysis. A quantitative ratio over 1.5 was considered upregulation, while a quantitative ratio less than 1:1.5 was considered downregulation.

**(ii) Untargeted metabolomics.** Ions from both ESI-negative or ESI-positive modes were merged and imported into the SIMCA-P program (version 14.1) for multivariate analysis. Principal-component analysis (PCA) was first used as an unsupervised method for data visualization and outlier identification. Supervised regression modeling was then performed on the data set by use of partial least-squares discriminant analysis (PLS-DA) or orthogonal projections to latent structures discriminant analysis (OPLS-DA) to identify the potential metabolites. Next, to select the variable metabolites, we used their variable importance in projection (VIP) scores to estimate the importance of each variable in the projection used in the discriminant analysis models. Metabolites with VIP scores of >1.5, FC of >2.0 and <0.5, and *P* value of <0.05 were defined as significant compounds.

Further statistical analysis of the experiments was performed with GraphPad Prism 7.04. Multiple comparison of samples was done with one-way analysis of variance (ANOVA), followed by Bonferroni adjustment, and a *t* test was performed for two-sample comparison. The significance is determined by a *P* value < 0.05, as follows; ns, *P* > 0.05; *, *P* ≤ 0.05; **, *P* ≤ 0.01; ***, *P* ≤ 0.001; and ****, *P* ≤ 0.0001; each bar represents mean ± standard error of the mean (SEM).

### Data availability.

The mass spectrometry proteomics data have been deposited to the ProteomeXchange Consortium via the PRIDE (83) partner repository with the data set identifier PXD036163.
