# Supplementary material for: Metabolic and Morphotypic Trade-Offs within the Eco-Evolutionary Dynamics of Escherichia coli
Source: Microbiol Spectr. 2022 Sep 28;10(5):e00678-22. doi: 10.1128/spectrum.00678-22 (PMC9602443; doi:10.1128/spectrum.00678-22)
Supplement: Supplemental file 1 — Supplemental material. Download spectrum.00678-22-s0001.pdf, PDF file, 6.8 MB [file spectrum.00678-22-s0001.pdf]

# **Metabolic and Morphotypic Trade-offs within the Eco-Evolutionary Dynamics of *Escherichia coli***

Nikola Zlatkov, Moa Elsa Cecilia Näsman, and Bernt Eric Uhlin

Department of Molecular Biology,
Umeå Centre for Microbial Research (UCMR),
Umeå University, SE-90187 Umeå, Sweden

**Table S1. List of significantly differentially expressed protein species in the strains[1]**

| Protein | Functional category | RS218 | IHE3034 |
|---|---|---|---|
| | 1. Nutritional and metabolic characteristics. | | |
| **Crp** | **cAMP receptor protein** | **Down** | **Up** |
| **CsrA** | **Carbon storage regulator A** | **Down** | **Up** |
| **SfsA** | **Sugar fermentation stimulation protein A** | **Up** | **Down** |
| | 1.1. Sugar degradation pathways. | | |
| | 1.1.1. Glycogen, trehalose and maltose degradation. | | |
| **GlgB** | **1,4-α-glucan branching enzyme** | **Down** | **Up** |
| **GlgX** | **Glycogen debranching enzyme** | **Down** | **Up** |
| **MalP** | **α-1,4-glucan phosphorylase** | **Down** | **Up** |
| **TreC** | **Trehalose-6-phosphate hydrolase** | **Down** | **Up** |
| **Glk** | **Glucokinase** | **Down** | **Up** |
| **TreF** | **Cytoplasmic trehalase** | **Up** | **Down** |
| **GlgP** | **α-1,4 glucan phosphorylase** | **Up** | **Down** |
| | 1.1.2. Fucose/Arabinose degradation. | | |
| **FucR** | **FucR-L-1-phosphate DNA-binding activator** | **Down** | **Up** |
| **FucK** | **L-Fuculokinase** | **Down** | **Up** |
| | 1.1.3. N-acetyl-D-glucosamine degradation. | | |
| **NagK** | **N-acetyl-D-glucosamine kinase** | **Down** | **Up** |
| **GlmM** | **Phosphoglucosamine mutase** | **Up** | **Down** |

1.1.4. D-galacturonate catabolism.

**UxaB Altronate oxidoreductase Up Down**

1.2. Feeding of pentose-phosphate and/or Entner-Doudoroff pathways.

**TktB Transketolase 2 Up Down**

**FbaB Fructose-bisphosphate aldolase class 1 Up Down**

1.2.1. Ascorbate degradation.

**DlgD (YaiK) 2,3-Diketo-L-gulonate reductase Down Up**

**SgbH 3-Keto-L-gulonate-6-phosphate decarboxylase Down Up**

1.2.2. Ketogluconate degradation.

**YiaE (GhrB) Glyoxylate/hydroxypyruvate reductase B Down Up**

**DkgA Methylglyoxal reductase Up Down**

1.2.3. Xylose degradation.

**XylB Xylulokinase Down Up**

1.2.4. Gluconeogenesis.

**MtfA Mlc titration factor A Down Up**

1.3. Fermentation

**TdcE 2-Ketobutyrate formate/pyruvate lyase Down Up**

**TdcB Threonine dehydratase Down Up**

**TdcF (YjgF) Enamine/imine deaminase Down Up**

**SdaA (TdcG) L-serine deaminase I Down Up**

**YahK NADPH-dependent aldehyde reductase Down Up**

| | | | |
|---|---|---|---|
| **Ppc** | **Phosphoenolpyruvate carboxylase** | **Up** | **Down** |
| **Pta** | **Phosphate acetyltransferase** | **Up** | **Down** |
| **LldD** | **L-lactate dehydrogenase** | **Up** | **Down** |
| **AldA** | **NAD-dependent aldehyde dehydrogenase** | **Up** | **Down** |
| **AldB** | **NAD-dependent aldehyde dehydrogenase** | **Up** | **Down** |
| **MaeB** | **$NADP^+$- dependent malic enzyme** | **Up** | **Down** |

1.4. Amino acid biosynthesis and degradation.

1.4.1. L-lysine and L-arginine biosynthesis and degradation

| | | | |
|---|---|---|---|
| **DapB** | **4-hydroxy-tetrahydrodipicolinate reductase** | **Down** | **Up** |
| **DapD** | **Tetrahydrodipicolinate succinylase** | **Down** | **Up** |
| **LysA** | **Diaminopimelate decarboxylase** | **Down** | **Up** |
| **GabD** | **Succinate-semialdehyde $NADP^+$ dehydrogenase** | **Up** | **Down** |
| **ArgI** | **Ornithine carbamoyltransferase** | **Up** | **Down** |

1.4.2. Sulphur metabolism, assimilatory sulfate reduction and L-cysteine biosynthesis

| | | | |
|---|---|---|---|
| **CysE** | **Serine acetyltrasferase** | **Down** | **Up** |
| **CysH** | **Phosphoadenosine phosphosulfate reductase** | **Down** | **Up** |
| **IscS** | **Cysteine desulfurase** | **Down** | **Up** |
| **IscR** | **HTH-type transcriptional repressor of *iscRSUA*** | **Up** | **Down** |

1.4.3. L-Aspartate biosynthesis, and L-asparagine biosynthesis and degradation.

| | | | |
|---|---|---|---|
| **IaaA** | **Isoaspartyl dipeptidase proenzyme** | **Down** | **Up** |
| **AsnA** | **Aspartate-ammonia ligase** | **Down** | **Up** |

1.4.4. L-tyrosine, L-phenylalanine, L-tryptophan biosynthesis and degradation, and L-leucine biosynthesis.

| | | | |
|---|---|---|---|
| **TrpB** | **Tryptophan synthase β chain** | **Down** | **Up** |
| **TrpE** | **Anthranilate synthase component 1** | **Down** | **Up** |
| **TyrB** | **Tyrosine aminotransferase** | **Down** | **Up** |
| **AroC** | **Chorismate synthase** | **Up** | **Down** |

1.4.5. L-glutamate and L-glutamine biosynthesis.

| | | | |
|---|---|---|---|
| **AlaA** | **Glutamate-pyruvate aminotransferase** | **Up** | **Down** |
| **GlnA** | **Glutamine synthetase** | **Up** | **Down** |

1.4.6. L-threonine biosynthesis.

| | | | |
|---|---|---|---|
| **ThrA** | **Aspartokinase/homoserine dehydrogenase 1** | **Up** | **Down** |

1.4.7. L-proline degradation.

| | | | |
|---|---|---|---|
| **PutA** | **Fused transcriptional repressor /Pro dehydrogenase** | **Up** | **Down** |

1.5. Biosynthesis of cofactors and prosthetic groups.

1.5.1. Biosynthesis of pantothenate.

| | | | |
|---|---|---|---|
| **PanD** | **Aspartate 1-decarboxylase** | **Down** | **Up** |
| **CoaA** | **Pantothenate kinase** | **Down** | **Up** |

1.5.2. Biosynthesis of folate.

| | | | |
|---|---|---|---|
| **FolD** | **Methylenetetrahydrofolate dehydrogenase** | **Down** | **Up** |
| **FolE** | **GTP cyclohydrolase 1** | **Up** | **Down** |

1.5.3. Lipoate biosynthesis

| | | | |
|---|---|---|---|
| **LipA** | **Lipoyl synthase** | **Down** | **Up** |
| | 1.5.4. Tetrapyrrole biosynthesis | | |
| **HemL** | **Glutamate-1-semialdehyde 2,1-aminomutase** | **Down** | **Up** |
| | 1.5.5. Pyridoxal 5'-phosphate biosynthesis. | | |
| **PdxH** | **Pyridoxine/Pyridoxamine 5'-phosphate oxidase** | **Down** | **Up** |
| **Epd** | **D-erythrose-4-phosphate dehydrogenase** | **Down** | **Up** |
| | 1.5.6. Molybdenum cofactor biosynthesis. | | |
| **MobA** | **Molybdenum cofactor guanylyltransferase** | **Up** | **Down** |
| | 1.5.6. Flavin biosynthesis. | | |
| **YbjI** | **5-amino-6-(5-phospho-D-ribityl-amino) uracil phosphatase** | **Up** | **Down** |
| | 1.5.7. Quinol biosynthesis. | | |
| **IspB** | **All-trans-octaprenyl-diphosphate synthase** | **Down** | **Up** |
| **IspF** | **2-C-Me-D-erythritol 2,4-cyclodiphosphate synthase** | **Down** | **Up** |
| **Dxr** | **1-deoxy-D-xylulose 5-phosphate reductoisomerase** | **Down** | **Up** |
| **UbiD** | **3-octaprenyl-4-hydroxybenzoate carboxy-ligase** | **Up** | **Down** |
| | 1.6. Nucleoside and nucleotide biosynthesis and degradation. | | |
| | 1.6.1. (Deoxy)Ribonucleotide biosynthesis and degradation. | | |
| **PurK** | **AIR[2] carboxylase** | **Down** | **Up** |
| **PurC** | **Phosphoribosylaminoimidazole-succinocarboxamide** | **Down** | **Up** |

| | | | |
|---|---|---|---|
| | synthase | | |
| PurN | GAR[3] transformylase 1 | Down | Up |
| PurT | GAR transformylase 2 | Down | Up |
| PyrI | Aspartate carbamoyltransferase regulatory chain | Down | Up |
| GuaA | GMP synthase | Down | Up |
| GuaC | GMP reductase | Down | Up |
| PyrG | CTP synthase | Down | Up |
| ThyA | Thymidylate synthase | Down | Up |
| Gmk | Guanylate kinase | Down | Up |
| YeiC (PsuK) | Pseudouridine kinase | Down | Up |
| YeiN (PsuG) | Pseudouridine-5-phosphate glycosidase | Down | Up |
| RutR | DNA-binding transcriptional repressor of *rut* and *gcl* | Down | Up |
| Dcd | Deoxycytidine triphosphate deaminase | Down | Up |
| NrdR (YbaD) | Transcriptional repressor of *nrd* | Down | Up |
| GuaB | Inosine-5-phosphate dehydrogenase | Up | Down |
| Amn | AMP nucleosidase | Up | Down |
| PpnN (YgdH) | Nucleotide 5’-phosphate nucleosidase | Up | Down |

1.6.2. Purine and pyrimidine nucleotide salvage pathways.

| | | | |
|---|---|---|---|
| Hpt | Hypoxanthine phosphoribosyltransferase | Down | Up |
| RihC | Non-specific ribonucleoside hydrolase | Down | Up |

1.7. Lipid metabolism

1.7.1. Glycerophosphodiester degradation

| | | | |
|---|---|---|---|
| **GlpQ** | **Periplasmic glycerophosphodiester phosphodiesterase** | **Down** | **Up** |

1.7.2. Fatty acid biosynthesis and degradation.

| | | | |
|---|---|---|---|
| **TesB** | **Acyl-CoA thioesterase II** | **Down** | **Up** |
| **FadB** | **Fatty acid oxidation complex subunit alpha** | **Up** | **Down** |
| **YqeF** | **Putative acetyl-CoA acetyltransferase** | **Up** | **Down** |
| **AccA** | **Acetyl-CoA carboxylase carboxyl transferase subunit α** | **Up** | **Down** |
| **AccD** | **Acetyl-CoA carboxylase carboxyl transferase subunit β** | **Up** | **Down** |

1.7.3. Phospholipid metabolism.

| | | | |
|---|---|---|---|
| **Psd** | **Phosphatidylserine decarboxylase proenzyme** | **Up** | **Down** |
| **PssA** | **CDP-diacylglycerol-serine O-phosphatidyltransferase** | **Up** | **Down** |

1.8. Transport proteins.

1.8.1. Channel proteins.

1.8.2. Electrochemical potential-driven transporters.

| | | | |
|---|---|---|---|
| **YiaO** | **2,3-diketo-L-gulonate:$Na^+$ symporter (periplasmic binding)** | **Down** | **Up** |
| **TonB*** | **Prophage-encoded TonB-like protein** | **Down** | **Up** |
| **PutP** | **$Na^+$/Proline symporter** | **Up** | **Down** |
| **AcrB** | **Multidrug efflux pump RND permease** | **Up** | **Down** |
| **CstA** | **Pyruvate/Oligopeptide:proton symporter** | **Up** | **Down** |
| **TcyP** | **Cystine/Sulfocystein:cation symporter** | **Up** | **Down** |

1.8.3. Primary active transporters.

1.8.4. Phosphotransfer-driven group translocators.

| | | | |
|---|---|---|---|
| **UlaC** | **PTS enzyme IIA$^{\text{L-Ascorbate}}$ component** | **Up** | **Down** |
| **PtsG** | **PTS enzyme IIBC$^{\text{Glucose}}$ component** | **Up** | **Down** |
| **NagE** | **PTS enzyme EIICBA$^{\text{N-Acetyl-D-glucosamine}}$ component** | **Up** | **Down** |
| **ManX** | **PTS enzyme IIAB$^{\text{Mannose}}$ component** | **Up** | **Down** |
| **ManZ** | **PTS enzyme IID$^{\text{Mannose}}$ component** | **Up** | **Down** |
| **MtlA** | **PTS enzyme IICBA$^{\text{Mannitol}}$ component** | **Up** | **Down** |

1.8.5. The ABC superfamily (the ATP-Binding Cassette superfamily).

| | | | |
|---|---|---|---|
| **DppA** | **Dipeptide ABC transporter periplasmic binding protein** | **Down** | **Up** |
| **YejA** | **Oligopeptide ABC transporter periplasmic binding protein** | **Down** | **Up** |
| **ArtP** | **L-Arg ABC transporter ATP-binding subunit** | **Down** | **Up** |
| **YohN (RcnB)** | **Periplasmic protein involved in Ni/Co export** | **Down** | **Up** |
| **OppF** | **Murein tripeptide ABC transporter ATP-binding subunit** | **Up** | **Down** |
| **MalK** | **Maltose/maltodextrin import ATP-binding subunit** | **Up** | **Down** |
| **ArtJ** | **L-Arg ABC transporter periplasmic binding subunit** | **Up** | **Down** |
| **GltK** | **L-Glu/Asp ABC transporter membrane subunit** | **Up** | **Down** |
| **GltL** | **L-Glu/Asp ABC transporter ATP-binding subunit** | **Up** | **Down** |
| **GltJ** | **L- Glu/Asp ABC transporter membrane subunit** | **Up** | **Down** |
| **GlnP** | **L-Gln ABC transporter membrane subunit** | **Up** | **Down** |
| **ProW** | **L-Gly/L-Pro/betaine ABC transporter membrane subunit** | **Up** | **Down** |
| **IsrB*** | **AI2 ABC transporter substrate-binding subunit** | **Up** | **Down** |

| | | | |
|---|---|---|---|
| **YdcS** | **Polyhydroxybutyrate synthase/ABC transporter** | **Up** | **Down** |

1.8.6. The general secretory pathway (Sec) family.

| | | | |
|---|---|---|---|
| **SecA** | **Protein translocase subunit** | **Up** | **Down** |
| **SecD** | **Protein translocase subunit** | **Up** | **Down** |
| **SecY** | **Protein translocase subunit** | **Up** | **Down** |
| **YidC** | **Membrane protein insertase** | **Up** | **Down** |

1.9. Energy metabolism

| | | | |
|---|---|---|---|
| **GlpB** | **Anaerobic glycerol-3-phosphate dehydrogenase** | **Down** | **Up** |
| **GlpD** | **Aerobic glycerol-3-phosphate dehydrogenase** | **Down** | **Up** |
| **GlpK** | **Glycerol kinase** | **Down** | **Up** |
| **NapA** | **Periplasmic nitrate reductase subunit** | **Down** | **Up** |
| **FrdA** | **Fumarate reductase flavoprotein subunit** | **Down** | **Up** |
| **NuoE** | **NADH:quinone oxidoreductase subunit E** | **Down** | **Up** |
| **CcmG** | **Holocytochrome c synthetase** | **Down** | **Up** |
| **ErpA** | **Iron-sulfur cluster insertion protein** | **Up** | **Down** |
| **SufA** | **Iron-sulfur cluster insertion protein** | **Up** | **Down** |
| **YqcA** | **Putative flavodoxin** | **Up** | **Down** |
| **WrbA** | **NAD(P)H dehydrogenase** | **Up** | **Down** |
| **PntA** | **NAD(P) transhydrogenase subunit α** | **Up** | **Down** |
| **PntB** | **NAD(P) transhydrogenase subunit β** | **Up** | **Down** |
| **YdiJ** | **Putative FAD-linked oxidoreductase** | **Up** | **Down** |

| | | | |
|---|---|---|---|
| **SucA** | **Thiamine-requiring 2-oxoglutarate decarboxylase** | **Up** | **Down** |
| **AceE** | **Pyruvate dehydrogenase E1 component** | **Up** | **Down** |
| **AceF** | **Pyruvate dehydrogenase E2 component** | **Up** | **Down** |
| **Tam** | ***Trans*-aconate 2-methyltransferase** | **Up** | **Down** |
| **FdoG** | **Formate dehydrogenase O subunit α** | **Up** | **Down** |
| **CyoB** | **Cytochrome $bo_3$ ubiquinol oxidase subunit 1** | **Up** | **Down** |
| **NuoA** | **NADH:quinone oxidoreductase subunit A** | **Up** | **Down** |
| **NuoH** | **NADH:quinone oxidoreductase subunit H** | **Up** | **Down** |
| **CydA** | **Cytochrome bd-1 ubiquinol oxidase subunit 1** | **Up** | **Down** |

1.10. Glycolate/Glyoxalate degradation.

| | | | |
|---|---|---|---|
| **GlcB** | **Malate synthase G** | **Down** | **Up** |
| **GlcE** | **Glycolate oxidase subunit** | **Up** | **Down** |

1.11. Gluconeogenesis and glycolysis.

| | | | |
|---|---|---|---|
| **Fbp** | **Fructose-1,6-bisphosphatase class 1** | **Down** | **Up** |

1.12. Iron metabolism.

| | | | |
|---|---|---|---|
| **ChuS** | **Hematinate-forming heme oxygenase** | **Down** | **Up** |

1.13. Phosphate acquisition.

| | | | |
|---|---|---|---|
| **AphA** | **Class B acid phosphatase** | **Down** | **Up** |

1.14. Manganese transport.

| | | | |
|---|---|---|---|
| **MntR** | **Manganese transport regulator** | **Down** | **Up** |

1.15. Unclear role in metabolism.

| | | | |
|---|---|---|---|
| **Tas** | **Tyrosine auxotrophy suppressor (aldo-keto reductase)** | **Down** | **Up** |
| **YbiC (HcxB)** | **Hydroxyphenyllactate dehydrogenase B** | **Down** | **Up** |
| **BglA** | **6-Phospho-β-glucosidase A** | **Down** | **Up** |
| **Gss** | **Fused glutathionylspermidine amidase** | **Down** | **Up** |
| **EutL** | **Ethanolamine utilization microcompartment protein** | **Down** | **Up** |
| **YfcE** | **Phosphodiesterase** | **Down** | **Up** |
| **MalM** | **Maltose regulon periplasmic protein** | **Down** | **Up** |
| **YedX (HiuH)** | **Transthyretin-related 5-hydroxyisourate hydrolase** | **Up** | **Down** |
| **HemX** | **Uroporphyrinogen-III methyltransferase** | **Up** | **Down** |
| **HemY** | **Porphyrin biosynthesis protein** | **Up** | **Down** |

2. Central bacterial morphology, and genome, transcriptome and proteome maintenance.

2.1. Capsule and LPS biosynthesis

| | | | |
|---|---|---|---|
| **NanR** | **Repressor of the sialic acid metabolism and transport** | **Down** | **Up** |
| **RfbB** | **dTDP-glucose 4,6-dehydratase** | **Down** | **Up** |
| **RmlB** | **dTDP-glucose 4,6-dehydratase** | **Down** | **Up** |
| **RffG** | **dTDP-glucose 4,6-dehydratase** | **Down** | **Up** |
| **KdsA** | **2-dehydro-3-deoxyphosphooctonate aldolase** | **Down** | **Up** |
| **RfaE** | **Fused heptose 7-P kinase/heptose 1-P adenyltransferase** | **Up** | **Down** |
| **KpsE** | **Capsule polysaccharide export inner-membrane protein** | **Up** | **Down** |
| **MrcB** | **Penicillin-binding protein 1Bγ** | **Up** | **Down** |

| | | | |
|---|---|---|---|
| **DacC** | **D-Ala-D-alanine carboxypeptidase (PBP6)** | **Up** | **Down** |
| **MlaD** | **Subunit of the retrograde phospholipid trafficking system** | **Up** | **Down** |
| **MsbA** | **Lipid A export ATP-binding/permease** | **Up** | **Down** |
| **Blc** | **Outer membrane lipoprotein** | **Up** | **Down** |
| **Lgt** | **Prolipoprotein diacylglyceryl transferase** | **Up** | **Down** |
| **YgdI** | **DUF903 domain lipoprotein** | **Up** | **Down** |
| **MscS** | **Small-conductance mechanosensitive channel** | **Up** | **Down** |

2.2. Genome maintenance

| | | | |
|---|---|---|---|
| **Fis** | **DNA-binding protein** | **Down** | **Up** |
| **MukE** | **Chromosome partition protein** | **Down** | **Up** |
| **EngB** | **Chromosome partition, GTP-binding protein** | **Down** | **Up** |
| **IhfB** | **Integration host factor β** | **Down** | **Up** |
| **IhfA** | **Integration host factor α** | **Up** | **Down** |
| **H-NS** | **DNA-binding protein** | **Up** | **Down** |
| **GyrB** | **DNA gyrase subunit B** | **Up** | **Down** |
| **PolA** | **DNA polymerase I** | **Up** | **Down** |
| **HolD** | **DNA polymerase III subunit ψ** | **Up** | **Down** |

2.3. Transcriptome maintenance.

2.3.1. Transcriptional factors and RNA polymerase sigma subunits

| | | | |
|---|---|---|---|
| **RpoA** | **RNA polymerase α-subunit** | **Down** | **Up** |
| **Rsd** | **Regulator of sigma D** | **Down** | **Up** |

| | | | |
|---|---|---|---|
| **CspA** | **Cold shock protein A** | **Up** | **Down** |
| **CspE** | **Cold shock protein E** | **Up** | **Down** |
| **RpoB** | **RNA polymerase subunit β** | **Up** | **Down** |
| **RpoC** | **RNA polymerase subunit β'** | **Up** | **Down** |

2.3.2. Ribonucleases.

| | | | |
|---|---|---|---|
| **Rna** | **Ribonuclease I** | **Down** | **Up** |
| **Rnc** | **Ribonuclease 3** | **Down** | **Up** |
| **Rnr** | **Ribonuclease R** | **Up** | **Down** |
| **Pnp** | **Polynucleotide phosphorylase** | **Up** | **Down** |
| **YdiZ** | **Putative endoribonuclease** | **Up** | **Down** |
| **RraB (YjgD)** | **Regulator of ribonuclease activity B** | **Up** | **Down** |
| **RhlB** | **ATP-dependent RNA helicase** | **Up** | **Down** |

2.3.3. RNA-binding proteins.

| | | | |
|---|---|---|---|
| **YbcJ** | **Putative RNA-binding protein** | **Down** | **Up** |

2.4. Proteome maintenance.

2.4.1. Ribosome assembly and activity.

| | | | |
|---|---|---|---|
| **RplF** | **50S ribosomal protein L6** | **Down** | **Up** |
| **RpmD** | **50S ribosomal protein L30** | **Down** | **Up** |
| **RpsG** | **30S ribosomal protein S7** | **Down** | **Up** |
| **RpsP** | **30S ribosomal protein S16** | **Down** | **Up** |
| **RpsS** | **30S ribosomal protein S19** | **Down** | **Up** |

| | | | |
|---|---|---|---|
| **RpsU** | **30S ribosomal protein S21** | **Down** | **Up** |
| **YjfH (RimB)** | **23S rRNA(guanosine-2-O-)-methyltransferase** | **Down** | **Up** |
| **TypA (YihK)** | **Ribosome-dependent GTP-ase assembly factor** | **Down** | **Up** |
| **YfiA (RaiA)** | **Ribosome-associated inhibitor A** | **Down** | **Up** |
| **RsgA** | **Putative ribosome biogenesis GTP-ase** | **Down** | **Up** |
| **Era** | **30S ribosomal subunit maturation GTPase** | **Down** | **Up** |
| **YceD** | **23S rRNA accumulation protein** | **Down** | **Up** |
| **RpsC** | **30S ribosomal protein S3** | **Up** | **Down** |
| **RpsD** | **30S ribosomal protein S4** | **Up** | **Down** |
| **RplP** | **50S ribosomal protein L6** | **Up** | **Down** |
| **RplT** | **50S ribosomal protein L20** | **Up** | **Down** |
| **InfB** | **Translation intiation factor IF-2** | **Up** | **Down** |
| **YggL** | **Putative ribosome assembly factor** | **Up** | **Down** |
| **HflX** | **Heat shock-induced ribosome-dependent GTP-ase** | **Up** | **Down** |
| **YihI** | **Der GTP-ase activating protein** | **Up** | **Down** |
| **YqjD** | **Ribosome- and membrane-associated DUF883 protein** | **Up** | **Down** |
| **YjjK (EttA)** | **Energy-dependent translational throttle protein** | **Up** | **Down** |

2.4.2. tRNA ligases and tRNA modification enzymes.

| | | | |
|---|---|---|---|
| **HisS** | **Histindine-tRNA ligase** | **Down** | **Up** |
| **SerS** | **Serine-tRNA ligase** | **Down** | **Up** |
| **TyrS** | **Tyrosine-tRNA ligase** | **Down** | **Up** |

| | | | |
|---|---|---|---|
| **Tgt** | **Queunine tRNA-ribosyltransferase** | **Down** | **Up** |
| **TrmJ (YfhQ)** | **tRNA(cytidine/uridine-2-O-)-methyltransferase** | **Down** | **Up** |
| **TrmD** | **tRNA(guanidine-N(1)-)-methyltransferase** | **Down** | **Up** |
| **DusA** | **tRNA-dihydrouridine synthase** | **Up** | **Down** |
| **GlyS** | **Glycine-tRNA ligase beta subunit** | **Up** | **Down** |
| **YfiF** | **tRNA/rRNA methyltransfrase** | **Up** | **Down** |
| **PheT** | **Phenylalanine-tRNA ligase β subunit** | **Up** | **Down** |

2.4.3. Chaperones and proteases.

| | | | |
|---|---|---|---|
| **IbpA** | **Small heat shock protein** | **Down** | **Up** |
| **ClpA** | **ATP-binding subunit of Clp** | **Down** | **Up** |
| **OmpT** | **Protease 7** | **Down** | **Up** |
| **PepD** | **Peptidase D** | **Down** | **Up** |
| **PepN** | **Aminopeptidase N** | **Down** | **Up** |
| **PepQ** | **Xaa-Pro dipeptidase** | **Down** | **Up** |
| **PepT** | **Peptidase T** | **Down** | **Up** |
| **PepA** | **Aminopeptidase A/I and transcriptional repressor** | **Up** | **Down** |
| **PepB** | **Peptidase B** | **Up** | **Down** |
| **PepP** | **Proline aminopeptidase II** | **Up** | **Down** |
| **YggG (LoiP)** | **Metalloprotease** | **Up** | **Down** |
| **DegP** | **Periplasmic serine endopeptidase** | **Up** | **Down** |
| **DegQ** | **Periplasmic pH-dependent serine endopeptidase** | **Up** | **Down** |

| | | | |
|---|---|---|---|
| **HslU** | **ATP-dependent protease ATP subunit** | **Up** | **Down** |
| **FtsH (HflB)#** | **ATP-dependent zinc metalloprotease** | **Up** | **Down** |
| **HflC** | **Modulator of FtsH protease** | **Up** | **Down** |
| **HflK** | **Regulator of FtsH protease** | **Up** | **Down** |
| **FklB** | **Peptidyl-prolyl *cis-trans* isomerase** | **Up** | **Down** |
| **GroEL** | **Chaperonin** | **Up** | **Down** |

2.5. Cell division.

| | | | |
|---|---|---|---|
| **FtsA** | **Cell division protein that anchors the Z ring** | **Up** | **Down** |
| **YfdQ#** | **Prophage protein, part of the *had-185ΔsulA* suppressor** | **Up** | **Down** |
| **DamX #** | **Cell division protein** | **Up** | **Down** |
| **BolA** | **DNA-binding transcriptional regulator** | **Up** | **Down** |
| **MreB** | **Dynamic cytoskeletal protein** | **Up** | **Down** |
| **MraZ (YabB)#** | **Cell wall and cell division transcriptional repressor** | **Up** | **Down** |
| **YhcB#** | **DUF1043 domain-containing inner membrane protein** | **Up** | **Down** |

2.6. Miscellaneous factors.

| | | | |
|---|---|---|---|
| **Mrp** | **P-loop NTP-ase family protein** | **Down** | **Up** |
| **ECOK1_1466*** | **Class I SAM-dependent methyltransferase** | **Down** | **Up** |
| **ECOK1_4263** | **SAM:hydroxide methyltransferase** | **Down** | **Up** |
| **YigI** | **Putative thioesterase** | **Down** | **Up** |

3. Factors involved in resistance and survival strategies.

3.1. DNA SOS stress response.

| | | | |
|---|---|---|---|
| **RecR** | **Recombination protein** | **Down** | **Up** |
| **YmfK** | **e14 lambdoid LexA-like regulator** | **Down** | **Up** |
| **ObgE#** | **Chromosome partitioning and ribosome assembly GTP-ase** | **Up** | **Down** |
| **YafO#** | **Ribosome-dependent mRNA interferase toxin** | **Up** | **Down** |

3.2. DNA repair.

3.2.1. Mismatch repair.

| | | | |
|---|---|---|---|
| **SbcB (ExoI)** | **Exodeoxyribonuclease I** | **Down** | **Up** |

3.2.2. Base-excision repair

| | | | |
|---|---|---|---|
| **MutT** | **3-oxo-dGTP diphosphatase** | **Down** | **Up** |

3.3. Envelope stress response.

| | | | |
|---|---|---|---|
| **CpxP** | **Negative regulator of the Cpx pathway; chaperone** | **Down** | **Up** |
| **Mpl (YjfG)** | **Murein peptide ligase** | **Down** | **Up** |
| **MepA** | **Penicillin-insensitive murein endopeptidase** | **Down** | **Up** |
| **YfeY** | **Lipoprotein; a member of the $\sigma^E$ regulon** | **Up** | **Down** |
| **YajI** | **Lipoprotein** | **Up** | **Down** |
| **UspC (YecG)** | **Universal stress protein** | **Up** | **Down** |

3.4. Oxidative stress.

| | | | |
|---|---|---|---|
| **FrmB** | **S-formylglutathione hydrolase** | **Down** | **Up** |
| **YfiD (GrcA)** | **Autonomous glycyl radical cofactor** | **Down** | **Up** |
| **YaaA** | **DNA-binding and ROS stress response protein** | **Down#** | **Up** |
| **YbgL (PxpA)** | **5-oxo-prolinase component A** | **Up** | **Down** |

| | | | |
|---|---|---|---|
| **BtuE** | **Glutathione peroxidase** | **Up** | **Down** |
| **OsmC** | **Osmotically inducible peroxiredoxin** | **Up** | **Down** |
| **CueO** | **Multicopper oxidase** | **Up** | **Down** |
| **MsrP (YedY)** | **Protein-L-methionine sulfoxide reductase** | **Up** | **Down** |
| **KatG** | **Catalase/Peroxidase HPI** | **Up** | **Down** |

3.5. Sessility/motility switches.

| | | | |
|---|---|---|---|
| **YahA (PdeL)** | **DNA-binding activator/c-di-GMP phosphodiesterase** | **Down** | **Up** |
| **YedQ (DgcQ)** | **Diguanylate cyclase** | **Up** | **Down** |

3.6. Other factors.

| | | | |
|---|---|---|---|
| **Can** | **Carbonic anhydrase** | **Down** | **Up** |
| **TehB** | **Tellurite methyltransferase** | **Down** | **Up** |
| **YdhQ** | **Adhesin-related protein** | **Down** | **Up** |
| **Clb** | **Colibactin biosynthesis thioesterase** | **Down** | **Up** |
| **MdoD** | **Osmoregulated periplasmic glucan biosynthesis protein D** | **Down** | **Up** |
| **MdoG (OpgG)** | **Osmoregulated periplasmic glucan biosynthesis protein G** | **Down** | **Up** |
| **UspF** | **Nucleotide binding filament protein** | **Up** | **Down** |
| **YbeD** | **DUF493 protein** | **Up** | **Down** |
| **MsyB** | **Acidic protein** | **Up** | **Down** |
| **YbjQ** | **UPF0145 putative heavy metal binding protein** | **Up** | **Down** |
| **FimC** | **Type-1 fimbrial chaperone** | **Up** | **Down** |

| | | | |
|---|---|---|---|
| 4. Proteins with unknown function | | | |
| **YfbU** | | **Down** | **Up** |
| **YcjX** | **Induced under N starvation** | **Down** | **Up** |
| **EcfF (YggN)** | **DUF2884 domain-containing protein** | **Up** | **Down** |
| **YbeL** | **DUF1451 domain-containing protein** | **Up** | **Down** |
| **ECOK1_3003** | **Prophage conserved protein** | **Up** | **Down** |
| **YebY** | **DUF2511 domain-containing protein** | **Up** | **Down** |
| **YfcL** | **PF08891 family protein** | **Up** | **Down** |
| **YccJ** | **PF13993 family protein** | **Up** | **Down** |

## Footnotes

[1] Classification as Up and Down is based on the fold change with (FC): FC >1.5 as upregulated and FC < 1/1.5=0.667 as downregulated.

[2] AIR: 5-amino-1-(5-phospho-D-ribosyl)imidazole.

[3] GAR: glycinamide ribonucleotide.

**Table S2. List of proteins at levels below detection in one of the two strains.**

**A.** Undetected proteins in IHE3034

| **Gene** | **Protein** | **IHE3034** | **RS218** |
|---|---|---|---|
| *dinD* | DNA damage-inducible protein D | 0 | 789800 |
| *ytfM* (*tamA*) | Ag43 translocation and assembly module subunit | 0 | 1103200 |
| *chaA* | $Na^+/K^+:H^+$ antiporter | 0 | 1289380 |
| *fyuA* | Yersiniabactin receptor | 0 | 1346220 |
| *yfcN* | UPF0115 protein | 0 | 1561340 |
| *sohB* | S49 peptidase family protein | 0 | 2010800 |
| *iclR* | *ace* operon repressor | 0 | 2024200 |
| *yjgR* | DUF853 domain-containing protein | 0 | 2104600 |
| *ycfS* | L,D-transpeptidase | 0 | 2127400 |
| *yfiC* | tRNA1(Val) (adenine(37)-N6)-methyltransferase | 0 | 2178400 |
| *yfgB* | Dual-specificity RNA methyltransferase RlmN | 0 | 2227600 |
| ERS451419_03394 | | 0 | 2275400 |
| *ptrB* | Oligopeptidase B | 0 | 2309800 |
| *gcp* | tRNA N6-adenosine threonylcarbamoyltransferase | 0 | 2364400 |
| *chaC* | Glutathione-specific γ-glutamylcyclotransferase | 0 | 2579400 |
| *ybdG* | Miniconductance mechanosensitive channel | 0 | 2643600 |
| *glpE* | Thiosulfate sulfurtransferase GlpE | 0 | 2736800 |
| *caiD* | Carnitinyl-CoA dehydratase | 0 | 2834400 |
| *yheV* | DUF2387 domain-containing protein | 0 | 2846800 |
| *fliG* | Flagellar motor switch protein FliG | 0 | 2995200 |
| *cheY* | Chemotaxis protein CheY | 0 | 3184000 |
| *acpH* | Acyl carrier protein phosphodiesterase | 0 | 3279600 |
| *bioD* | ATP-dependent dethiobiotin synthetase BioD | 0 | 3301800 |
| *yjeF* (*nnr*) | NAD(P)HX epimerase / NAD(P)HX dehydratase | 0 | 3326200 |
| *proB* | Glutamate 5-kinase | 0 | 3345000 |
| *ybjD* | DUF2813 domain-containing protein | 0 | 3549400 |
| *yfjG* (*ratA*) | Ribosome association toxin | 0 | 3600600 |
| *dnaT* | Primosomal protein 1 | 0 | 3628400 |
| *yeiR* | Zinc-binding GTPase | 0 | 3830200 |
| *sdhD* | Succinate:quinone oxidoreductase | 0 | 3895800 |
| *ypfG* | DUF1176 domain-containing protein | 0 | 3958000 |
| *ebgR* | Evolved β-galactosidase repressor | 0 | 4169200 |
| *clbG* | Colibactin biosynthesis acyltransferase | 0 | 4270800 |
| ECS88_1341 | Hypothetical protein | 0 | 4498000 |
| *tusB* (*dsrH*) | Sulfurtransferase (tRNA-U thiolation/selenation) | 0 | 4635600 |
| *coaBC* (*dfp*) | Fused 4'-phosphopantothenoylcysteine decarboxylase and phosphopantothenoylcysteine synthetase | 0 | 4692200 |
| *ydfI* | Putative oxidoreductase | 0 | 4693800 |
| *mdoH* (*opgH*) | Glucans biosynthesis glucosyltransferase H | 0 | 4935600 |
| *zntA* (*cadA*) | $Zn^{2+}/Cd^{2+}/Pb^{2+}$ translocating P-type ATPase | 0 | 5041800 |
| *mukB* | Chromosome partition protein MukB | 0 | 5293200 |

| | | | |
|---|---|---|---|
| *ygiS* | Putative deoxycholate binding periplasmic protein | 0 | 5334400 |
| *fucI* | L-fucose isomerase | 0 | 5502800 |
| *yhbJ* (*rapZ*) | RNase adapter protein | 0 | 5541000 |
| *envC* | Murein hydrolase activator | 0 | 5670000 |
| *rpoN* | RNA polymerase sigma-54 factor | 0 | 5706400 |
| *yhaJ* | LysR-like transcriptional regulator | 0 | 5887000 |
| *yjfK* | DUF2491 domain-containing protein | 0 | 6076000 |
| ECS88_3204 | DNA-binding protein, DUF296 domain-containing protein | 0 | 6135800 |
| *ubiX* | Flavin prenyltransferase | 0 | 6156000 |
| *yafJ* | Putative glutamine amidotransferase | 0 | 6204800 |
| *mutS* | DNA mismatch repair protein | 0 | 6258000 |
| *visC* (*ubiI*) | 2-octaprenylphenol 6-hydroxylase | 0 | 6293200 |
| *kduI* | 4-deoxy-L-threo-5-hexosulose-uronate ketol-isomerase | 0 | 6332800 |
| *kdtA* | KDO transferase | 0 | 6336200 |
| *rimL* | Ribosomal-protein-L12-serine N-acetyltransferase | 0 | 6684600 |
| *yaeP* | UPF0253 protein | 0 | 6805400 |
| *aapJ* (*gltI*) | Glu/Asp ABC transporter periplasmic binding protein | 0 | 6815200 |
| *stbA* | Par-like plasmid-based | 0 | 6818800 |
| *fepA* | $Fe^{3+}$ enterobactin outer membrane transporter | 0 | 6880400 |
| *leuD* | 3-isopropylmalate dehydratase small subunit | 0 | 6965000 |
| *rpsL* | 30S ribosomal protein S12 | 0 | 7282200 |
| *thiQ* | Thiamine import ATP-binding protein | 0 | 7382200 |
| *yfhM* | $\alpha_2$-macroglobulin | 0 | 7459200 |
| *yijP* | Phosphoethanolamine transferase EptC | 0 | 7463600 |
| *cydB* | Cytochrome bd-I ubiquinol oxidase subunit II | 0 | 7590000 |
| *ybcH* | DUF4434 domain-containing protein | 0 | 7612400 |
| CE10_1285 | Hypothetical protein | 0 | 7616600 |
| *yihR-like* | Putative sulfoquinovose aldose-1-epimerase | 0 | 7715800 |
| *mreC* | Cell shape determining protein | 0 | 7739000 |
| *Irp* | Leucine-responsive regulatory protein | 0 | 7798800 |
| ECS88_1342 (ECOK1_1424) | Hypothetical protein | 0 | 7915800 |
| *ppk* | Polyphosphate kinase | 0 | 7933600 |
| *rnd* | Ribonuclease D | 0 | 8150800 |
| *nhoA* | Arylamine N-acetyltransferase | 0 | 8281200 |
| *secF* | Protein-export membrane protein SecF | 0 | 8390600 |
| *selA* | L-seryl-tRNA(Sec) selenium transferase | 0 | 8453800 |
| *xylA* | Xylose isomerase | 0 | 8507800 |
| ECS88_1212 | Hypothetical protein | 0 | 8545200 |
| *mltC* | Membrane-bound lytic murein transglycosylase C | 0 | 8639800 |
| *ydhB* | Putative LysR family DNA-binding transcriptional regulator | 0 | 9012000 |
| *ycjW* | Putative LacI-type DNA-binding transcriptional factor | 0 | 9256000 |
| *fruR* (*cra*) | Catabolite repressor activator | 0 | 9301000 |

| | | | |
|---|---|---|---|
| *rfaH* | Transcription antitermination protein RfaH | 0 | 9403200 |
| *treB* | PTS system trehalose-specific EIIBC component | 0 | 9945200 |
| CE10_4492 | IclR family transcriptional regulator | 0 | 10133200 |
| CE10_1277 | DUF2190 domain-containing protein (present in RS218) | 0 | 10185600 |
| *flgA* | Flagellar basal body P-ring formation protein | 0 | 10284000 |
| *murC* | UDP-N-acetylmuramate--L-alanine ligase | 0 | 10558000 |
| *hemG* | Protoporphyrinogen oxidase | 0 | 10669000 |
| *yoaE* | Putative inner membrane protein | 0 | 10812000 |
| *glpX* | Fructose-1,6-bisphosphatase | 0 | 11047200 |
| *fdoH* | Formate dehydrogenase O subunit β | 0 | 11510200 |
| *yibT* | Small ORF protein | 0 | 11651800 |
| *yagV* (*matF*) | Probable fimbrial chaperone EcpE | 0 | 11738200 |
| *btuB* | Vitamin B12 transporter | 0 | 12771600 |
| *greB* | Transcription elongation factor | 0 | 13027600 |
| *trmC* | tRNA 5-methylaminomethyl-2-thiouridine biosynthesis bifunctional protein MnmC | 0 | 13038600 |
| *rsmD* | 16S rRNA m2G966 methyltransferase D | 0 | 13115400 |
| *yeaG* | Protein kinase | 0 | 14461000 |
| *frdC* | Fumarate reductase subunit C | 0 | 14678000 |
| *yiaJ* (*plaR*) | L-lyxose metabolism transcriptional repressor | 0 | 14765200 |
| *ybaW* (*fadM*) | Long-chain acyl-CoA thioesterase III | 0 | 15546400 |
| *yncA* (*mnaT*) | L-amino acid N-acyltransferase | 0 | 16059600 |
| *ampC* | Beta-lactamase | 0 | 16105200 |
| ECS88_3886; ECOK1_3912 | Fructose-specific PTS transporter subunit IIA | 0 | 16644400 |
| *lipB* | Octanoyltransferase | 0 | 17118800 |
| *rapA* | RNA polymerase-binding ATPase and RNAP recycling factor | 0 | 18260800 |
| *ampE* | Putative regulator of AmpC | 0 | 20245800 |
| *ycaR* | UPF0434 protein | 0 | 22098000 |
| *ybbH* | Hypothetical NanR-like protein | 0 | 23213800 |
| *mglA* | Galactose/methyl galactoside import ATP-binding protein | 0 | 24531600 |
| *pstB* | Phosphate import ATP-binding protein | 0 | 25879400 |
| *ydiV* (*rflP*) | anti-FlhDC factor | 0 | 25981200 |
| *yvgN_1* (*dkgA*) | Methylglyoxal reductase | 0 | 26105800 |
| *proC* | Pyrroline-5-carboxylate reductase | 0 | 26490800 |
| *lolC* | Lipoprotein release complex - inner membrane subunit | 0 | 29187400 |
| *mfd* | Transcription-repair-coupling factor | 0 | 33287800 |
| *ygbM* | Hydroxypyruvate isomerase | 0 | 36093200 |
| *betA* | Oxygen-dependent choline dehydrogenase | 0 | 39446000 |
| *senB* | Enterotoxin TieB protein; plasmid-based gene | 0 | 43139200 |
| *ymjC* | Putative uncharacterized protein | 0 | 47630000 |
| *exbB* | Ton complex subunit | 0 | 50172600 |
| CE10_1750 | Hypothetical protein | 0 | 54722800 |
| *yfeC* | Putative DNA-binding transcriptional regulator | 0 | 55140000 |

| | | | |
|---|---|---|---|
| *yghZ* (*gpr*) | L-glyceraldehyde 3-phosphate reductase | 0 | 57956800 |
| *cydC* | Glutathione/L-cysteine ABC exporter subunit | 0 | 63170000 |
| *relA* | GDP/GTP pyrophosphokinase | 0 | 63500000 |
| CE10_A10 | NADH:ubiquinone reductase (Na(+)-transporting) subunit C | 0 | 81910400 |
| *nudK* | GDP-mannose pyrophosphatase | 0 | 101766000 |
| *cycA* | D-serine/alanine/glycine/:H+symporter | 0 | 105464000 |
| ECS88_2204 | Hypothetical protein (phage region) | 0 | 145822000 |
| ECS88_0539 (*dicA*) | DNA-binding transcriptional dual regulator | 0 | 153626000 |
| CE10_A16 | ABC transporter ATP-binding protein | 0 | 188746000 |
| *yggE* | DUF541 domain-containing protein | 0 | 228080000 |
| CE10_A52 (ParA) | Chromosome partitioning protein | 0 | 334988000 |
| *ccdB* | Type II toxin CcdB | 0 | 418264000 |
| CE10_A51 | Hypothetical protein | 0 | 421230000 |
| *traT* | Complement resistance protein | 0 | 505274000 |
| CE10_A12 | Iron transporter | 0 | 1057688000 |
| *dcp* | Dipeptidyl carboxypeptidase II | 0 | 3006640000 |

**B.** Undetected proteins in RS218

| **Gene** | **Protein** | **IHE3034** | **RS218** |
|---|---|---|---|
| *csqR* (*yihW*) | Putative transcriptional repressor for genes of catabolism of sulfoquinovose | 3918420 | 0 |
| *ybjK* (*rcdA*) | TetR-like transcriptional regulator | 1877140 | 0 |
| *mpaA* | Murein tripeptide amidase A | 10875600 | 0 |
| *pncA* | Nicotinamidase | 75692400 | 0 |
| *tdcG* | L-serine deaminase III | 9921200 | 0 |
| CE10_2382 | XRE family transcriptional regulator | 23665000 | 0 |
| *Tag* | 3-methyl-adenine DNA glycosylase I | 49668800 | 0 |
| *endA* | DNA-specific endonuclease I | 3955400 | 0 |
| ECS88_4791 | Type II toxin-antitoxin system HipA family toxin | 90834800 | 0 |
| *ydaL* (*smrA*) | DNA endonuclease | 6363600 | 0 |
| *gatR* | DNA-binding transcriptional repressor | 7988800 | 0 |
| ECS88_4576 | ABC transporter substrate-binding protein | 30270200 | 0 |
| ECS88_3996 | DUF4862 domain-containing protein | 8638800 | 0 |
| *lpxC* | UDP-3-O-[3-hydroxymyristoyl] N-acetylglucosamine deacetylase | 5260000 | 0 |
| *siiEA* | Putative adhesion | 19842800 | 0 |
| *Dxr* | 1-deoxy-D-xylulose 5-phosphate reducto-isomerase | 66296800 | 0 |
| *ybaV* | Uncharacterized protein | 15875400 | 0 |
| ECS88_0596 | Putative exported protein; putative protease | 160680000 | 0 |
| *dmsB* | Anaerobic dimethyl sulfoxide reductase chain B | 6316400 | 0 |
| *minE* | Cell division topological specificity factor | 3643580000 | 0 |
| *trpE* | Anthranilate synthase component 1 | 12862000 | 0 |
| *ycfL* | Uncharacterised protein | 31069600 | 0 |
| *ymgD* | Uncharacterised protein | 3513600 | 0 |
| *Mind* | Site-determining protein | 2049940000 | 0 |
| *Mlc* | Transcriptional repressor of MalT | 8996000 | 0 |
| *sufE* | Cysteine desulfuration protein | 87081400 | 0 |
| *rsmF* | Ribosomal RNA small subunit methyltransferase F | 3681000 | 0 |

| | | | |
|---|---|---|---|
| *yobB* | Putative carbon-nitrogen hydrolase | 12566000 | 0 |
| *nuoM* | NADH:quinone oxidoreductase subunit M | 15090400 | 0 |
| *lrhA* | DNA-binding transcriptional dual regulator | 9040800 | 0 |
| *yehT* | DNA-binding transcriptional dual regulator | 6423600 | 0 |
| CE10_2924 | XRE family transcriptional regulator | 1705260000 | 0 |
| *sdaB* | L-serine deaminase II | 75982200 | 0 |
| *hypD* | Fe-$(CN)_2$CO cofactor assembly scaffold protein | 2048200 | 0 |
| *had* | DnaA regulatory inactivator | 14720600 | 0 |
| CE10_2893 | Hypothetical protein | 1250420000 | 0 |
| *argP* | HTH-type transcriptional regulator | 13868200 | 0 |
| *kduD* | Putative 2-keto-3-deoxy-D-gluconate dehydrogenase | 11655800 | 0 |
| *rlmD* | 23S rRNA (uracil(1939)-C(5))-methyltransferase | 4682200 | 0 |
| *rrmJ* | Ribosomal RNA large subunit methyltransferase E | 107496400 | 0 |
| *ygiB* | UPF0441 protein | 5580564 | 0 |
| *malQ* | 4-alpha-glucanotransferase | 3970200 | 0 |
| *rfaC* | ADP-heptose:LPS heptosyl transferase I | 19559800 | 0 |
| *hdeB* | Acid stress chaperone | 5808800 | 0 |
| *malS* | α – amylase | 10189000 | 0 |
| *bcsC* | Cellulose synthase outer membrane channel | 26928000 | 0 |
| *yjaH* | DUF1481 protein | 30287000 | 0 |
| *mnmG* | tRNA uridine 5-carboxymethylaminomethyl modification enzyme | 9187400 | 0 |
| *yjgM* | Putative acetyltransferase | 6057400 | 0 |
| *Nfi* | Endonuclease V | 15442800 | 0 |
| *zraR* | DNA-binding transcriptional activator | 20106000 | 0 |
| *cutA* | Divalent-cation tolerance protein CutA | 6154800 | 0 |
| *rimI* | N-acetyltransferase RimI | 3665200 | 0 |
| *Folk* | 2-amino-4-hydroxy-6-hydroxymethyldihydropteridine diphosphokinase | 57153000 | 0 |
| *ybbA* | Putative ABC transporter ATP-binding protein | 27681600 | 0 |
| *lacI* | Transcriptional repressor of the lac operon | 60249800 | 0 |
| *holB* | DNA polymerase III subunit δ' | 24750400 | 0 |
| *cdtC* | Cytolethal distending toxin type IV subunit C | 4368680000 | 0 |
| *yddE* | PF02567 family protein | 16191400 | 0 |
| *cdtA* | Cytolethal distending toxin type IV subunit A | 2054640000 | 0 |
| *ydbA* | Putative autotransporter protein | 7500600 | 0 |
| *ydhA* | Inhibitor of c-type lysozyme | 101652000 | 0 |
| *cdtB* | Cytolethal distending toxin type IV subunit B | 12800200000 | 0 |
| *pbpC* | Peptidoglycan glycosyltransferase | 7218000 | 0 |
| *kpsF* | Arabinose 5-phosphate isomerase | 79289400 | 0 |
| *chiA* | Periplasmic endochitinase | 2450800 | 0 |
| *bisC* | Biotin sulfoxide reductase | 8071600 | 0 |
| *yieH* (*hxpA*) | Hexitol phosphatase A | 292982000 | 0 |
| *hdfR* | HTH-type transcriptional regulator HdfR | 33955800 | 0 |
| *sucA* | 2-oxoglutarate decarboxylase | 12139200 | 0 |
| *fadJ* | Fatty acid oxidation complex subunit alpha | 1167000 | 0 |
| *mlaB* | Intermembrane phospholipid transport system protein | 78303000 | 0 |

**Table S3. List of all identified metabolites (negative data, FC = $[RS218]_{metabolite}$/ $[IHE3034]_{metabolite}$)**

| Compound | RT [min] | m/z | MW | Mono isotopic Mass | IHE_1 | IHE_2 | IHE_3 | IHE_4 | IHE_5 | RS_1 | RS_2 | RS_3 | RS_4 | RS_5 | Log2(FC) | Log10 (p-value) | VIP |
|---|---|---|---|---|---|---|---|---|---|---|---|---|---|---|---|---|---|
| L-Valine | 0.957 | 116.0706 | 117.07782 | 117.079 | 217.1183 | 217.212 | 231.4436 | 280.9228 | 291.8025 | 1369.119 | 1099.043 | 1332.172 | 1091.19 | 1583.241 | 2.386235 | 5.439251 | 1.95592 |
| N-acetyl-L-glutamic acid | 1.418 | 188.0559 | 189.0631 | 189.0637 | 79.70088 | 93.00043 | 89.2288 | 102.8773 | 139.1002 | 624.6454 | 321.9407 | 518.1284 | 441.0894 | 513.5417 | 2.263386 | 4.163724 | 1.88718 |
| Ophthalmic acid | 1.14 | 288.1203 | 289.12769 | 289.1274 | 2324.278 | 2074.414 | 2017.774 | 2110.099 | 1567.311 | 3741.608 | 3586.845 | 2959.134 | 3030.619 | 3703.919 | 0.753931 | 3.768218 | 1.85304 |
| Mannitol 1-phosphate | 0.731 | 261.0383 | 262.04555 | 262.0454 | 175.7987 | 218.7958 | 281.8651 | 258.9631 | 416.1923 | 543.9606 | 506.3193 | 524.5204 | 567.487 | 552.1921 | 0.995322 | 3.671305 | 1.84341 |
| Deoxyribose | 1.143 | 133.0496 | 134.05689 | 134.0579 | 1472.742 | 1482.128 | 1648.89 | 1120.399 | 972.8067 | 518.3301 | 436.519 | 313.6276 | 366.8711 | 652.8223 | -1.54931 | 3.649301 | 1.83918 |
| Mevalonic acid | 1.604 | 147.0654 | 148.07266 | 148.0736 | 1364.451 | 1506.868 | 1470.954 | 1761.109 | 1759.767 | 993.8807 | 916.571 | 881.352 | 866.5325 | 1190.338 | -0.69752 | 3.510942 | 1.8242 |
| Lactaldehyde | 0.822 | 73.02816 | 74.03569 | 74.03678 | 1737.461 | 1373.548 | 1415.879 | 1426.774 | 1123.143 | 715.9003 | 787.9743 | 611.6176 | 617.3241 | 972.3754 | -0.93355 | 3.345046 | 1.80453 |
| D-Alanyl-D-alanine | 0.819 | 159.0768 | 160.08403 | 160.0848 | 399.4548 | 359.0461 | 315.808 | 316.1497 | 221.1236 | 595.6091 | 759.4874 | 521.8459 | 609.9103 | 883.9073 | 1.064596 | 2.932528 | 1.75017 |
| N-Acetyl-D-leucine | 4 | 172.0971 | 173.10445 | 173.1052 | 816.4438 | 615.1771 | 520.1109 | 512.8618 | 489.5921 | 1251.233 | 2053.551 | 1426.13 | 1036.442 | 1549.29 | 1.308422 | 2.875221 | 1.73744 |
| Glutamylhydroxyproline | 1.519 | 259.0938 | 260.10104 | 260.1008 | 164.0936 | 177.9864 | 158.5529 | 157.7144 | 95.47232 | 284.6016 | 393.9815 | 269.608 | 224.2608 | 377.7841 | 1.040197 | 2.683047 | 1.70484 |
| L-Leucine | 1.488 | 130.0862 | 131.09357 | 131.0946 | 1600.877 | 2068.06 | 1603.638 | 2650.965 | 2195.789 | 3470.465 | 2780.744 | 4150.389 | 3164.993 | 4407.976 | 0.828844 | 2.609556 | 1.69208 |
| 3-Methylhippuric acid | 4.259 | 192.066 | 193.07338 | 193.0739 | 61.6793 | 68.63817 | 65.7389 | 92.25199 | 78.56468 | 57.9474 | 44.41882 | 34.4895 | 50.3758 | 41.94729 | -0.67881 | 2.447947 | 1.65752 |
| Uridine 5'-diphosphate | 0.706 | 402.9952 | 404.00246 | 404.0022 | 999.2569 | 1047.899 | 1629.151 | 1320.22 | 1192.989 | 1707.089 | 1642.59 | 1922.243 | 1789.899 | 2250.35 | 0.589291 | 2.405533 | 1.64897 |
| Methylisocitric acid | 1.207 | 205.0349 | 206.04221 | 206.0427 | 4933.142 | 5554.663 | 6893.398 | 7616.957 | 8283.818 | 4521.573 | 4099.443 | 3564.699 | 3604.395 | 4441.565 | -0.71813 | 2.38826 | 1.64277 |
| Hydroxyphenyllactic acid | 2.951 | 181.05 | 182.05725 | 182.0579 | 168.4029 | 175.9471 | 164.2794 | 272.6548 | 244.9589 | 154.5439 | 123.2183 | 98.88788 | 98.84727 | 120.375 | -0.7843 | 2.091463 | 1.56895 |
| N-Acetylneuraminic acid | 0.781 | 346.056 | 309.10663 | 309.106 | 1168.628 | 1116.085 | 1484.617 | 953.1143 | 852.6853 | 1472.122 | 1645.27 | 1450.08 | 1499.132 | 1490.75 | 0.438876 | 2.085253 | 1.56515 |
| Methionine | 1.142 | 148.0429 | 149.05012 | 149.051 | 231.4761 | 146.8799 | 313.3728 | 166.0997 | 259.5342 | 527.5355 | 470.6684 | 224.443 | 494.4098 | 603.1693 | 1.054168 | 2.026132 | 1.55158 |
| L-Proline | 0.855 | 114.0548 | 115.06247 | 115.0633 | 385.1715 | 313.4774 | 272.6467 | 199.3841 | 171.1222 | 341.0673 | 560.8649 | 595.3231 | 433.3656 | 422.6366 | 0.810488 | 1.979631 | 1.53421 |
| Capryloylglycine | 5.41 | 200.1288 | 201.13605 | 201.1365 | 53.16251 | 50.977 | 42.02359 | 53.43812 | 47.65481 | 63.11325 | 100.0241 | 67.35064 | 59.15221 | 93.83109 | 0.633113 | 1.858377 | 1.49876 |
| Coprine | 1.362 | 201.0877 | 202.09495 | 202.0954 | 11.20072 | 12.72547 | 36.44581 | 13.10035 | 23.15651 | 57.29442 | 80.52261 | 26.70366 | 38.70942 | 83.59319 | 1.569636 | 1.84836 | 1.49595 |

| | | | | | | | | | | | | | | | | | |
|---|---|---|---|---|---|---|---|---|---|---|---|---|---|---|---|---|---|
| Uridine diphosphate glucuronic acid | 0.696 | 579.0275 | 580.03462 | 580.0343 | 1059.925 | 748.0373 | 623.8997 | 747.4543 | 530.3155 | 970.4571 | 1427.571 | 899.2302 | 1164.862 | 1636.342 | 0.71717 | 1.698309 | 1.44544 |
| dGDP | 0.734 | 426.0226 | 427.0299 | 427.0294 | 382.0457 | 506.0882 | 908.5407 | 592.4425 | 862.4378 | 1019.545 | 935.875 | 852.0488 | 1043.446 | 944.226 | 0.560443 | 1.68394 | 1.43634 |
| Flavin Mononucleotide | 3.112 | 455.0978 | 456.10502 | 456.1046 | 1101.736 | 1579.814 | 1042.492 | 1398.94 | 990.6596 | 985.9459 | 835.9423 | 841.4571 | 759.8964 | 969.3883 | -0.47695 | 1.652269 | 1.42379 |
| Aminoadipic acid | 0.813 | 160.0608 | 161.06806 | 161.0688 | 400.3646 | 299.6556 | 236.7226 | 164.5646 | 137.7516 | 354.5495 | 485.3113 | 591.913 | 371.5044 | 367.2687 | 0.808814 | 1.647066 | 1.42169 |
| Glutamine | 0.779 | 145.061 | 146.06824 | 146.0691 | 179.7905 | 115.9395 | 212.9192 | 89.12941 | 124.7288 | 308.7189 | 223.7335 | 218.5995 | 444.6793 | 220.8744 | 0.971354 | 1.646882 | 1.4223 |
| Inosine | 1.166 | 267.0737 | 268.08099 | 268.0808 | 144.6882 | 122.5603 | 34.65981 | 167.1089 | 40.71307 | 27.27234 | 19.84342 | 33.77013 | 30.41428 | 16.38234 | -1.99717 | 1.628799 | 1.41707 |
| alpha-D-Glucose 1,6-bisphosphate | 0.701 | 338.989 | 339.99618 | 339.996 | 747.6077 | 683.0457 | 1357.937 | 827.4101 | 1090.648 | 1292.98 | 1240.863 | 1220.45 | 1337.253 | 1402.421 | 0.4644 | 1.609594 | 1.40944 |
| 5-Thymidylic acid | 1.146 | 321.0495 | 322.05675 | 322.0566 | 86.71654 | 158.1284 | 480.8394 | 200.7973 | 336.8647 | 482.2227 | 450.8397 | 407.8944 | 403.8566 | 494.6168 | 0.825881 | 1.561605 | 1.38984 |
| Gluconolactone | 0.814 | 177.04 | 178.04742 | 178.0477 | 877.2686 | 1191.802 | 895.4002 | 1387.35 | 586.544 | 687.1747 | 516.8411 | 594.4617 | 660.9746 | 574.7697 | -0.70271 | 1.558846 | 1.38965 |
| Phenylacetaldehyde | 4.426 | 119.0492 | 120.05668 | 120.0575 | 880.3205 | 1023.663 | 920.4332 | 1368.668 | 1602.192 | 792.9281 | 703.9685 | 756.1749 | 789.1186 | 855.558 | -0.57224 | 1.543885 | 1.38181 |
| Phenyllactic acid | 4.419 | 165.0548 | 166.06215 | 166.063 | 902.8046 | 1052.292 | 946.0741 | 1409.264 | 1643.094 | 816.1045 | 271.8135 | 777.3105 | 813.8008 | 880.0787 | -0.74223 | 1.522056 | 1.37134 |
| Xanthosine | 1.398 | 283.0687 | 284.07602 | 284.0757 | 503.1007 | 575.0841 | 195.4116 | 708.1138 | 141.9764 | 89.46536 | 76.83078 | 275.1327 | 66.24295 | 82.68545 | -1.84691 | 1.517731 | 1.37276 |
| 2-Hydroxymyristic acid | 10.3 | 243.1965 | 244.20386 | 244.2038 | 598.6032 | 656.0965 | 410.8542 | 737.0901 | 515.3527 | 470.6184 | 403.0966 | 487.0312 | 353.6151 | 421.5785 | -0.45011 | 1.468768 | 1.35182 |
| dGMP | 0.804 | 346.056 | 347.06346 | 347.0631 | 328.8684 | 494.944 | 979.02 | 611.3872 | 639.3141 | 968.0166 | 991.6946 | 817.2769 | 817.4544 | 897.9028 | 0.556989 | 1.461921 | 1.34642 |
| Adenine | 1.076 | 134.0462 | 135.05349 | 135.0532 | 1026.37 | 1041.285 | 211.2836 | 982.6962 | 255.3498 | 224.9983 | 227.8996 | 306.3284 | 163.9944 | 161.9725 | -1.69639 | 1.43644 | 1.33727 |
| L-Acetylcarnitine | 4.294 | 202.1081 | 203.11536 | 203.1158 | 85.40475 | 74.27283 | 51.57918 | 77.63925 | 88.72768 | 94.3244 | 196.9312 | 95.5353 | 104.3136 | 197.4226 | 0.866564 | 1.410494 | 1.3281 |
| Uracil | 0.934 | 111.0189 | 112.02618 | 112.0273 | 622.4792 | 564.3163 | 586.9764 | 565.5009 | 574.7136 | 630.378 | 561.4816 | 699.9972 | 786.8926 | 760.4264 | 0.239069 | 1.397876 | 1.32419 |
| Heptadecanoic acid | 13.313 | 269.249 | 270.25618 | 270.2559 | 64.5846 | 78.21475 | 63.20989 | 76.46495 | 70.48125 | 115.8459 | 81.56146 | 106.6091 | 70.41379 | 86.83911 | 0.386124 | 1.397099 | 1.31757 |
| NADP | 0.723 | 742.0692 | 743.07643 | 743.0755 | 832.284 | 1113.928 | 1008.058 | 1381.246 | 1244.144 | 896.2781 | 797.177 | 936.8013 | 883.2516 | 907.2443 | -0.33589 | 1.350093 | 1.29781 |
| Propionylcarnitine | 3.973 | 216.1238 | 217.13109 | 218.1387 | 0.569843 | 0.607267 | 0.576317 | 0.537529 | 0.764899 | 0.864244 | 1.170193 | 0.531935 | 0.907721 | 0.877027 | 0.509811 | 1.347958 | 1.29601 |
| Melibiose | 0.786 | 341.1097 | 342.11685 | 342.1162 | 135.243 | 181.731 | 244.7504 | 239.8644 | 253.0957 | 200.5856 | 71.36508 | 150.1053 | 132.8595 | 138.0918 | -0.60587 | 1.334275 | 1.29018 |
| L-Tryptophan | 2.905 | 203.0819 | 204.0892 | 204.0899 | 67.67556 | 129.9087 | 170.084 | 425.3426 | 672.8157 | 1068.98 | 232.2712 | 913.5391 | 722.4169 | 651.6241 | 1.2918 | 1.327067 | 1.28619 |

| | | | | | | | | | | | | | | | | |
|---|---|---|---|---|---|---|---|---|---|---|---|---|---|---|---|---|
| Glutaric acid | 1.674 | 131.0339 | 132.0412 | 132.0423 | 486.3896 | 356.9089 | 205.8353 | 112.7528 | 182.2296 | 617.8394 | 763.6909 | 263.3953 | 1.27585 | 1.27585 | 1.27585 | 1.27585 | 1.27585 |
| Uridine | 1.139 | 243.0623 | 244.06959 | 244.0695 | 244.1919 | 248.7784 | 259.3312 | 277.2502 | 319.4004 | 253.6863 | 199.7651 | 223.3012 | 224.5108 | 252.9223 | -0.22496 | 1.289083 | 1.26551 |
| L-Asparagine | 0.816 | 309.1027 | 132.05223 | 132.0535 | 217.6659 | 159.3016 | 107.7577 | 115.4569 | 73.8825 | 163.6803 | 260.792 | 172.6344 | 201.3407 | 222.2947 | 0.59866 | 1.2849 | 1.26683 |
| 3-hydroxypentadecanoic acid | 10.384 | 257.2123 | 258.21967 | 258.2195 | 34.88195 | 112.3635 | 112.7445 | 136.4134 | 161.9587 | 218.0062 | 159.9371 | 176.1399 | 128.1114 | 160.1091 | 0.59314 | 1.229342 | 1.23615 |
| L-Lysine | 0.917 | 145.0973 | 146.1046 | 146.1055 | 444.334 | 582.2523 | 636.4154 | 695.9841 | 679.5402 | 599.339 | 318.7786 | 519.6813 | 463.5239 | 418.6813 | -0.38924 | 1.22622 | 1.23749 |
| D-Sedoheptulose 7-phosphate | 0.724 | 289.0334 | 290.04056 | 290.0403 | 521.8634 | 647.7501 | 1557.483 | 811.8564 | 1421.864 | 1675.189 | 1496.81 | 1326.732 | 1534.246 | 1323.471 | 0.568432 | 1.216117 | 1.22923 |
| Glutamylleucine | 1.585 | 259.1301 | 260.13737 | 260.1372 | 79.04504 | 62.91436 | 78.33599 | 62.63105 | 134.2455 | 102.446 | 107.1786 | 120.3201 | 130.4903 | 204.2184 | 0.67196 | 1.214922 | 1.23659 |
| Pantethine | 8.329 | 553.2321 | 554.23954 | 554.2444 | 51.56892 | 51.42243 | 7.580344 | 51.5459 | 7.577728 | 10.51523 | 10.77028 | 16.58202 | 6.081271 | 7.222399 | -1.72954 | 1.209948 | 1.22833 |
| 17alpha-Estradiol | 4.597 | 271.1667 | 272.17396 | 272.1776 | 28.9354 | 27.77482 | 28.289 | 24.1748 | 19.35464 | 24.91363 | 54.75734 | 34.06876 | 29.60052 | 54.28809 | 0.6207 | 1.170119 | 1.20916 |
| 2'-O-Methyluridine | 1.549 | 257.0781 | 258.0855 | 258.0852 | 91.90873 | 186.8835 | 191.0456 | 169.0423 | 66.06045 | 160.3618 | 321.3812 | 197.6008 | 224.0504 | 503.0356 | 0.996464 | 1.162842 | 1.20755 |
| 5-Hydroxy-L-tryptophan | 1.545 | 219.0773 | 220.08454 | 223.0845 | 11.35316 | 11.75931 | 22.34027 | 33.25396 | 72.6032 | 77.35655 | 36.71824 | 71.35265 | 39.88893 | 77.57075 | 1.001274 | 1.148102 | 1.1962 |
| Citric acid | 14.542 | 191.0191 | 192.0264 | 192.027 | 157.8019 | 140.6386 | 166.2573 | 175.0584 | 215.3693 | 214.5416 | 297.5002 | 266.2791 | 175.2995 | 181.1544 | 0.408198 | 1.143884 | 1.18709 |
| Riboflavin | 3.66 | 375.1314 | 376.13887 | 376.1383 | 298.1546 | 148.3452 | 74.39069 | 133.0566 | 104.1265 | 96.39825 | 48.89326 | 86.4118 | 55.33287 | 57.54703 | -1.13749 | 1.143427 | 1.19159 |
| Ribose 1-phosphate | 0.727 | 229.0118 | 230.01939 | 230.0192 | 847.9236 | 802.9491 | 1612.019 | 960.8471 | 1484.434 | 1722.555 | 1533.983 | 1370.033 | 1591.5 | 1374.284 | 0.411518 | 1.141647 | 1.18891 |
| Malic acid | 0.817 | 133.0133 | 134.02053 | 134.0215 | 572.0158 | 587.8553 | 890.5641 | 745.9702 | 1106.288 | 1338.167 | 908.0598 | 1406.861 | 878.7883 | 932.3492 | 0.485547 | 1.127263 | 1.18044 |
| Pentadecanoic acid | 12.686 | 241.2172 | 242.22453 | 242.2246 | 92.36867 | 57.57617 | 8.169585 | 33.40497 | 8.156487 | 7.774762 | 8.11229 | 9.102428 | 6.020649 | 6.688649 | -2.40507 | 1.109701 | 1.17204 |
| Trimetaphosphoric acid | 14.58 | 238.8915 | 239.89872 | 239.899 | 42.79597 | 33.74098 | 29.13752 | 32.2457 | 35.54238 | 33.44842 | 26.41175 | 26.42348 | 32.44465 | 26.83293 | -0.253 | 1.102552 | 1.16865 |
| Theophylline | 0.784 | 179.0556 | 180.06292 | 180.0634 | 1148.721 | 957.0917 | 752.1031 | 1130.075 | 936.7573 | 1183.168 | 2620.572 | 1306.496 | 1088.841 | 3380.661 | 0.959936 | 1.093218 | 1.16776 |
| 3-Phosphoglyceric acid | 0.734 | 184.9849 | 185.99223 | 185.9929 | 956.1943 | 1116.401 | 358.1603 | 1438.964 | 408.6694 | 410.8376 | 451.1621 | 480.0403 | 381.1341 | 508.6571 | -0.93884 | 1.064241 | 1.14687 |
| palmitoleic acid | 12.3 | 253.2174 | 254.22471 | 254.2246 | 178.7737 | 123.244 | 51.23724 | 89.12706 | 38.70742 | 59.19439 | 47.18569 | 37.57899 | 40.19595 | 48.13819 | -1.05036 | 1.051201 | 1.13796 |
| N-Acetyl-glutamine | 1.142 | 187.0718 | 188.07935 | 188.0797 | 16.54851 | 16.52019 | 9.926496 | 12.97506 | 14.4559 | 18.20062 | 19.84284 | 13.51199 | 16.73664 | 27.94333 | 0.450457 | 1.029986 | 1.13211 |
| Citrulline | 0.781 | 174.0877 | 175.095 | 175.0957 | 71.04613 | 84.54627 | 130.6792 | 94.63715 | 86.60261 | 124.3937 | 84.76394 | 155.8307 | 135.9915 | 113.8637 | 0.395218 | 1.027129 | 1.12354 |
| Cytidine | 8.7 | 242.0824 | 243.08957 | 243.0855 | 57.60741 | 45.78758 | 50.10316 | 49.437 | 52.52907 | 49.22079 | 42.00826 | 45.95261 | 49.66129 | 47.2265 | -0.12618 | 0.9538 | 1.07669 |

| | | | | | | | | | | | | | | | | | |
|---|---|---|---|---|---|---|---|---|---|---|---|---|---|---|---|---|---|
| 16-Hydroxyhexadecanoic acid | 11.47 | 271.2281 | 272.23545 | 272.2351 | 52.22452 | 79.10151 | 86.24831 | 111.1736 | 161.393 | 227.8666 | 131.4151 | 137.7853 | 91.05796 | 158.1677 | 0.606545 | 0.936177 | 1.06612 |
| Cinnamic acid | 0.778 | 147.0483 | 148.05559 | 148.0524 | 198.557 | 155.3272 | 241.4237 | 200.9415 | 212.1445 | 254.5356 | 205.6035 | 215.7797 | 253.9219 | 384.9252 | 0.382747 | 0.930285 | 1.06906 |
| 3-Sulfodeoxycholic acid | 13.61 | 457.2269 | 458.23421 | 458.2338 | 3.156594 | 1.326329 | 0.713893 | 1.271632 | 1.094494 | 2.13458 | 2.19144 | 2.272754 | 2.060583 | 4.713572 | 0.822296 | 0.926521 | 1.06621 |
| 3-Hydroxyoctanoic acid | 5.657 | 159.1019 | 160.10915 | 160.1099 | 12.16745 | 13.20812 | 7.675494 | 16.08172 | 12.55718 | 22.94684 | 11.30802 | 16.1727 | 16.77704 | 14.5253 | 0.405828 | 0.905012 | 1.04445 |
| Oleic acid | 13.214 | 281.2487 | 282.2561 | 282.2559 | 773.0125 | 359.7087 | 151.657 | 340.5979 | 199.5409 | 184.1595 | 134.8217 | 158.0068 | 196.9683 | 227.8047 | -1.0167 | 0.87271 | 1.02209 |
| 10Z-Heptadecenoic acid | 12.783 | 267.2333 | 268.2405 | 268.2402 | 56.66939 | 29.42555 | 4.710728 | 18.3372 | 11.58973 | 5.082803 | 10.46161 | 13.74741 | 9.395379 | 5.159415 | -1.46128 | 0.870223 | 1.02162 |
| Uridine 5'-monophosphate | 0.777 | 323.0289 | 324.03626 | 324.0359 | 332.6849 | 354.2616 | 873.0291 | 418.8591 | 453.2314 | 577.7516 | 678.2654 | 643.8799 | 761.127 | 623.3511 | 0.433436 | 0.857388 | 1.0117 |
| 2-Keto-glutaramic acid | 0.814 | 144.0293 | 145.03684 | 145.0375 | 17.35292 | 19.51481 | 27.10099 | 24.45322 | 28.21807 | 27.10761 | 32.03488 | 33.77388 | 21.34566 | 26.15828 | 0.267689 | 0.798184 | 0.966671 |
| Phosphate | 0.76 | 96.96836 | 97.97569 | 97.9769 | 11167.82 | 8960.673 | 7577.484 | 9115.028 | 7947.055 | 7896.313 | 7071.575 | 7139.858 | 7849.234 | 9207.218 | -0.19293 | 0.781128 | 0.952923 |
| Adenosine | 1.261 | 266.0892 | 267.0971 | 267.0968 | 2310.062 | 543.009 | 29.66381 | 706.4325 | 45.68825 | 33.20716 | 23.28462 | 334.5526 | 25.48118 | 29.90832 | -3.02538 | 0.770978 | 0.949518 |
| N-acetyl-D-methionine | 3.142 | 190.0537 | 191.061 | 191.0616 | 250.2753 | 68.34137 | 53.00909 | 132.6711 | 77.25002 | 70.88403 | 57.30957 | 81.0789 | 45.33879 | 50.38198 | -0.93112 | 0.769323 | 0.949239 |
| myristic acid | 12.034 | 227.2014 | 228.20872 | 228.2089 | 4.533686 | 16.09414 | 20.50443 | 24.61721 | 29.06156 | 52.91422 | 28.03713 | 17.45674 | 20.74217 | 31.15208 | 0.664741 | 0.751102 | 0.933174 |
| 3-Oxohexanoic acid | 3.344 | 129.0546 | 130.06192 | 130.063 | 299.8839 | 114.5144 | 51.41562 | 101.907 | 47.43627 | 41.31146 | 53.36174 | 89.92705 | 35.29384 | 55.78098 | -1.15799 | 0.726497 | 0.914382 |
| Genistein | 6.096 | 269.0459 | 270.05319 | 270.0528 | 90.12943 | 75.01381 | 84.40372 | 78.58013 | 93.09457 | 84.54055 | 96.40278 | 95.67387 | 84.80385 | 90.07461 | 0.100132 | 0.718311 | 0.907512 |
| Erythrose 4-phosphate | 0.698 | 199.0007 | 200.00853 | 200.0086 | 50.80035 | 31.22943 | 20.28619 | 28.51553 | 21.28529 | 29.81394 | 40.51351 | 41.61937 | 38.57977 | 42.80522 | 0.3459 | 0.688511 | 0.885882 |
| Purine | 1.135 | 119.0339 | 120.04114 | 120.0436 | 26.17159 | 32.20865 | 32.94059 | 39.05539 | 58.14037 | 31.67552 | 27.8502 | 29.90591 | 28.2535 | 32.56704 | -0.32731 | 0.683286 | 0.878746 |
| Glucose 6-phosphate | 0.734 | 259.0225 | 260.02975 | 260.0297 | 340.7788 | 368.9855 | 836.7121 | 492.0557 | 585.7759 | 713.5085 | 542.2031 | 826.3168 | 614.3574 | 618.4696 | 0.337009 | 0.671537 | 0.868374 |
| Guanosine | 1.175 | 282.0846 | 283.09183 | 283.0917 | 100.5497 | 34.4541 | 8.607868 | 30.75297 | 6.678093 | 14.8726 | 12.8591 | 20.03937 | 10.46046 | 8.750918 | -1.43448 | 0.657246 | 0.85798 |
| Pantothenic acid | 2.343 | 218.1031 | 219.11036 | 219.1107 | 574.5191 | 419.6395 | 438.8561 | 378.1301 | 453.435 | 465.4811 | 964.448 | 407.2869 | 387.6582 | 1626.409 | 0.766096 | 0.648379 | 0.856619 |
| 3-Hydroxynonanoic acid | 6.522 | 173.1177 | 174.12492 | 174.1256 | 18.57668 | 15.73789 | 11.49582 | 11.75864 | 21.46452 | 19.40919 | 9.486762 | 11.57074 | 11.62278 | 11.54274 | -0.31271 | 0.572231 | 0.781517 |
| 12-Hydroxystearic acid | 13.113 | 299.2596 | 300.26672 | 300.2664 | 718.485 | 31.016 | 153.5344 | 322.7298 | 203.3707 | 186.9804 | 138.4777 | 159.9142 | 194.8117 | 34.47744 | -0.99981 | 0.56416 | 0.777158 |
| N-Lauroylglycine | 9.248 | 256.1921 | 257.19936 | 257.1991 | 75.90794 | 52.69977 | 89.40849 | 52.37721 | 65.80961 | 57.5046 | 60.84157 | 70.09595 | 50.95335 | 50.92425 | -0.21169 | 0.552626 | 0.766241 |

| | | | | | | | | | | | | | | | | | |
|---|---|---|---|---|---|---|---|---|---|---|---|---|---|---|---|---|---|
| FAD | 2.86 | 784.1506 | 785.1581 | 785.1571 | 668.4966 | 670.322 | 1144.35 | 903.4167 | 1197.571 | 1017.612 | 1110.11 | 1106.776 | 859.3852 | 1201.168 | 0.207989 | 0.532393 | 0.745313 |
| Fumaric acid | 1.152 | 291.035 | 116.01059 | 116.011 | 10.35414 | 17.40809 | 13.51828 | 13.24548 | 14.80841 | 15.92403 | 9.727008 | 8.069738 | 9.105192 | 15.71436 | -0.24414 | 0.490758 | 0.698813 |
| Indoleacetic acid | 4.478 | 174.0553 | 175.06266 | 175.0633 | 97.82738 | 110.7564 | 122.0676 | 148.4098 | 173.9685 | 148.2432 | 77.10418 | 107.9758 | 97.5535 | 127.194 | -0.2267 | 0.48066 | 0.691763 |
| Glycerol 3-phosphate | 0.74 | 171.0055 | 172.01286 | 172.0137 | 33196.81 | 34931.18 | 41517.47 | 31063.6 | 28833.78 | 29912.39 | 39155.55 | 24966.15 | 29539.45 | 29580.83 | -0.14666 | 0.479829 | 0.694258 |
| Panthenol | 2.381 | 204.1237 | 205.13099 | 205.1314 | 0.617647 | 0.516502 | 0.546345 | 0.521248 | 0.616077 | 0.767928 | 46.1796 | 0.520395 | 0.514282 | 0.634986 | 4.108816 | 0.463041 | 0.670774 |
| Testosterone sulfate | 5.72 | 367.1587 | 368.16603 | 368.1657 | 0.284074 | 0.223551 | 0.238001 | 0.23136 | 0.269892 | 0.224077 | 462.42 | 0.199017 | 0.213832 | 0.214794 | 8.537394 | 0.459955 | 0.667543 |
| lauric acid | 7.62 | 199.1691 | 200.17649 | 200.1776 | 87.21006 | 85.38941 | 90.82345 | 84.6492 | 93.90352 | 88.63917 | 86.64409 | 99.62049 | 95.38709 | 87.10296 | 0.04947 | 0.456205 | 0.666606 |
| Guanosine monophosphate | 1.137 | 362.0513 | 363.05827 | 363.058 | 65.40188 | 48.61388 | 33.31674 | 55.77982 | 23.33181 | 34.35719 | 41.2916 | 25.43452 | 37.50152 | 47.27786 | -0.28492 | 0.440094 | 0.648943 |
| Indole | 3.153 | 116.0495 | 117.05698 | 117.0578 | 348.8313 | 392.9561 | 396.1535 | 313.8369 | 331.5401 | 349.1931 | 299.776 | 313.7626 | 406.9631 | 283.2052 | -0.10956 | 0.432186 | 0.643126 |
| N-Acetyl-D-valine | 3.118 | 158.0813 | 159.08865 | 159.0895 | 365.5723 | 63.77245 | 63.57558 | 48.65424 | 56.11237 | 179.1207 | 214.8703 | 168.6093 | 147.8646 | 183.2684 | 0.580455 | 0.430131 | 0.640204 |
| Capric acid | 14.596 | 171.1384 | 172.14562 | 172.1463 | 16.7124 | 10.03596 | 31.23708 | 22.10586 | 19.44158 | 11.04465 | 36.35351 | 26.26765 | 20.29471 | 31.6908 | 0.336181 | 0.419812 | 0.628774 |
| Uridine diphosphate-N-acetylglucosamine | 0.691 | 606.075 | 607.08209 | 607.0816 | 2656.555 | 1959.828 | 2216.16 | 1529.67 | 1175.49 | 1474.367 | 3124.733 | 1556.708 | 1874.047 | 3992.46 | 0.334001 | 0.398219 | 0.610069 |
| Methylmalonic acid | 1.244 | 117.0181 | 118.02542 | 118.0266 | 44828.26 | 32270.52 | 34397.98 | 30629.87 | 32169.42 | 38585.38 | 43434.79 | 30725.24 | 33008.93 | 45044.58 | 0.130513 | 0.386954 | 0.595 |
| Monoethylhexyl phthalic acid | 9.592 | 277.1447 | 278.15204 | 278.1518 | 46.68861 | 72.35213 | 21.98299 | 17.38238 | 13.96796 | 23.21035 | 45.12816 | 22.08624 | 15.69065 | 12.37936 | -0.54072 | 0.384634 | 0.590077 |
| Oxidized glutathione | 1.145 | 611.1451 | 612.15249 | 612.152 | 500.1326 | 187.9321 | 164.1432 | 92.99431 | 56.26245 | 172.0867 | 542.5975 | 144.6575 | 177.4913 | 460.7484 | 0.580523 | 0.384284 | 0.591097 |
| Dethiobiotin | 1.807 | 213.1242 | 214.13148 | 214.1317 | 19.62989 | 21.58977 | 22.74328 | 46.00973 | 99.6459 | 65.49942 | 35.74807 | 83.40922 | 58.09469 | 41.94417 | 0.441654 | 0.382943 | 0.586033 |
| Porphobilinogen | 2.147 | 225.0879 | 226.09517 | 226.0954 | 176.8608 | 454.5608 | 219.6103 | 523.3612 | 756.0271 | 354.8114 | 319.7422 | 198.9237 | 167.5324 | 565.3144 | -0.40738 | 0.363014 | 0.560496 |
| Luteolin | 5.55 | 285.0408 | 286.04807 | 286.0477 | 31.68339 | 16.78575 | 44.3238 | 5.560437 | 37.71965 | 23.3685 | 34.66935 | 21.9475 | 51.38421 | 40.87499 | 0.340077 | 0.353507 | 0.55621 |
| Palmitic acid | 13.153 | 255.233 | 256.24032 | 256.2402 | 1386.593 | 565.5029 | 512.8017 | 568.2825 | 709.6141 | 774.0405 | 444.1958 | 460.3515 | 627.5776 | 728.6314 | -0.30252 | 0.351668 | 0.548304 |
| Azelaic acid | 4.979 | 187.097 | 188.1043 | 188.1049 | 403.1203 | 951.3894 | 168.1843 | 600.201 | 1576.48 | 1218.292 | 210.5069 | 215.7471 | 535.0808 | 277.901 | -0.59007 | 0.349059 | 0.547679 |
| L-Arginine | 0.871 | 173.1036 | 174.11086 | 174.1117 | 184.8255 | 131.7671 | 238.7973 | 324.4369 | 418.1858 | 137.6323 | 185.8951 | 175.2566 | 290.2582 | 274.4308 | -0.28752 | 0.348217 | 0.544783 |
| N-Acetylhistidine | 0.818 | 196.0723 | 197.08007 | 197.08 | 77.28232 | 93.95666 | 87.06512 | 77.25466 | 87.60247 | 72.67325 | 85.54219 | 79.53886 | 90.36383 | 77.172 | -0.06225 | 0.347063 | 0.544738 |

| | | | | | | | | | | | | | | | | | | |
|---|---|---|---|---|---|---|---|---|---|---|---|---|---|---|---|---|---|---|
| 2-Ethylhexanoic acid | 14.615 | 143.1068 | 144.1141 | 144.115 | 9.591324 | 13.93891 | 31.46418 | 21.07582 | 26.44663 | 7.374789 | 39.4281 | 25.72658 | 22.05257 | 34.37307 | 0.331008 | 0.336711 | 0.53389 |
| Diflunisal | 4.595 | 249.0323 | 250.03961 | 250.0442 | 294.4382 | 23.61842 | 9.48501 | 132.1 | 200.5144 | 244.335 | 1.006672 | 196.7156 | 266.9829 | 225.542 | 0.501514 | 0.329947 | 0.527148 |
| Mevalonic acid-5P | 1.141 | 227.0325 | 228.03973 | 228.0399 | 103.6425 | 75.52383 | 80.39256 | 113.503 | 111.4389 | 127.9972 | 99.14537 | 71.70626 | 108.5962 | 126.747 | 0.14086 | 0.329794 | 0.5266 |
| Linoleic acid | 12.535 | 279.2333 | 280.24045 | 280.2402 | 68.49021 | 20.619 | 34.27868 | 66.50542 | 44.3705 | 53.24414 | 39.67509 | 36.71425 | 61.72733 | 96.26256 | 0.296047 | 0.326199 | 0.525002 |
| 3-Hydroxybutyric acid | 1.556 | 103.0389 | 104.04615 | 104.0473 | 290.7775 | 652.5427 | 474.3057 | 2056.71 | 2015.666 | 966.0641 | 573.9622 | 845.8846 | 687.388 | 930.6427 | -0.45539 | 0.325434 | 0.519682 |
| 5'-Methylthioadenosine | 3.149 | 296.0825 | 297.08988 | 297.0896 | 2466.39 | 2210.728 | 3537.091 | 2439.803 | 3454.812 | 3423.637 | 3243.621 | 2252.946 | 2847.21 | 3781.746 | 0.140239 | 0.324981 | 0.521406 |
| L-Theanine | 1.576 | 173.0925 | 174.09974 | 174.1004 | 54.47202 | 46.69146 | 25.88399 | 54.20813 | 69.83256 | 46.62648 | 45.30388 | 59.45109 | 54.77803 | 81.09899 | 0.194155 | 0.323789 | 0.523105 |
| 2-Isopropylmalic acid | 3.324 | 175.0605 | 176.06779 | 176.0685 | 28.26324 | 33.79426 | 36.39244 | 32.4978 | 24.96306 | 31.9772 | 38.53382 | 31.9409 | 26.01384 | 38.87452 | 0.102064 | 0.311453 | 0.503042 |
| Pyroglutamic acid | 1.144 | 128.0342 | 129.04154 | 129.0426 | 1200.628 | 1265.336 | 816.0588 | 1211.865 | 1892.102 | 1374.05 | 819.8643 | 1437.329 | 1010.133 | 993.6777 | -0.18048 | 0.305093 | 0.493599 |
| 3-Oxohexadecanoic acid | 9.967 | 269.2126 | 270.21985 | 270.2195 | 68.61073 | 50.81077 | 17.74596 | 47.4578 | 43.0742 | 47.85195 | 41.56099 | 86.57391 | 39.37306 | 53.07417 | 0.237437 | 0.291299 | 0.476545 |
| L-Lactic acid | 1.14 | 89.02315 | 90.03041 | 90.03169 | 167.9417 | 690.4478 | 185.4438 | 623.0549 | 901.5439 | 763.6836 | 411.9066 | 570.8181 | 629.7753 | 724.5164 | 0.271706 | 0.284784 | 0.470092 |
| N-Phenylacetylglutamic acid | 4.388 | 264.0881 | 265.09527 | 265.095 | 291.6624 | 572.4587 | 446.8634 | 623.2153 | 518.7216 | 424.7354 | 321.5067 | 479.5687 | 471.1915 | 542.676 | -0.13121 | 0.259338 | 0.430624 |
| Caffeic acid | 3.599 | 179.0343 | 180.04159 | 180.0423 | 25.10146 | 8.786196 | 28.38329 | 2.474825 | 33.70745 | 8.194245 | 8.783711 | 5.601103 | 38.34546 | 11.70075 | -0.43897 | 0.25167 | 0.421842 |
| 1,2-Benzisothiazol-3(2H)-one | 5.389 | 150.001 | 151.00829 | 151.0092 | 289.5911 | 233.75 | 228.7148 | 232.9094 | 317.7092 | 338.893 | 246.9367 | 226.6018 | 183.956 | 209.5748 | -0.11129 | 0.248657 | 0.421913 |
| L-Phenylalanine | 2.199 | 164.071 | 165.07815 | 165.079 | 2189.307 | 2773.69 | 6888.392 | 4958.879 | 4335.329 | 3797.627 | 3725.293 | 3438.863 | 8319.048 | 5482.664 | 0.227858 | 0.240384 | 0.409193 |
| 12-Hydroxydodecanoic acid | 8.847 | 215.165 | 216.17225 | 216.1725 | 21.45502 | 16.31361 | 11.51762 | 19.04961 | 19.18096 | 19.63495 | 12.86125 | 20.36426 | 11.22025 | 16.29186 | -0.12286 | 0.236223 | 0.402313 |
| Docosahexaenoic acid | 8.964 | 327.2293 | 328.23661 | 328.2402 | 195.972 | 204.7903 | 220.6003 | 225.8777 | 339.7145 | 309.1682 | 166.7451 | 248.1543 | 156.8997 | 195.0254 | -0.1416 | 0.236028 | 0.403112 |
| Adipic acid | 1.523 | 145.0497 | 146.05695 | 146.0579 | 221.3645 | 212.9777 | 87.38815 | 430.358 | 114.5423 | 142.6412 | 91.06691 | 437.7376 | 64.68204 | 75.71353 | -0.39379 | 0.224197 | 0.388781 |
| Thymidine | 1.764 | 241.0829 | 242.0904 | 242.0903 | 45.76813 | 26.89812 | 7.074795 | 46.42082 | 10.53553 | 13.87443 | 18.57874 | 147.9017 | 13.02724 | 17.83592 | 0.627747 | 0.217875 | 0.374255 |
| Indolelactic acid | 4.624 | 204.0662 | 205.07352 | 205.0739 | 14.70185 | 33.05775 | 48.26523 | 71.94244 | 131.9556 | 73.90455 | 33.2755 | 39.50485 | 51.95904 | 44.80165 | -0.30099 | 0.212479 | 0.369169 |
| 4-Dodecylbenzenesulfonic Acid | 8.745 | 325.1847 | 326.19195 | 326.1916 | 1143.682 | 659.7785 | 824.124 | 1258.339 | 2008.852 | 2409.711 | 1292.243 | 1385.985 | 671.8617 | 1101.617 | 0.21907 | 0.210772 | 0.363392 |

| | | | | | | | | | | | | | | | | |
|---|---|---|---|---|---|---|---|---|---|---|---|---|---|---|---|---|
| Tyrosine methylester | 8.621 | 194.0818 | 195.08904 | 195.0895 | 56.63788 | 47.74452 | 46.86553 | 43.56286 | 53.69313 | 48.72511 | 43.53899 | 47.58847 | 56.14644 | 44.36157 | -0.04807 | 0.199438 | 0.349047 |
| Tridecanoylglycine | 7.519 | 270.2078 | 271.2151 | 271.2147 | 54.15273 | 9.974036 | 10.82215 | 76.43132 | 299.5542 | 487.3165 | 15.44023 | 9.19716 | 151.0912 | 29.39277 | 0.618767 | 0.180459 | 0.3195 |
| L-2-amino-3-oxobutanoic acid | 0.798 | 233.0779 | 117.04261 | 117.0426 | 35.86545 | 53.39801 | 74.86732 | 76.58315 | 49.84863 | 47.31822 | 51.49645 | 46.02481 | 62.7649 | 118.8047 | 0.167832 | 0.179739 | 0.326816 |
| 3-Hydroxysebacic acid | 4.497 | 217.1079 | 218.11519 | 218.1154 | 7.22433 | 10.54873 | 3.410569 | 12.04 | 24.60916 | 24.03219 | 3.084282 | 3.193227 | 9.282918 | 6.502798 | -0.32726 | 0.17498 | 0.313466 |
| Benzoic acid | 3.507 | 121.0284 | 122.03569 | 122.0368 | 32.53033 | 4.484963 | 11.93733 | 149.0796 | 87.58178 | 10.32785 | 65.52961 | 28.16726 | 75.75465 | 201.163 | 0.415506 | 0.173894 | 0.316274 |
| Tetradecanedioic acid | 8.003 | 257.1762 | 258.18342 | 258.1831 | 11.75464 | 5.028195 | 4.766184 | 7.290795 | 17.13514 | 11.18921 | 6.711821 | 8.755052 | 6.542834 | 7.31237 | -0.18252 | 0.171483 | 0.307824 |
| 2-Hydroxycaproic acid | 4.069 | 131.0703 | 132.07759 | 132.0786 | 2151.376 | 2264.559 | 2044.108 | 3191.768 | 3754.272 | 2425.497 | 2556.841 | 2859.857 | 2316.257 | 2508.726 | -0.08179 | 0.165387 | 0.299413 |
| Sulfate | 14.94 | 96.95888 | 97.96617 | 97.96738 | 10695.33 | 3679.199 | 7659.971 | 2177.125 | 8700.499 | 4538.805 | 5117.279 | 5155.602 | 7521.241 | 7014.908 | -0.16536 | 0.164505 | 0.293565 |
| L-Aspartic acid | 0.777 | 132.0292 | 133.0365 | 133.0375 | 737.9192 | 574.4989 | 858.1694 | 977.2365 | 883.6819 | 962.4853 | 684.3115 | 669.8954 | 1031.753 | 886.9705 | 0.071185 | 0.157153 | 0.28553 |
| 2-hydroxyglutaric acid | 1.138 | 147.0289 | 148.03622 | 148.0372 | 360.5684 | 167.1325 | 123.0327 | 98.54374 | 173.9166 | 196.4997 | 208.4594 | 93.9449 | 151.435 | 171.3285 | -0.16808 | 0.156526 | 0.283377 |
| Allopurinol | 1.139 | 135.0302 | 136.03746 | 136.0385 | 145.5325 | 519.184 | 598.4162 | 747.4004 | 761.4978 | 810.7435 | 383.5962 | 729.078 | 632.1461 | 472.0208 | 0.127224 | 0.143341 | 0.261021 |
| NAD | 1.137 | 662.1027 | 663.11001 | 663.1091 | 402.3656 | 421.106 | 303.8916 | 412.0613 | 291.6624 | 356.079 | 413.8232 | 393.2356 | 278.7087 | 320.4674 | -0.05523 | 0.141701 | 0.264404 |
| N-Undecylbenzenesulfonic acid | 8.27 | 311.1692 | 312.17638 | 312.1759 | 988.7959 | 417.2158 | 658.454 | 1248.566 | 1677.138 | 1881.89 | 1003.76 | 982.9014 | 401.6714 | 1290.935 | 0.156296 | 0.133308 | 0.246698 |
| Deoxycholic Acid | 9.026 | 391.2859 | 392.29319 | 392.2927 | 58.23442 | 5.346998 | 9.150187 | 2.328255 | 135.9694 | 51.79162 | 37.15797 | 4.648434 | 4.402276 | 64.19891 | -0.37968 | 0.131235 | 0.240865 |
| 3-Methyladipic acid | 3.57 | 159.0655 | 160.07279 | 160.0736 | 30.49131 | 15.44884 | 14.07447 | 18.60784 | 31.54415 | 96.15782 | 5.243639 | 9.089198 | 15.53489 | 14.23522 | 0.348425 | 0.130656 | 0.241222 |
| Gallic acid | 14.188 | 169.0134 | 170.0207 | 170.0215 | 75.70239 | 42.32799 | 43.66167 | 17.81966 | 21.61861 | 29.32175 | 34.86396 | 32.36434 | 38.91316 | 48.44601 | -0.12914 | 0.120075 | 0.221478 |
| Xanthine | 1.14 | 151.0252 | 152.03249 | 152.0334 | 1779.526 | 2978.642 | 3190.089 | 3120.876 | 2511.017 | 3309.662 | 2093.337 | 2847.267 | 2413.02 | 2393.201 | -0.05673 | 0.116847 | 0.222108 |
| Dodecanedioic acid | 6.849 | 229.1445 | 230.15171 | 230.1518 | 24.56788 | 16.35058 | 12.65307 | 15.56569 | 40.277 | 27.06627 | 15.09904 | 24.36646 | 17.32159 | 17.23891 | -0.11413 | 0.11317 | 0.213684 |
| N'-Formylkynurenine | 1.406 | 235.0723 | 236.07965 | 236.0797 | 39.79069 | 56.82473 | 40.20245 | 118.6536 | 54.07786 | 71.01015 | 31.69073 | 160.7734 | 30.93626 | 52.01686 | 0.162384 | 0.097032 | 0.183522 |
| Suberic acid | 4.307 | 173.0812 | 174.08853 | 174.0892 | 114.0397 | 118.8992 | 75.62277 | 106.0932 | 199.3445 | 228.1924 | 52.03537 | 65.4744 | 121.8395 | 99.42147 | -0.11498 | 0.092749 | 0.17814 |
| Xanthylic acid | 0.792 | 363.0353 | 364.04273 | 364.042 | 177.282 | 185.3081 | 297.1475 | 303.141 | 197.4082 | 257.0106 | 208.0894 | 229.4654 | 204.3994 | 224.3662 | -0.0467 | 0.092257 | 0.180846 |

| | | | | | | | | | | | | | | | | |
|---|---|---|---|---|---|---|---|---|---|---|---|---|---|---|---|---|
| Daucic acid | 0.962 | 203.0182 | 204.02551 | 204.027 | 15.31159 | 75.75782 | 89.40819 | 110.2958 | 106.2161 | 106.1756 | 57.47774 | 113.0719 | 75.76466 | 68.99281 | 0.086372 | 0.088722 | 0.169043 |
| stearic acid | 14.18 | 283.2643 | 284.27171 | 284.2715 | 1062.904 | 484.296 | 773.4118 | 708.3111 | 1104.953 | 866.9544 | 764.1536 | 715.9733 | 725.4534 | 915.0621 | -0.05198 | 0.08784 | 0.168031 |
| Indole-3-carboxaldehyde | 4.909 | 144.0445 | 145.05182 | 145.0528 | 450.3947 | 460.2759 | 138.2458 | 439.7974 | 305.523 | 457.3586 | 182.066 | 616.3459 | 159.103 | 254.9513 | -0.10367 | 0.084633 | 0.165661 |
| L-Histidine | 0.796 | 154.0614 | 155.06863 | 155.0695 | 1137.961 | 1379.234 | 454.4368 | 1318.794 | 1528.3 | 463.3074 | 1416.472 | 526.7112 | 1796.138 | 2019.774 | 0.096769 | 0.079035 | 0.159835 |
| Traumatic acid | 7.187 | 227.1288 | 228.13599 | 228.1362 | 32.34044 | 24.96376 | 23.46776 | 22.51794 | 31.73615 | 23.56575 | 27.07689 | 26.63748 | 34.95259 | 25.87251 | 0.03253 | 0.078731 | 0.154953 |
| Erucic acid | 13.127 | 337.3117 | 338.31897 | 338.3185 | 0.65682 | 36.71423 | 0.722668 | 0.708814 | 25.31674 | 0.352953 | 3.563297 | 5.507737 | 21.47949 | 25.93946 | -0.17378 | 0.056277 | 0.105584 |
| Tetraacetylethylenediamine | 2.952 | 227.1034 | 228.11085 | 228.111 | 1080.475 | 1420.162 | 1628.797 | 1844.508 | 2424.204 | 1703.438 | 1663.791 | 1707.647 | 1752.706 | 1739.519 | 0.028736 | 0.053304 | 0.106571 |
| L-Glutamic acid | 0.781 | 146.045 | 147.05223 | 147.0532 | 4525.482 | 7426.07 | 7838.609 | 6562.393 | 6460.528 | 9134.825 | 9107.595 | 4686.512 | 5796.112 | 4881.486 | 0.03447 | 0.048844 | 0.093162 |
| 2-Hydroxyvalerate | 2.885 | 117.0546 | 118.06186 | 118.063 | 191.1899 | 170.773 | 198.9977 | 176.6866 | 201.0569 | 233.6699 | 156.8743 | 188.9477 | 144.9319 | 204.7762 | -0.01468 | 0.038787 | 0.076907 |
| N-Nitrosoproline | 0.789 | 143.0454 | 144.05287 | 144.0535 | 17.14223 | 18.27118 | 30.09232 | 36.70595 | 27.48439 | 14.71214 | 22.25421 | 16.96143 | 24.80433 | 54.53832 | 0.039222 | 0.030701 | 0.069401 |
| Nicotinic acid | 1.14 | 122.0235 | 123.0309 | 123.032 | 443.0266 | 340.5947 | 159.1182 | 537.9143 | 93.99836 | 360.1349 | 271.1808 | 295.0134 | 251.4003 | 361.178 | -0.03313 | 0.028599 | 0.059089 |
| Phosphoglycolic acid | 0.734 | 154.9743 | 155.98185 | 155.9824 | 37.03484 | 66.59331 | 64.29855 | 99.60004 | 72.59545 | 68.95882 | 55.52915 | 84.40874 | 49.19749 | 77.9908 | -0.01723 | 0.02323 | 0.048027 |
| Tretinoin | 11.092 | 299.2018 | 300.20916 | 300.2089 | 2393.976 | 2127.395 | 2512.599 | 1529.719 | 2709.411 | 3370.218 | 1938.125 | 1651.455 | 2185.577 | 2026.867 | -0.01297 | 0.019208 | 0.03994 |
| Lycoperdic acid | 0.779 | 216.0507 | 217.05795 | 217.0586 | 52.24181 | 78.51072 | 35.11382 | 57.63307 | 57.8708 | 48.74568 | 34.26087 | 85.46356 | 60.5394 | 54.30688 | 0.009944 | 0.012157 | 0.027357 |
| Deoxyribose 5-monophosphate | 1.117 | 213.0168 | 214.02406 | 214.0242 | 50.15597 | 57.32262 | 58.94266 | 24.44096 | 45.91881 | 59.91471 | 45.49142 | 33.50887 | 50.99367 | 45.70647 | -0.00712 | 0.010511 | 0.020091 |
| N-Undecanoylglycine | 6.423 | 242.1762 | 243.18346 | 243.1834 | 1020.377 | 261.7342 | 280.3141 | 1319.412 | 6154.971 | 5611.05 | 344.8234 | 237.3565 | 2181.218 | 610.4897 | -0.00831 | 0.002326 | 0.006027 |
| Phthalic acid | 3.507 | 165.0185 | 166.02581 | 166.0266 | 220.0121 | 62.68614 | 132.0428 | 1536.869 | 84.27533 | 81.6951 | 31.2861 | 291.8841 | 94.28907 | 1536.294 | -0.00031 | 7.29E-05 | 0.003968 |

**Table S4. List of all identified metabolites (positive data, FC = [RS218]$_{metabolite}$/ [IHE3034]$_{metabolite}$)**

| Compound | RT [min] | m/z | MW | Mono isotopic Mass | IHE_1 | IHE_2 | IHE_3 | IHE_4 | IHE_5 | RS_1 | RS_2 | RS_3 | RS_4 | RS_5 | Log2(FC) | Log10(p-value) | VIP |
|---|---|---|---|---|---|---|---|---|---|---|---|---|---|---|---|---|---|

| | | | | | | | | | | | | | | | | |
|---|---|---|---|---|---|---|---|---|---|---|---|---|---|---|---|---|
| N-acetyl-L-glutamic acid | 1.4 | 190.0717 | 189.0645 | 189.0637 | 11.30718 | 19.57808 | 21.60692 | 22.33496 | 23.66633 | 59.34965 | 37.67783 | 54.75554 | 49.28797 | 53.01878 | 1.367238 | 4.070164 | 1.95205 |
| Dimethylglycine | 0.821 | 104.0714 | 103.0642 | 103.0633 | 44.15614 | 67.76349 | 49.9289 | 79.77956 | 134.3032 | 282.2671 | 186.3967 | 285.5344 | 219.4042 | 245.9783 | 1.697844 | 3.863818 | 1.93377 |
| Piperidine | 0.909 | 86.09734 | 85.08999 | 85.08915 | 542.0876 | 553.8123 | 650.6232 | 581.4073 | 434.8763 | 811.0251 | 1143.975 | 962.4985 | 976.0766 | 968.5864 | 0.815463 | 3.788341 | 1.92637 |
| L-Carnitine | 14.536 | 162.1132 | 161.1059 | 162.113 | 294.8748 | 283.585 | 259.1423 | 249.5061 | 254.4614 | 165.257 | 221.1681 | 164.9389 | 179.0368 | 170.937 | -0.57378 | 3.67766 | 1.91503 |
| O-Toluidine | 14.691 | 108.0817 | 107.0744 | 107.0735 | 94 | 90.23967 | 100.4769 | 92.11906 | 94.28034 | 77.89879 | 82.04826 | 82.90756 | 83.37411 | 82.33689 | -0.20551 | 3.636191 | 1.91076 |
| L-Alanine | 0.804 | 90.05599 | 89.04865 | 89.04768 | 72.05323 | 89.7024 | 107.9136 | 108.9999 | 161.0787 | 548.9608 | 346.7552 | 739.9427 | 423.6869 | 651.9006 | 2.328599 | 3.448245 | 1.88907 |
| Nicotine | 14.664 | 163.1236 | 162.1164 | 162.1157 | 1760.345 | 1880.613 | 1899.829 | 1728.37 | 2039.821 | 1365.949 | 1531.491 | 1496.067 | 1499.206 | 1585.288 | -0.31597 | 3.255949 | 1.86419 |
| alpha-D-Glucose 1,6-bisphosphate | 0.709 | 341.0046 | 339.9971 | 339.996 | 6.269002 | 3.889905 | 5.18663 | 4.677418 | 4.883437 | 6.483404 | 8.359373 | 8.76632 | 6.85311 | 8.634105 | 0.650517 | 2.763988 | 1.78531 |
| N-Acetyl-D-leucine | 3.994 | 174.1132 | 173.106 | 173.1052 | 143.2724 | 112.2037 | 88.92847 | 88.74365 | 74.6586 | 175.9187 | 259.4042 | 182.951 | 161.7209 | 198.5608 | 0.946374 | 2.710269 | 1.77524 |
| Sphinganine | 10.386 | 302.3063 | 301.2991 | 301.2981 | 76.69611 | 123.0651 | 102.0098 | 53.79624 | 29.87416 | 3.191893 | 9.617234 | 3.730432 | 11.85833 | 2.280278 | -3.65123 | 2.551469 | 1.74288 |
| L-Proline | 0.816 | 116.0714 | 115.0642 | 115.0633 | 664.4165 | 785.3083 | 1174.758 | 949.1281 | 1058.072 | 1524.51 | 1739.882 | 2153.122 | 1267.651 | 1591.952 | 0.837592 | 2.54056 | 1.74064 |
| NAD | 1.134 | 664.1176 | 663.1112 | 663.1091 | 145.3532 | 107.1131 | 96.22915 | 123.3631 | 98.10967 | 61.07299 | 84.26792 | 69.99052 | 43.38927 | 73.39363 | -0.77971 | 2.496932 | 1.73102 |
| Alverine | 12.68 | 282.2228 | 281.2153 | 281.2143 | 19.90292 | 18.65477 | 14.9041 | 19.71598 | 16.76119 | 14.01536 | 14.62247 | 11.75692 | 12.38105 | 14.29147 | -0.42334 | 2.482554 | 1.72782 |
| Stearoylethanolamide | 12.987 | 328.3225 | 327.315 | 327.3137 | 8.255168 | 5.871148 | 8.224011 | 8.32151 | 9.124302 | 4.796106 | 5.291321 | 5.129846 | 4.871898 | 6.63573 | -0.57444 | 2.446423 | 1.71984 |
| L-Valine | 0.803 | 118.087 | 117.0797 | 117.079 | 9660.771 | 12532.93 | 20965.94 | 12980.08 | 13017.01 | 28167.44 | 20035.59 | 41974.95 | 30056.03 | 26981.31 | 1.089986 | 2.325879 | 1.69101 |
| Methylisocitric acid | 1.203 | 207.051 | 206.0436 | 206.0427 | 316.1265 | 432.2871 | 549.7955 | 533.1284 | 404.003 | 342.6965 | 279.5583 | 289.2456 | 243.8068 | 231.7498 | -0.68847 | 2.146336 | 1.64393 |
| Flavin Mononucleotide | 3.098 | 457.1133 | 456.106 | 456.1046 | 41.76701 | 58.71142 | 39.62762 | 62.57742 | 31.70145 | 26.59729 | 25.00743 | 25.37196 | 20.46933 | 29.98515 | -0.87916 | 2.099583 | 1.63063 |
| Mevalonic acid-5P | 7.151 | 229.0438 | 228.0366 | 228.0399 | 10.41498 | 12.64099 | 18.74408 | 21.17047 | 16.98635 | 10.55798 | 10.1456 | 7.941481 | 5.257061 | 7.693492 | -0.94279 | 2.094184 | 1.62915 |
| L-Leucine | 1.472 | 132.1025 | 131.0952 | 131.0946 | 29892.7 | 50151.73 | 51631.87 | 44501 | 31187.43 | 48806.74 | 64699.12 | 76186.93 | 62498.42 | 64808.12 | 0.612309 | 2.05984 | 1.61907 |
| Oleoylethanolamide | 12.019 | 326.3068 | 325.2992 | 325.2981 | 84.80624 | 119.674 | 108.6065 | 131.8969 | 121.4575 | 83.52733 | 78.01721 | 97.10256 | 55.58603 | 69.13525 | -0.56319 | 2.059812 | 1.61894 |
| Pipecolic acid | 0.879 | 130.087 | 129.0796 | 129.079 | 3807.223 | 5267.226 | 6654.675 | 6665.404 | 6578.222 | 4211.787 | 2489.233 | 4196.354 | 3515.096 | 3740.189 | -0.67452 | 1.999104 | 1.60084 |

| | | | | | | | | | | | | | | | | |
|---|---|---|---|---|---|---|---|---|---|---|---|---|---|---|---|---|
| Metenamine | 14.571 | 141.1142 | 140.1068 | 140.1062 | 1659.925 | 1558.827 | 1393.873 | 1826.845 | 1508.872 | 1340.709 | 1284.956 | 1460.226 | 1380.708 | 1338.129 | -0.22412 | 1.704861 | 1.50043 |
| Eicosapentaenoic acid | 9.407 | 303.2332 | 302.2256 | 302.2246 | 72.05241 | 50.8035 | 52.61233 | 53.71156 | 73.98167 | 42.71516 | 46.66721 | 42.97337 | 44.50881 | 49.93019 | -0.4187 | 1.703592 | 1.49992 |
| Adenine | 1.14 | 136.0623 | 135.0551 | 135.0532 | 287.8382 | 363.6993 | 78.13463 | 331.4913 | 79.26454 | 50.23286 | 48.27455 | 51.43153 | 36.07192 | 57.33555 | -2.22849 | 1.68909 | 1.49433 |
| Xanthine | 1.145 | 153.0415 | 152.0342 | 152.0334 | 180.2484 | 282.5647 | 315.8772 | 334.872 | 260.2263 | 199.9434 | 220.0289 | 191.4486 | 161.9091 | 198.6932 | -0.4991 | 1.645566 | 1.47755 |
| Riboflavin | 3.657 | 377.1469 | 376.1397 | 376.1383 | 56.19563 | 33.6601 | 17.94436 | 43.30095 | 20.46587 | 16.05492 | 9.861357 | 19.46677 | 11.53414 | 11.29728 | -1.33062 | 1.639952 | 1.4753 |
| Putrescine | 0.908 | 89.10836 | 88.10104 | 88.10005 | 602.6527 | 616.0046 | 645.8536 | 672.7837 | 504.314 | 738.8962 | 1028.349 | 655.3956 | 762.8057 | 1048.574 | 0.477194 | 1.632018 | 1.47209 |
| Retinal | 9.412 | 285.2221 | 284.2149 | 284.214 | 18.62876 | 18.07911 | 17.43124 | 16.87302 | 17.49493 | 14.92046 | 17.39931 | 16.63764 | 15.81953 | 16.57158 | -0.12168 | 1.616681 | 1.46595 |
| 3-Methyl sulpholene | 0.828 | 133.0325 | 132.0251 | 132.0245 | 228.1228 | 213.9535 | 386.1733 | 194.8287 | 416.2635 | 506.3145 | 539.6267 | 265.8855 | 549.6249 | 538.0916 | 0.737351 | 1.558756 | 1.44215 |
| Indoleacrylic acid | 2.957 | 188.0717 | 187.064 | 187.0633 | 215.4987 | 261.452 | 241.5304 | 770.7065 | 1289.359 | 1506.025 | 794.3953 | 1547.112 | 1135.412 | 1152.559 | 1.142851 | 1.545633 | 1.43678 |
| Atrazine | 6.909 | 216.1022 | 215.0947 | 215.0938 | 29.01944 | 26.8299 | 27.41704 | 33.29888 | 31.66119 | 22.5445 | 27.6031 | 25.07465 | 25.20373 | 27.87144 | -0.20831 | 1.453113 | 1.39632 |
| 4-Methoxycinnamic acid | 12.178 | 179.071 | 178.0638 | 178.063 | 48.42661 | 27.99492 | 25.12974 | 28.95316 | 27.59379 | 21.67736 | 22.243 | 17.89811 | 21.57003 | 21.64637 | -0.58995 | 1.402745 | 1.37296 |
| Pyridoxamine | 0.913 | 169.0957 | 168.0882 | 168.0899 | 619.4687 | 956.0107 | 1067.316 | 1223.689 | 1359.953 | 857.9109 | 483.0412 | 749.8656 | 677.668 | 732.9477 | -0.57788 | 1.398398 | 1.37091 |
| Nonivamide | 10.072 | 294.2078 | 293.2002 | 293.1991 | 27.32869 | 24.7252 | 13.72861 | 17.39899 | 12.11673 | 12.63734 | 13.93302 | 10.87857 | 9.299756 | 10.50929 | -0.73497 | 1.396881 | 1.37003 |
| L-Tryptophan | 2.818 | 205.0977 | 204.0904 | 204.0899 | 67.77319 | 160.776 | 186.275 | 602.1062 | 405.2403 | 1009.13 | 439.119 | 478.9532 | 752.0764 | 496.232 | 1.158894 | 1.372302 | 1.3584 |
| 3,5-di-tert-Butyl-4-hydroxybenzaldehyde | 9.882 | 235.1702 | 234.1629 | 234.162 | 13.96201 | 13.75784 | 14.23072 | 28.36701 | 17.90873 | 10.71878 | 12.08375 | 9.132289 | 11.35412 | 11.31143 | -0.6923 | 1.34816 | 1.34685 |
| Aspartame | 3.467 | 295.1301 | 294.1227 | 294.1216 | 24.15871 | 25.03362 | 27.1582 | 23.78378 | 16.54609 | 31.80524 | 46.98075 | 27.82962 | 23.78677 | 48.80553 | 0.619072 | 1.308747 | 1.32711 |
| L-Asparagine | 1.058 | 133.0602 | 132.0528 | 132.0535 | 19.14555 | 28.23467 | 31.6141 | 39.80079 | 42.72177 | 18.98058 | 18.8544 | 29.81489 | 20.87421 | 19.158 | -0.58491 | 1.288182 | 1.31663 |
| Methionine | 1.136 | 150.0589 | 149.0516 | 149.051 | 795.0155 | 561.8122 | 1069.81 | 608.8746 | 1162.236 | 1222.107 | 1829.128 | 721.7788 | 1394.181 | 2144.696 | 0.800628 | 1.279291 | 1.31192 |
| palmitoleic acid | 12.313 | 255.2327 | 254.2255 | 254.2246 | 13.84643 | 17.29375 | 4.570508 | 8.807033 | 2.126583 | 4.52072 | 3.282672 | 2.404001 | 2.719463 | 1.375177 | -1.70548 | 1.269069 | 1.30658 |
| L-Threonine | 0.822 | 120.0664 | 119.059 | 119.0582 | 607.1934 | 716.4344 | 647.7406 | 502.5306 | 426.2978 | 620.5062 | 929.1499 | 667.5864 | 702.8468 | 958.6054 | 0.41942 | 1.261312 | 1.30268 |
| Hydroxyphenyllactic acid | 0.921 | 183.0637 | 182.0561 | 182.0579 | 27.95679 | 28.45808 | 14.1125 | 36.53127 | 26.80535 | 24.53158 | 14.4073 | 15.16684 | 17.42514 | 17.56697 | -0.58731 | 1.24644 | 1.29478 |

| | | | | | | | | | | | | | | | | | |
|---|---|---|---|---|---|---|---|---|---|---|---|---|---|---|---|---|---|
| Ophthalmic acid | 1.146 | 290.1355 | 289.1284 | 289.1274 | 645.5809 | 523.7521 | 597.5008 | 606.2516 | 455.2841 | 637.2797 | 1038.046 | 626.3904 | 635.3789 | 931.5763 | 0.451868 | 1.236094 | 1.28929 |
| L-Aspartic acid | 0.793 | 134.0455 | 133.0383 | 133.0375 | 66.20306 | 81.23224 | 86.39382 | 57.81519 | 61.01638 | 61.00277 | 55.86018 | 59.47591 | 60.47829 | 49.17636 | -0.3023 | 1.230179 | 1.28611 |
| N1,N12-Diacetylspermine | 0.819 | 287.2451 | 286.2379 | 286.2369 | 193.1417 | 163.8036 | 197.0145 | 240.2155 | 153.0957 | 255.5746 | 211.0726 | 282.1612 | 306.6491 | 536.7407 | 0.749171 | 1.224141 | 1.28271 |
| Kojic acid | 1.198 | 143.0345 | 142.0272 | 142.0266 | 90.70726 | 95.14927 | 193.6412 | 56.67944 | 74.42413 | 66.78809 | 74.28537 | 34.91895 | 21.80714 | 38.16804 | -1.11361 | 1.177413 | 1.25723 |
| Salicylic acid | 1.101 | 139.0395 | 138.0323 | 138.0317 | 17.13632 | 17.08924 | 9.738239 | 9.525142 | 17.78821 | 10.09952 | 6.288331 | 11.97392 | 10.71122 | 10.712 | -0.51773 | 1.109724 | 1.21809 |
| Aminoadipic acid | 0.871 | 162.0768 | 161.0695 | 161.0688 | 104.1133 | 173.0185 | 185.7423 | 79.48286 | 59.8422 | 250.4987 | 215.4108 | 262.3703 | 78.59415 | 207.3248 | 0.752028 | 1.091613 | 1.20767 |
| Benzoic acid | 0.888 | 123.041 | 122.0337 | 122.0368 | 113.4041 | 129.8103 | 29.19432 | 137.2389 | 18.30659 | 12.65454 | 14.1913 | 74.07016 | 36.08729 | 10.06413 | -1.54098 | 1.083567 | 1.20256 |
| Dihydrothymine | 0.989 | 129.0666 | 128.0592 | 128.0586 | 386.2365 | 355.9277 | 549.6109 | 415.3185 | 413.1667 | 448.5981 | 1059.853 | 414.9214 | 587.4456 | 884.4112 | 0.679268 | 1.063432 | 1.19028 |
| Tetraacetylethylenediamine | 2.934 | 229.1194 | 228.1119 | 228.111 | 57.5407 | 73.33292 | 93.19232 | 100.1695 | 127.5935 | 62.80943 | 63.7679 | 62.02446 | 59.46017 | 81.81972 | -0.45383 | 1.049602 | 1.18187 |
| Tetradecanedioic acid | 9.504 | 259.1914 | 258.1842 | 258.1831 | 3.933755 | 4.535157 | 8.438123 | 5.332166 | 5.440224 | 3.815305 | 4.181248 | 4.745033 | 1.853803 | 4.320078 | -0.54925 | 1.022698 | 1.16494 |
| L-Serine | 0.79 | 106.0509 | 105.0433 | 105.0426 | 74.20507 | 74.62084 | 140.2761 | 57.91661 | 87.59324 | 116.4071 | 95.41602 | 133.5179 | 126.5151 | 109.6751 | 0.420129 | 1.014095 | 1.15931 |
| Deoxyadenosine | 1.356 | 252.11 | 251.1027 | 251.1018 | 345.94 | 146.3894 | 5.896605 | 135.5493 | 4.775083 | 4.070861 | 1.894792 | 36.86283 | 2.890381 | 2.257346 | -3.73441 | 1.013862 | 1.15923 |
| Tretinoin | 11.085 | 301.2171 | 300.2099 | 300.2089 | 36.8166 | 19.54034 | 34.69527 | 30.14303 | 27.95505 | 19.39957 | 27.52979 | 24.06803 | 26.19705 | 19.6186 | -0.35257 | 1.011261 | 1.15782 |
| L-2-amino-3-oxobutanoic acid | 1.146 | 118.0524 | 117.0454 | 117.0426 | 2.919276 | 7.006784 | 4.674793 | 13.89184 | 4.141572 | 16.92774 | 4.329731 | 8.815249 | 14.71559 | 20.04728 | 0.990398 | 1.006708 | 1.15452 |
| Indole-3-carboxaldehyde | 0.899 | 73.53284 | 145.0511 | 145.0528 | 318.4447 | 341.6754 | 63.8651 | 360.7968 | 55.30068 | 37.38727 | 40.0443 | 215.9947 | 107.5597 | 33.92847 | -1.39033 | 0.973911 | 1.1332 |
| Phosphate | 0.751 | 98.98518 | 97.97779 | 97.9769 | 582.7276 | 505.2651 | 512.4954 | 464.0994 | 468.5438 | 381.2925 | 487.8205 | 450.4691 | 472.4814 | 483.0443 | -0.15499 | 0.950669 | 1.11782 |
| Adenosine | 1.271 | 268.1049 | 267.0977 | 267.0968 | 3280.44 | 1152.448 | 81.84623 | 1293.685 | 96.16741 | 56.37804 | 55.61325 | 554.3419 | 53.06438 | 49.10468 | -2.94171 | 0.917666 | 1.09509 |
| Dimethylbenzimidazole | 14.63 | 147.0925 | 146.0851 | 146.0844 | 34.51995 | 27.05188 | 43.03801 | 28.66414 | 39.29829 | 23.99754 | 33.76314 | 25.63883 | 32.41984 | 25.28459 | -0.29044 | 0.909285 | 1.08943 |
| Uridine 5'-diphosphate | 0.694 | 405.011 | 404.0035 | 404.0022 | 127.8966 | 139.5027 | 148.7423 | 174.2206 | 92.76878 | 120.523 | 106.6558 | 114.0578 | 95.55751 | 123.6818 | -0.28551 | 0.905791 | 1.08692 |
| Porphobilinogen | 2.134 | 227.1034 | 226.0962 | 226.0954 | 44.81251 | 56.34277 | 80.84973 | 91.43836 | 126.9449 | 53.70442 | 65.61707 | 51.32694 | 45.0947 | 59.17967 | -0.54237 | 0.891867 | 1.077 |
| L-Acetylcarnitine | 4.256 | 204.124 | 203.1167 | 203.1158 | 6.520388 | 6.486715 | 4.939287 | 8.558541 | 9.291745 | 8.274061 | 17.78917 | 7.107704 | 7.212624 | 21.3986 | 0.787365 | 0.873078 | 1.06352 |

| | | | | | | | | | | | | | | | | | |
|---|---|---|---|---|---|---|---|---|---|---|---|---|---|---|---|---|---|
| D-Alanyl-D-alanine | 0.954 | 161.0929 | 160.0855 | 160.0848 | 536.7399 | 647.1081 | 592.4179 | 429.5801 | 746.1942 | 920.9461 | 1891.873 | 544.273 | 636.8202 | 966.0829 | 0.748626 | 0.855412 | 1.0508 |
| 12-Hydroxydodecanoic acid | 8.81 | 217.1809 | 216.1735 | 216.1725 | 5.86509 | 9.033893 | 13.91255 | 9.030495 | 8.183739 | 9.582418 | 6.990427 | 6.917746 | 4.438647 | 5.33678 | -0.46839 | 0.839952 | 1.0392 |
| Leucylproline | 3.108 | 229.1558 | 228.1483 | 228.1474 | 231.7817 | 205.4848 | 135.4771 | 353.5551 | 270.1316 | 143.8691 | 113.9215 | 240.9905 | 180.2555 | 181.8733 | -0.4748 | 0.830524 | 1.03223 |
| Glutamylhydroxyproline | 1.529 | 261.1092 | 260.1019 | 260.1008 | 11.86956 | 18.32419 | 14.78515 | 11.2954 | 7.918704 | 14.22425 | 30.67386 | 14.19149 | 12.62721 | 23.74924 | 0.572571 | 0.82707 | 1.02969 |
| Dethiobiotin | 3.246 | 215.1396 | 214.1326 | 214.1317 | 88.08327 | 91.56685 | 82.2983 | 120.6883 | 67.17627 | 165.0138 | 69.01602 | 170.0734 | 76.5975 | 161.1348 | 0.512878 | 0.812942 | 1.01894 |
| Pyroglutamic acid | 0.815 | 130.0508 | 129.0433 | 129.0426 | 391.6004 | 337.2446 | 418.4372 | 227.6142 | 290.7628 | 377.0291 | 418.9046 | 420.5703 | 401.4828 | 338.5969 | 0.232244 | 0.793729 | 1.00413 |
| Palmitoylethanolamide | 11.842 | 300.291 | 299.2834 | 299.2824 | 313.8133 | 465.5967 | 493.3161 | 669.3432 | 701.8381 | 444.2507 | 409.4061 | 502.943 | 311.8667 | 385.0174 | -0.3646 | 0.775171 | 0.989501 |
| L-Arginine | 0.849 | 175.1197 | 174.1125 | 174.1117 | 743.4607 | 897.614 | 796.2042 | 1183.19 | 1477.747 | 788.26 | 740.8495 | 744.5787 | 828.6161 | 936.6433 | -0.33601 | 0.758927 | 0.976669 |
| Capryloylglycine | 5.388 | 202.1446 | 201.1372 | 201.1365 | 8.68075 | 7.114103 | 5.94886 | 8.304936 | 6.971549 | 7.254587 | 15.55796 | 7.860716 | 7.249189 | 11.74603 | 0.424018 | 0.751685 | 0.970885 |
| Glutaric acid | 1.104 | 133.0502 | 132.043 | 132.0423 | 61.82071 | 83.93939 | 66.45785 | 49.12887 | 103.0864 | 61.5741 | 48.90618 | 48.38702 | 73.76589 | 54.02058 | -0.34635 | 0.751054 | 0.970226 |
| N-Acetylcadaverine | 1.141 | 145.1343 | 144.1268 | 144.1263 | 1153.583 | 1678.648 | 1377.139 | 2004.365 | 1504.092 | 1868.318 | 1787.472 | 1765.663 | 1675.163 | 1697.013 | 0.188264 | 0.737613 | 0.959461 |
| L-Lysine | 0.898 | 147.1133 | 146.1062 | 146.1055 | 4041.473 | 5752.88 | 7111.913 | 7288.044 | 2568.267 | 4621.12 | 2711.164 | 4507.866 | 3858.144 | 4114.312 | -0.4338 | 0.722135 | 0.946791 |
| Lycoperdic acid | 0.699 | 218.0671 | 217.0595 | 217.0586 | 404.6128 | 572.6742 | 737.8333 | 810.9034 | 764.7305 | 614.4712 | 469.61 | 604.0646 | 443.4857 | 566.7833 | -0.28631 | 0.7199 | 0.944828 |
| Tiglic acid | 1.111 | 101.0606 | 100.0532 | 100.0524 | 442.2856 | 382.8226 | 139.169 | 131.1175 | 204.7116 | 491.4098 | 804.0815 | 140.8578 | 523.609 | 271.1795 | 0.77915 | 0.715344 | 0.941111 |
| Spermidine | 0.925 | 146.166 | 145.1585 | 145.1579 | 2383.883 | 2979.104 | 3319.2 | 3991.222 | 2733.624 | 2181.534 | 3080.058 | 2938.908 | 2127.279 | 2759.559 | -0.23542 | 0.686568 | 0.916674 |
| Xanthosine | 0.918 | 285.0855 | 284.0782 | 284.0757 | 24.76498 | 32.56805 | 29.75104 | 32.69962 | 15.94989 | 30.42483 | 34.20802 | 37.52951 | 42.26324 | 22.44236 | 0.297929 | 0.671911 | 0.903892 |
| Diphenylamine | 8.987 | 170.0973 | 169.0899 | 169.0891 | 90.57908 | 82.07549 | 80.61697 | 101.4383 | 91.81714 | 74.90592 | 86.19414 | 74.22204 | 79.20073 | 95.42609 | -0.1233 | 0.661126 | 0.894664 |
| Creatinine | 14.567 | 114.0672 | 113.0598 | 113.0589 | 35.54907 | 34.93695 | 34.07544 | 42.44084 | 38.71778 | 33.90604 | 28.22447 | 40.57479 | 34.04164 | 33.10872 | -0.12882 | 0.620455 | 0.858007 |
| 2-Ethylhexanoic acid | 15.337 | 145.1229 | 144.1156 | 144.115 | 44.6653 | 46.76159 | 53.10727 | 48.38804 | 4.233564 | 32.26963 | 41.615 | 37.46254 | 3.148321 | 8.93368 | -0.67565 | 0.604752 | 0.843351 |
| Diethylphosphate | 5.251 | 155.0473 | 154.04 | 154.0395 | 18.11776 | 43.87319 | 84.32978 | 16.95555 | 13.41861 | 16.90295 | 22.76317 | 19.52154 | 19.31051 | 14.57959 | -0.92475 | 0.603126 | 0.842016 |
| Glycerol 3-phosphate | 0.734 | 173.0219 | 172.0144 | 172.0137 | 1432.983 | 1697.557 | 2063.043 | 1497.544 | 1306.947 | 1379.252 | 1675.528 | 1215.97 | 1408.381 | 1383.789 | -0.17939 | 0.599432 | 0.83857 |

| | | | | | | | | | | | | | | | | | |
|---|---|---|---|---|---|---|---|---|---|---|---|---|---|---|---|---|---|
| Pantothenic acid | 2.34 | 220.1188 | 219.1116 | 219.1107 | 222.5813 | 294.1319 | 332.3995 | 136.9933 | 219.6248 | 212.2391 | 668.5176 | 213.4795 | 222.6736 | 510.3017 | 0.599736 | 0.598844 | 0.837994 |
| Aprindine | 3.209 | 323.2452 | 322.2379 | 322.2409 | 1.02684 | 1.152092 | 65.52661 | 1.072007 | 16.64372 | 7.273156 | 2.747708 | 0.853579 | 2.960333 | 2.100559 | -2.42237 | 0.523608 | 0.764765 |
| N-Acetylputrescine | 1.119 | 131.1187 | 130.1113 | 130.1106 | 6213.137 | 12234.32 | 10599.78 | 6849.088 | 6328.451 | 9231.976 | 11473.99 | 10124.24 | 9591.579 | 9071.822 | 0.229153 | 0.519648 | 0.760737 |
| Arachidonic acid | 11.132 | 305.2489 | 304.2414 | 304.2402 | 30.11063 | 14.1522 | 22.16307 | 23.34038 | 25.59126 | 17.41485 | 31.69014 | 12.89969 | 18.71276 | 10.71932 | -0.33527 | 0.497904 | 0.738197 |
| Tyramine | 1.599 | 138.092 | 137.0847 | 137.0841 | 1235.728 | 1206.524 | 878.3296 | 1271.685 | 1706.456 | 1503.478 | 1495.863 | 1188.583 | 1628.109 | 1278.495 | 0.171648 | 0.476985 | 0.716173 |
| 2-Hexenal | 1.103 | 99.08146 | 98.07413 | 98.07316 | 488.2095 | 317.852 | 121.9758 | 142.1609 | 168.0413 | 131.3662 | 222.1686 | 230.4206 | 155.0207 | 133.8955 | -0.50445 | 0.464096 | 0.702077 |
| Panthenol | 2.373 | 206.1395 | 205.1322 | 205.1314 | 0.83276 | 0.549891 | 0.657998 | 0.810078 | 1.456059 | 0.807434 | 32.48849 | 0.659776 | 0.777668 | 0.559463 | 3.034692 | 0.445675 | 0.682212 |
| 1,2-Benzisothiazol-3(2H)-one | 5.39 | 152.0172 | 151.0098 | 151.0092 | 121.2578 | 55.12754 | 54.93171 | 55.0621 | 53.32235 | 42.05391 | 94.16086 | 27.29158 | 44.54723 | 46.39985 | -0.41687 | 0.445631 | 0.682022 |
| Hypotaurine | 4.199 | 110.0273 | 109.02 | 109.0197 | 0.732614 | 0.744289 | 0.793889 | 0.730829 | 0.807135 | 0.639852 | 0.70544 | 21.54373 | 0.621357 | 0.645883 | 2.665005 | 0.445405 | 0.681919 |
| LysoPC(16:0/0:0) | 10.299 | 496.3416 | 495.3344 | 495.3325 | 7.596629 | 8.671709 | 6.96409 | 8.905763 | 11.81853 | 45.77649 | 7.034709 | 8.708003 | 7.621973 | 10.95956 | 0.865732 | 0.438531 | 0.674299 |
| Heptadecanoic acid | 1.105 | 271.2641 | 270.2567 | 270.2559 | 31.60913 | 11.45871 | 4.016569 | 17.3548 | 23.98429 | 12.555 | 13.12602 | 17.24187 | 10.95691 | 11.48629 | -0.43589 | 0.424887 | 0.658726 |
| Diaminopimelic acid | 0.917 | 191.105 | 190.0976 | 190.0954 | 308.95 | 371.8305 | 126.0545 | 359.2419 | 407.3376 | 282.5272 | 321.7263 | 227.243 | 200.8076 | 289.1651 | -0.25176 | 0.418989 | 0.651865 |
| Methylmalonic acid | 1.249 | 119.0348 | 118.0275 | 118.0266 | 104.615 | 90.92724 | 81.88095 | 117.8764 | 91.43958 | 94.45609 | 169.3772 | 101.249 | 76.5741 | 123.4817 | 0.215455 | 0.411774 | 0.643947 |
| Isobutyric acid | 1.112 | 89.06077 | 88.05345 | 88.05243 | 305.3091 | 112.0347 | 137.8185 | 112.1698 | 145.2556 | 89.15512 | 133.0727 | 212.9804 | 95.65355 | 82.33539 | -0.40617 | 0.411449 | 0.643413 |
| Nicotinic acid | 1.111 | 124.0402 | 123.0328 | 123.032 | 636.9261 | 412.885 | 291.2778 | 873.0653 | 568.4493 | 579.2129 | 922.9109 | 1024.345 | 741.6042 | 255.1735 | 0.34047 | 0.393028 | 0.622118 |
| Deoxyribose | 1.128 | 135.0657 | 134.0587 | 134.0579 | 161.4924 | 122.1984 | 78.70882 | 120.2098 | 113.8315 | 136.1986 | 99.85311 | 117.8033 | 90.22233 | 81.59173 | -0.18222 | 0.383041 | 0.609995 |
| Diflunisal | 11.764 | 251.0479 | 250.0405 | 250.0442 | 5.397139 | 4.143845 | 6.789882 | 4.544281 | 5.444547 | 3.288093 | 5.031345 | 4.636425 | 5.88814 | 4.807628 | -0.1542 | 0.382831 | 0.610274 |
| Cytosine | 14.531 | 112.0514 | 111.0441 | 111.0433 | 17.9531 | 19.85303 | 16.02776 | 18.97899 | 17.67399 | 9.334137 | 13.11268 | 18.10247 | 22.76216 | 17.05343 | -0.17114 | 0.378969 | 0.605546 |
| Linolenelaidic acid | 9.902 | 279.2329 | 278.2255 | 278.2246 | 6.560214 | 1.534843 | 4.022773 | 17.26535 | 2.936565 | 1.70369 | 0.88011 | 5.739751 | 5.82653 | 5.752399 | -0.69947 | 0.360109 | 0.582798 |
| Tyrosine methylester | 2.947 | 196.0978 | 195.0906 | 195.0895 | 1.799347 | 2.145666 | 6.583082 | 1.925418 | 7.727043 | 5.856297 | 7.557278 | 1.949897 | 3.711435 | 8.129905 | 0.430896 | 0.355055 | 0.576285 |
| Glutamine | 0.796 | 147.0771 | 146.0699 | 146.0691 | 270.3018 | 250.1987 | 366.0567 | 185.5508 | 255.5699 | 303.7562 | 259.7787 | 323.0035 | 310.3205 | 259.7702 | 0.133729 | 0.354207 | 0.575211 |
| Phenyllactic acid | 0.995 | 167.0683 | 166.0612 | 166.063 | 21.18625 | 16.87997 | 8.855163 | 15.45093 | 29.02951 | 18.40245 | 16.43272 | 12.13533 | 14.73315 | 15.74775 | -0.23893 | 0.349189 | 0.568893 |

| | | | | | | | | | | | | | | | | | |
|---|---|---|---|---|---|---|---|---|---|---|---|---|---|---|---|---|---|
| dGMP | 0.8 | 348.0714 | 347.0642 | 347.0631 | 37.49359 | 63.39258 | 140.261 | 66.9349 | 115.2306 | 103.258 | 91.4697 | 85.43531 | 104.5774 | 112.0281 | 0.23085 | 0.328697 | 0.543132 |
| Hypoxanthine | 1.138 | 137.0465 | 136.0391 | 136.0385 | 132.6114 | 376.0239 | 457.301 | 636.5555 | 579.8689 | 412.0503 | 306.1418 | 455.7991 | 340.2077 | 325.8895 | -0.24611 | 0.316251 | 0.527315 |
| 5'-Methylthioadenosine | 3.148 | 298.0981 | 297.0906 | 297.0896 | 1139.146 | 1022.889 | 1711.159 | 1175.315 | 1359.567 | 1386.854 | 1702.332 | 1066.516 | 1160.547 | 1759.561 | 0.143004 | 0.31093 | 0.520252 |
| 2-Hydroxyvalerate | 3.16 | 119.0738 | 118.0664 | 118.063 | 34.20674 | 39.91935 | 30.78421 | 36.92489 | 41.23253 | 39.18406 | 28.28813 | 34.66063 | 35.13069 | 36.36768 | -0.07635 | 0.309511 | 0.518382 |
| 2-Hydroxycaproic acid | 15.334 | 133.0865 | 132.0793 | 132.0786 | 237.6617 | 25.21988 | 308.4014 | 253.0181 | 136.7526 | 98.04194 | 219.2454 | 215.2166 | 110.6637 | 118.0192 | -0.33637 | 0.299704 | 0.505833 |
| Oleamide | 12.443 | 282.2802 | 281.2728 | 281.2719 | 1131.733 | 275.1185 | 222.6405 | 419.6389 | 249.073 | 158.6332 | 403.0135 | 276.7221 | 263.2122 | 558.1398 | -0.46957 | 0.293474 | 0.49742 |
| L-Lactic acid | 1.1 | 91.04005 | 90.03275 | 90.03169 | 16.61494 | 10.59304 | 8.448887 | 13.46552 | 10.9898 | 8.110697 | 11.3568 | 15.06856 | 9.562104 | 9.692387 | -0.1603 | 0.291595 | 0.494772 |
| Phosphohydroxypyruvic acid | 14.819 | 184.9866 | 183.9791 | 183.9773 | 869.1479 | 785.7185 | 651.4167 | 436.7293 | 645.6338 | 606.5281 | 755.7637 | 708.5437 | 516.8293 | 513.8286 | -0.12775 | 0.272176 | 0.468204 |
| N-Undecanoylglycine | 12.735 | 244.1917 | 243.1844 | 243.1834 | 284.4968 | 152.711 | 115.4282 | 153.8633 | 424.4075 | 382.1407 | 97.48217 | 88.72462 | 205.4233 | 110.4322 | -0.35503 | 0.257231 | 0.447663 |
| Glutamylleucine | 1.623 | 261.1456 | 260.1382 | 260.1372 | 30.67213 | 30.83481 | 27.04461 | 22.07192 | 49.96964 | 23.47559 | 43.39664 | 32.6538 | 28.56927 | 54.4923 | 0.185179 | 0.248869 | 0.436016 |
| N-Acetylhistamine | 0.819 | 154.0974 | 153.0896 | 153.0902 | 53.57218 | 53.89772 | 45.9578 | 88.11113 | 82.98751 | 42.48022 | 104.9022 | 37.69475 | 41.6358 | 52.56498 | -0.21663 | 0.246224 | 0.432132 |
| Indoleacetic acid | 4.454 | 176.0714 | 175.0642 | 175.0633 | 39.70721 | 36.04787 | 47.75063 | 45.36385 | 72.45007 | 47.32396 | 48.92917 | 42.09052 | 44.99854 | 38.77959 | -0.11959 | 0.237215 | 0.41925 |
| Linoleamide | 11.74 | 280.2643 | 279.257 | 279.2562 | 176.7306 | 63.16405 | 69.88037 | 114.4668 | 94.3807 | 51.20132 | 97.92903 | 78.14091 | 97.64373 | 125.1082 | -0.20469 | 0.236528 | 0.418466 |
| Phenylacetaldehyde | 1.582 | 121.0656 | 120.0583 | 120.0575 | 359.8494 | 139.6466 | 337.1055 | 395.9751 | 175.3122 | 241.4869 | 365.5968 | 179.0819 | 73.70363 | 345.0053 | -0.22465 | 0.220375 | 0.394786 |
| Caprolactam | 1.139 | 114.0921 | 113.0847 | 113.0841 | 6212.193 | 12235.37 | 10607.89 | 6853.862 | 6335.281 | 9234.283 | 11473.41 | 10125.54 | 6266.6 | 9073.239 | 0.128285 | 0.209629 | 0.378908 |
| Shikimic acid | 0.718 | 175.0609 | 174.0535 | 174.0528 | 3.275323 | 3.332607 | 3.267796 | 8.916073 | 4.197911 | 5.511807 | 2.902666 | 3.698331 | 4.641216 | 3.123017 | -0.20989 | 0.209534 | 0.378645 |
| Anandamide | 11.307 | 348.2908 | 347.2834 | 347.2824 | 6.786662 | 6.955981 | 8.737846 | 9.29586 | 10.68374 | 10.89446 | 7.189575 | 10.24164 | 5.268251 | 5.290079 | -0.12693 | 0.204756 | 0.371113 |
| 3-Oxohexanoic acid | 2.284 | 131.0709 | 130.0636 | 130.063 | 40.15549 | 25.38606 | 37.16639 | 64.9735 | 117.6104 | 36.66457 | 49.17346 | 91.36005 | 24.67452 | 31.78553 | -0.28804 | 0.204613 | 0.370963 |
| L-Phenylalanine | 2.208 | 166.0872 | 165.0798 | 165.079 | 5642.543 | 10371.33 | 7883.421 | 12568.2 | 10010.99 | 8972.536 | 10604.82 | 9866.402 | 8445.882 | 11948.92 | 0.100761 | 0.20292 | 0.368556 |
| Cinnamic acid | 2.211 | 149.0605 | 148.0532 | 148.0524 | 5710.894 | 10431.47 | 7965.124 | 12694.71 | 10066.54 | 9023.512 | 10664.31 | 9958.945 | 8496.227 | 12019.35 | 0.097979 | 0.196881 | 0.359343 |
| Citrulline | 0.799 | 176.1036 | 175.0964 | 175.0957 | 55.60884 | 78.38666 | 86.33897 | 60.05243 | 61.95469 | 79.26322 | 51.74336 | 103.9493 | 80.1612 | 53.67195 | 0.107359 | 0.185056 | 0.341084 |

| | | | | | | | | | | | | | | | | |
|---|---|---|---|---|---|---|---|---|---|---|---|---|---|---|---|---|
| L-Histidine | 0.855 | 156.0775 | 155.0702 | 155.0695 | 287.5277 | 693.786 | 931.8206 | 1051.997 | 962.1204 | 834.2904 | 763.3206 | 953.2384 | 395.4135 | 592.9737 | -0.15008 | 0.181768 | 0.335779 |
| L-Tyrosine | 1.192 | 182.0822 | 181.0747 | 181.0739 | 1018.252 | 1420.596 | 1723.587 | 1730.586 | 1489.016 | 1259.452 | 1458.19 | 1392.756 | 1158.29 | 1744.324 | -0.07398 | 0.17721 | 0.328815 |
| Pantethine | 3.997 | 555.2532 | 554.246 | 554.2444 | 0.642173 | 12.06881 | 37.73631 | 35.17346 | 62.00764 | 42.95907 | 15.15332 | 27.25535 | 21.70965 | 14.69204 | -0.27782 | 0.170455 | 0.317977 |
| Thymine | 0.82 | 127.0509 | 126.0435 | 126.0429 | 6.987289 | 12.468 | 15.74677 | 10.02176 | 13.23048 | 11.42982 | 10.53813 | 12.2782 | 14.89327 | 12.70543 | 0.081344 | 0.158959 | 0.29932 |
| Cytidine | 14.819 | 122.548 | 243.0811 | 243.0855 | 387.6698 | 375.2976 | 382.7987 | 456.0528 | 503.7955 | 353.4646 | 420.0178 | 438.0579 | 433.3953 | 536.8773 | 0.051286 | 0.152289 | 0.288402 |
| Benzothiazole | 5.825 | 136.0222 | 135.0149 | 135.0143 | 20480.16 | 22013.08 | 20414.68 | 18175.53 | 21786.33 | 22708.2 | 21515.79 | 20766.24 | 19642.9 | 15672.32 | -0.03642 | 0.142162 | 0.271431 |
| L-Glutamic acid | 0.803 | 148.0611 | 147.0537 | 147.0532 | 1732.684 | 2203.511 | 2021.974 | 1715.83 | 1696.318 | 1723.645 | 1667.416 | 2471.913 | 1628.829 | 1514.977 | -0.05709 | 0.140034 | 0.267974 |
| Uridine diphosphate-N-acetylglucosamine | 0.695 | 608.0904 | 607.0831 | 607.0816 | 13.49802 | 10.33767 | 11.99333 | 7.313789 | 4.820063 | 5.572391 | 12.69696 | 8.056838 | 8.181208 | 18.26982 | 0.137997 | 0.133995 | 0.257895 |
| N8-Acetylspermidine | 0.779 | 188.1765 | 187.1692 | 187.1685 | 3280.01 | 5603.456 | 6069.351 | 6835.589 | 4028.316 | 3649.867 | 7510.274 | 4241.778 | 3787.407 | 5073.898 | -0.08953 | 0.121316 | 0.236295 |
| N-Phenylacetylglutamic acid | 4.384 | 266.1035 | 265.096 | 265.095 | 31.25722 | 56.66127 | 44.38945 | 62.40545 | 48.86862 | 41.6292 | 50.64829 | 73.54777 | 41.1514 | 48.67012 | 0.069744 | 0.113507 | 0.222811 |
| trans-Aconitic acid | 0.819 | 175.0235 | 174.0162 | 174.0164 | 6.056525 | 17.60857 | 21.50361 | 10.48675 | 23.0333 | 15.28365 | 19.69579 | 16.24783 | 6.854392 | 27.12059 | 0.114734 | 0.104993 | 0.207708 |
| Oxidized glutathione | 1.142 | 307.0841 | 612.154 | 612.152 | 330.9261 | 108.1741 | 108.6236 | 54.32168 | 40.49096 | 53.99769 | 326.1156 | 63.67047 | 81.83764 | 215.7201 | 0.20636 | 0.097936 | 0.19495 |
| Azelaic acid | 11.679 | 189.1128 | 188.1056 | 188.1049 | 42.57201 | 14.76398 | 21.59341 | 19.29829 | 19.32888 | 19.17207 | 21.13417 | 17.6141 | 17.57317 | 34.45158 | -0.09657 | 0.096461 | 0.192402 |
| Niacinamide | 1.143 | 123.0562 | 122.0488 | 122.048 | 38.69298 | 70.23692 | 2512.821 | 72.58588 | 1988.192 | 971.6151 | 1797.935 | 107.6395 | 1143.348 | 1414.557 | 0.215017 | 0.090543 | 0.181367 |
| Methionine sulfoxide | 1.135 | 166.0539 | 165.0466 | 165.046 | 253.9895 | 181.7256 | 262.9143 | 127.0817 | 380.1323 | 265.233 | 320.8065 | 161.7864 | 224.6867 | 287.2749 | 0.063138 | 0.077273 | 0.156953 |
| FAD | 2.852 | 786.1663 | 785.1586 | 785.1571 | 8.981405 | 11.79351 | 16.85216 | 16.64087 | 18.92218 | 15.91688 | 17.77212 | 13.45767 | 12.78123 | 15.38634 | 0.041274 | 0.075557 | 0.153663 |
| Monobutyl phthalate | 10.599 | 223.0975 | 222.0901 | 222.0892 | 48.6674 | 50.62408 | 59.44498 | 57.15197 | 64.73086 | 72.62215 | 58.2284 | 47.47434 | 57.27508 | 39.06099 | -0.03096 | 0.067516 | 0.138362 |
| Tridecanoylglycine | 7.519 | 272.223 | 271.2158 | 271.2147 | 18.55554 | 3.558036 | 4.836312 | 26.14467 | 118.3402 | 128.9066 | 4.633942 | 3.08545 | 47.94875 | 9.420373 | 0.178361 | 0.049671 | 0.103782 |
| L-Theanine | 1.597 | 175.1087 | 174.1014 | 174.1004 | 26.75936 | 24.82697 | 14.99039 | 28.62859 | 31.85997 | 21.26036 | 20.30611 | 27.12055 | 23.59052 | 37.50927 | 0.030574 | 0.045428 | 0.095256 |
| N-Lauroylglycine | 6.97 | 258.2072 | 257.2001 | 257.1991 | 6.172401 | 1.239163 | 1.611813 | 6.064995 | 25.95598 | 24.40605 | 2.732737 | 2.15486 | 11.18625 | 4.488151 | 0.131717 | 0.044733 | 0.093941 |
| N'-Formylkynurenine | 1.394 | 237.0878 | 236.0805 | 236.0797 | 19.38687 | 27.45404 | 22.38013 | 65.69342 | 36.70116 | 28.54698 | 24.87433 | 66.42501 | 18.28724 | 26.30914 | -0.0616 | 0.042115 | 0.08857 |
| Monoethylhexyl phthalic acid | 10.616 | 279.1603 | 278.1527 | 278.1518 | 3975.156 | 3852.802 | 4374.49 | 4438.427 | 5555.793 | 5031.558 | 4751.556 | 3798.798 | 4256.201 | 4176.577 | -0.01188 | 0.034127 | 0.072383 |
| 17alpha-Estradiol | 10.635 | 273.1857 | 272.1786 | 272.1776 | 13.64895 | 10.48875 | 23.20698 | 15.14855 | 18.55066 | 12.08774 | 23.34815 | 17.128 | 13.79877 | 15.94909 | 0.022395 | 0.030271 | 0.064417 |

| | | | | | | | | | | | | | | | | |
|---|---|---|---|---|---|---|---|---|---|---|---|---|---|---|---|---|
| Docosanamide | 13.555 | 340.3585 | 339.3513 | 339.3501 | 39.81 | 62.69558 | 18.88799 | 16.58482 | 60.62949 | 26.9421 | 36.13895 | 50.02398 | 30.53318 | 58.32561 | 0.024174 | 0.020047 | 0.043466 |
| Palmitic amide | 12.263 | 256.2646 | 255.2571 | 255.2562 | 675.3872 | 282.4621 | 216.958 | 685.564 | 508.2377 | 520.0132 | 225.2897 | 365.8831 | 602.1204 | 640.0887 | -0.0093 | 0.008302 | 0.018346 |
| Sulcatol | 8.583 | 129.128 | 128.1207 | 128.1201 | 30.485 | 27.85589 | 28.33566 | 25.40532 | 32.8166 | 49.01775 | 25.73884 | 15.83795 | 32.03454 | 22.47059 | 0.002002 | 0.002345 | 0.005205 |
| Adipic acid | 0.722 | 147.0657 | 146.0586 | 146.0579 | 20.65388 | 12.70775 | 13.06125 | 12.79936 | 29.28153 | 16.21618 | 14.67577 | 11.09382 | 21.31307 | 25.14456 | -0.00098 | 0.000989 | 0.002281 |

**Figure S1. Genetic and proteomic comparison of RS218 and IHE3034, related to Fig. 1. and Fig. 2.**

**A.** Dot plot of the genomic alignment of the chromosomes of RS218 and IHE3034.

**B.** Two-way amino acid identity between the IHE3034 and RS218 datasets of proteins based on Rodríguez-R and Konstantinidis methodology.

**C.** Hierarchical clustering of the protein samples extracted from IHE3034 and RS218.

**D.** Comparison of the expression profiles of the detected but not validated protein species that participate in the regulation of cellular morphology. The FtsH protease and its regulators HflK and HflC; the SPOR domain protein DamX are upregulated in RS218 compared to IHE3034. The levels of MinC were equally present in IHE3034 and RS218, but MinD and MinE were below detection level in the case of RS218.

**E.** Comparative analysis between cellular morphology and use of citrate by ExPEC pyelonephritis isolates.

**F.** Comparative analysis between cellular morphology and use of citrate by ExPEC cystitis isolates.

**G.** Comparative analysis between cellular morphology and use of citrate by ExPEC meningitis isolates.

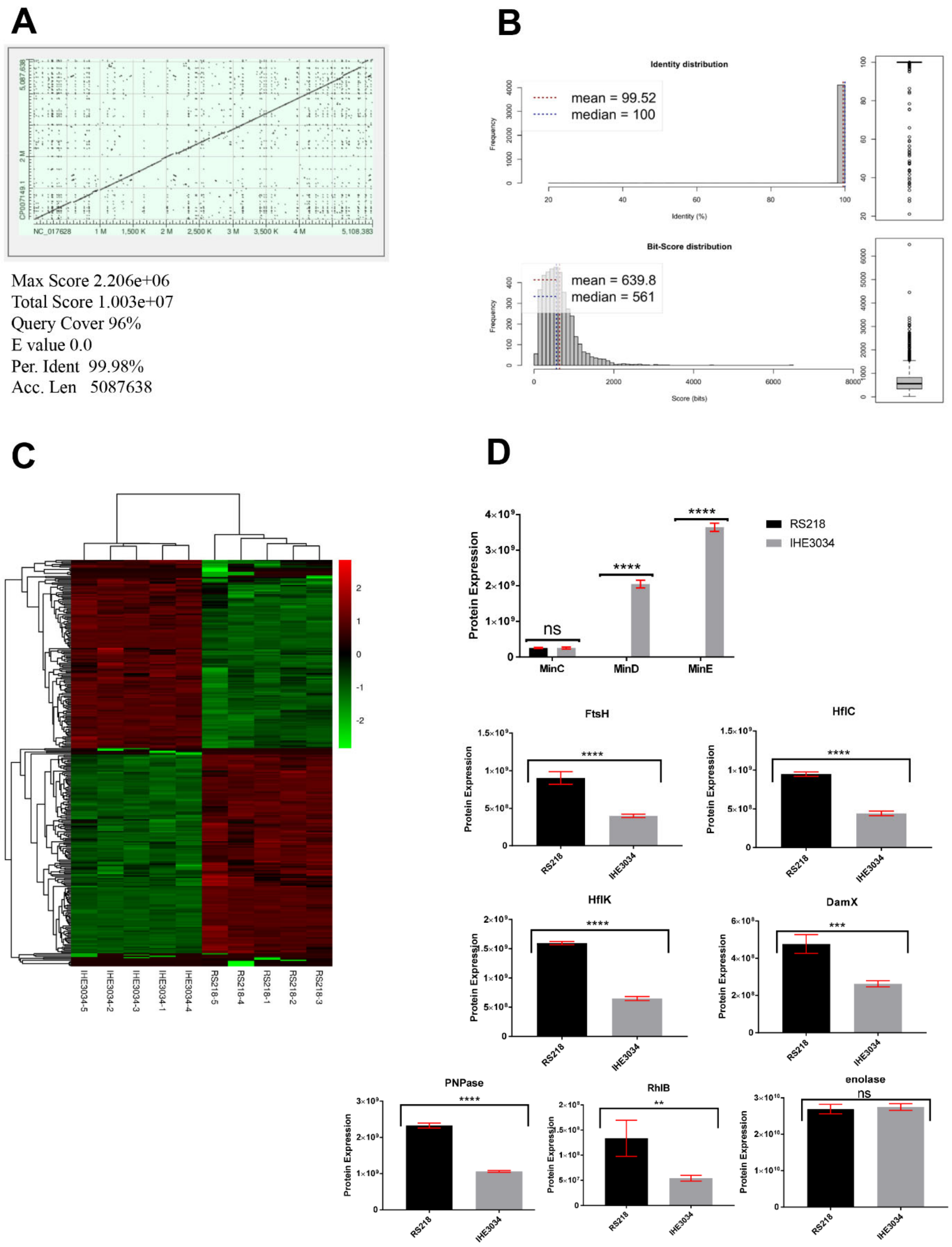
A
Max Score 2.206e+06
Total Score 1.003e+07
Query Cover 96%
E value 0.0
Per. Ident 99.98%
Acc. Len 5087638
B
Identity distribution
mean = 99.52
median = 100
Frequency
Identity (%)
Bit-Score distribution
mean = 639.8
median = 561
Score (bits)
C
IHE3034-5
IHE3034-2
IHE3034-3
IHE3034-1
IHE3034-4
RS218-5
RS218-4
RS218-1
RS218-2
RS218-3
D
RS218
IHE3034
Protein Expression
ns
****
MinC
MinD
MinE
FtsH
HflC
HflK
DamX
***
PNPase
RhlB
**
enolase

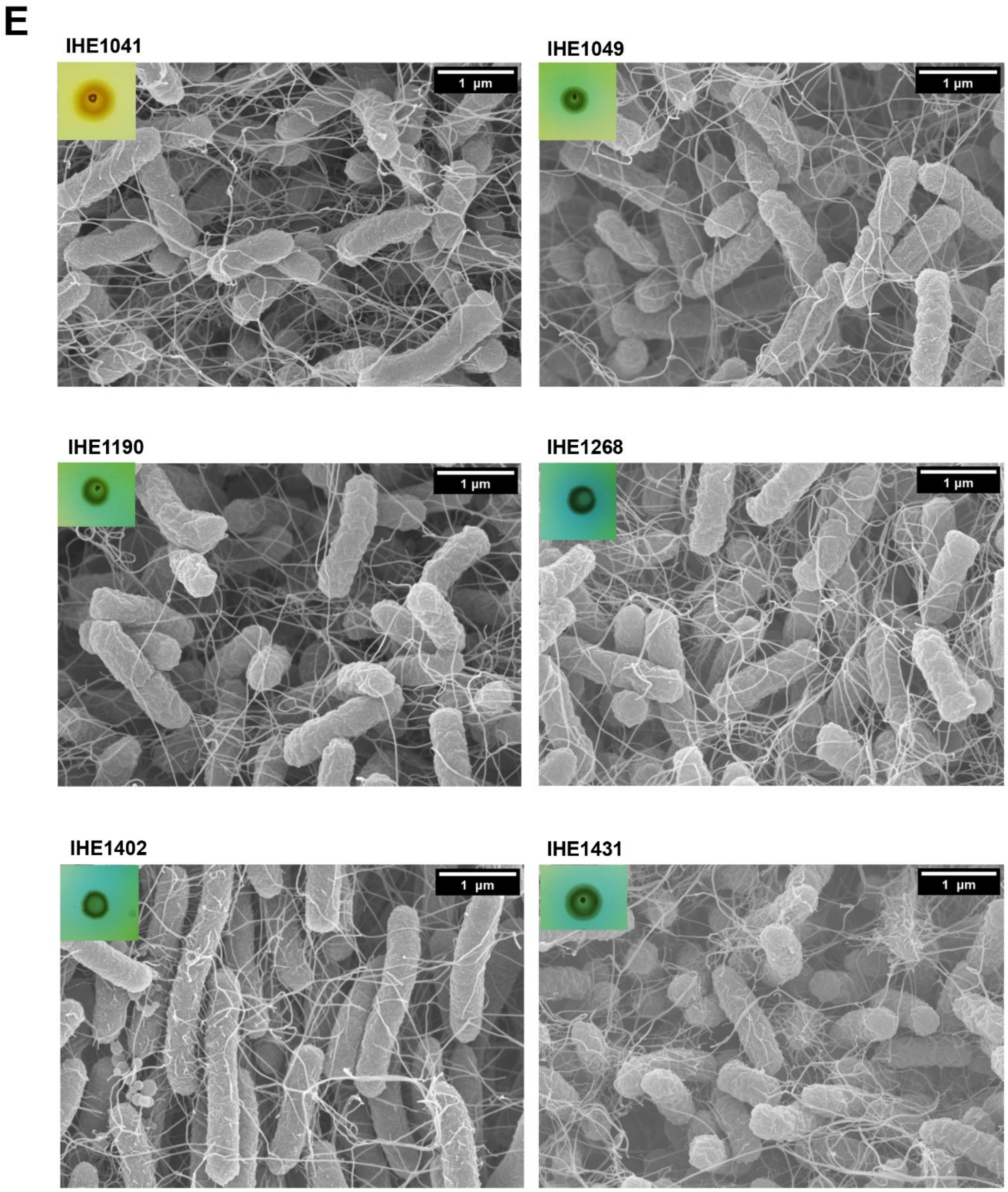
E
IHE1041
1 µm
IHE1049
1 µm
IHE1190
1 µm
IHE1268
1 µm
IHE1402
1 µm
IHE1431
1 µm

F

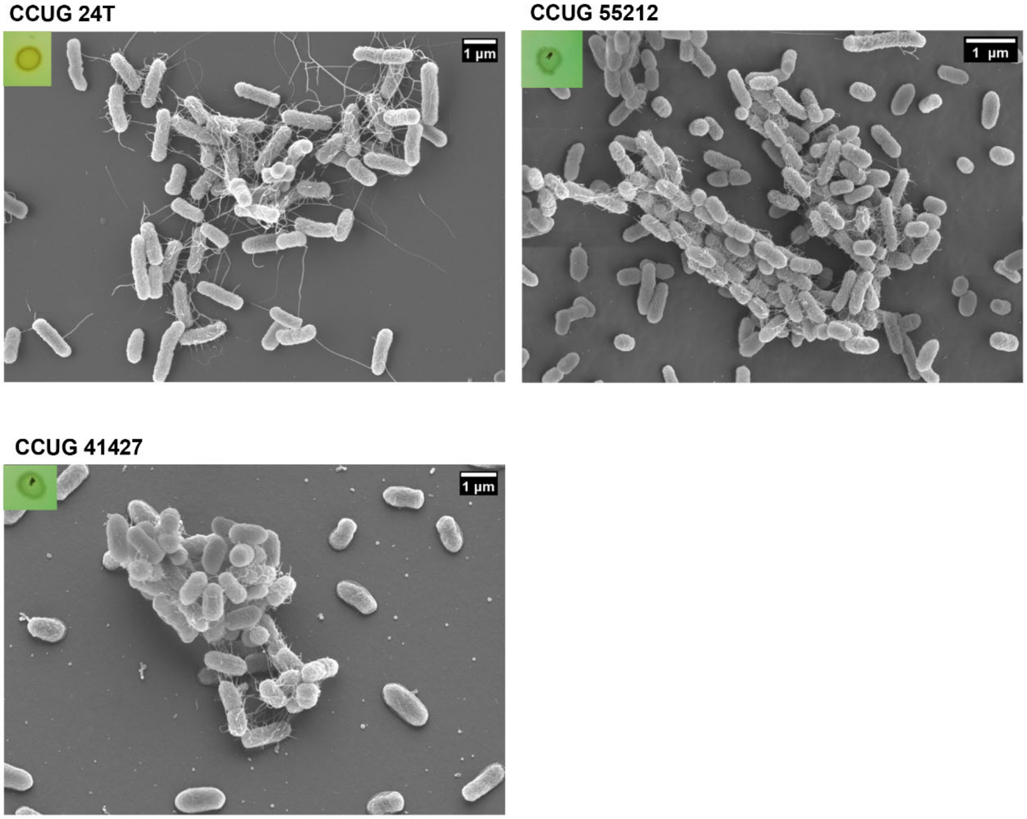

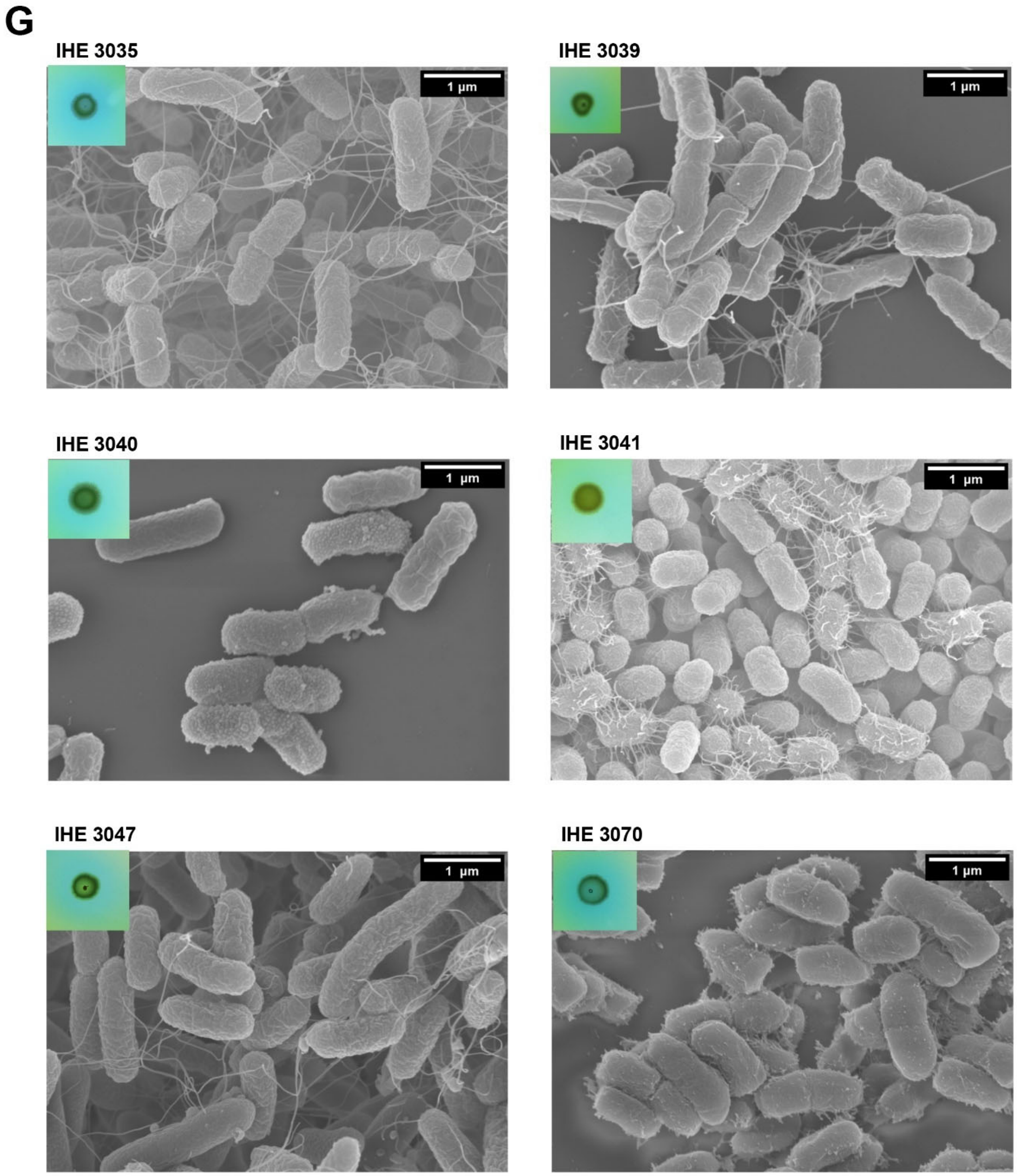
G
IHE 3035
1 µm
IHE 3039
1 µm
IHE 3040
1 µm
IHE 3041
1 µm
IHE 3047
1 µm
IHE 3070
1 µm

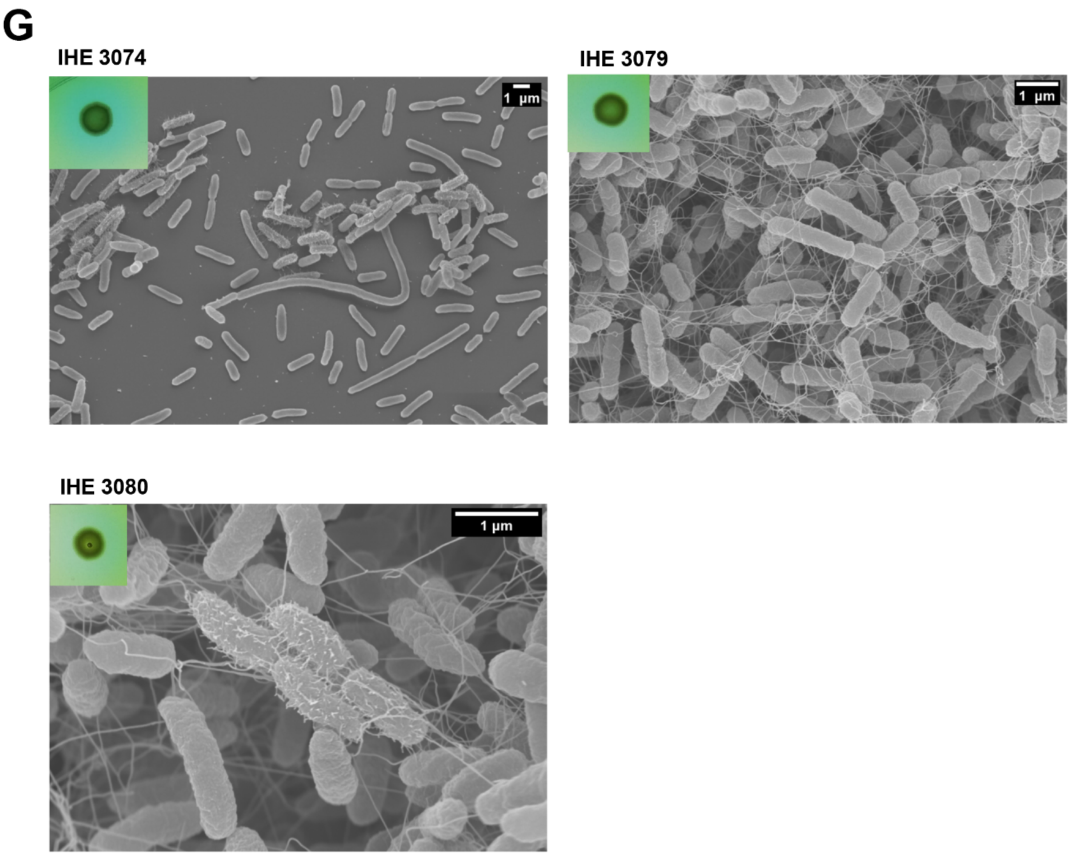
G
IHE 3074
1 µm
IHE 3079
1 µm
IHE 3080
1 µm

**Figure S2. SfaC- and SfaY-dependent phase-variation events in different ExPEC strains, related to Fig. 3 and Fig. 4.**

**A.** SEM images of the IHE3034*ΔsfaY*/EV and IHE3034*ΔsfaY*/pSfaY strains.

**B.** Comparison between the IHE3034 ON strains in the presence and absence of pSfaY.

**C.** Quantification of the detected LexA bands shown in **Fig. 4C**.

**D.** Comparison between the IHE3034*Δhns* ON strains in the presence and absence of pSfaY. The c-di-GMP levels of each strain are displayed next to the panel.

**E.** Comparative analysis between cellular morphology and use of citrate by the ON RS218 strains.

**F.** SEM image of the RS218*ΔsfaY* bacteria.

**G.** SEM image of the RS218*ΔsulA* bacteria. The ratio of the elongated bacteria (length > 2.5 µm) to the total number of bacteria detected in the wild-type colonies versus the mutant ones is displayed below the picture. (Bacterial length was estimated from the SEM images of three biological replicates.)

**H.** Comparative analysis between cellular morphology and use of citrate by the ON UTI89 strains.

**I.** Comparative analysis between cellular morphology and use of citrate by the ON IHE3040 strains.

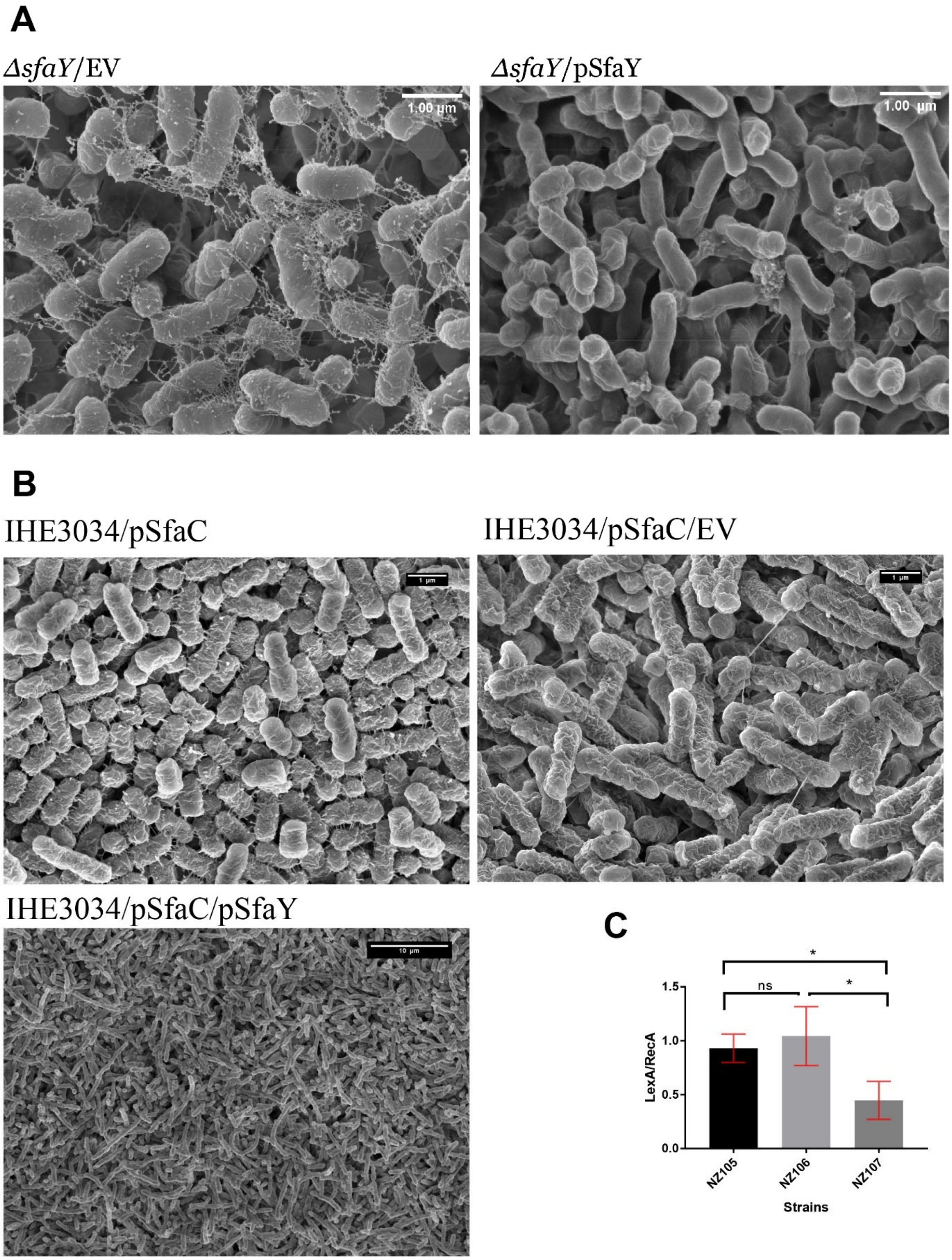
A
ΔsfaY/EV
1.00 µm
ΔsfaY/pSfaY
1.00 µm
B
IHE3034/pSfaC
1 µm
IHE3034/pSfaC/EV
1 µm
IHE3034/pSfaC/pSfaY
10 µm
C
LexA/RecA
1.5
1.0
0.5
0.0
ns
*
*
NZ105
NZ106
NZ107
Strains

**D**

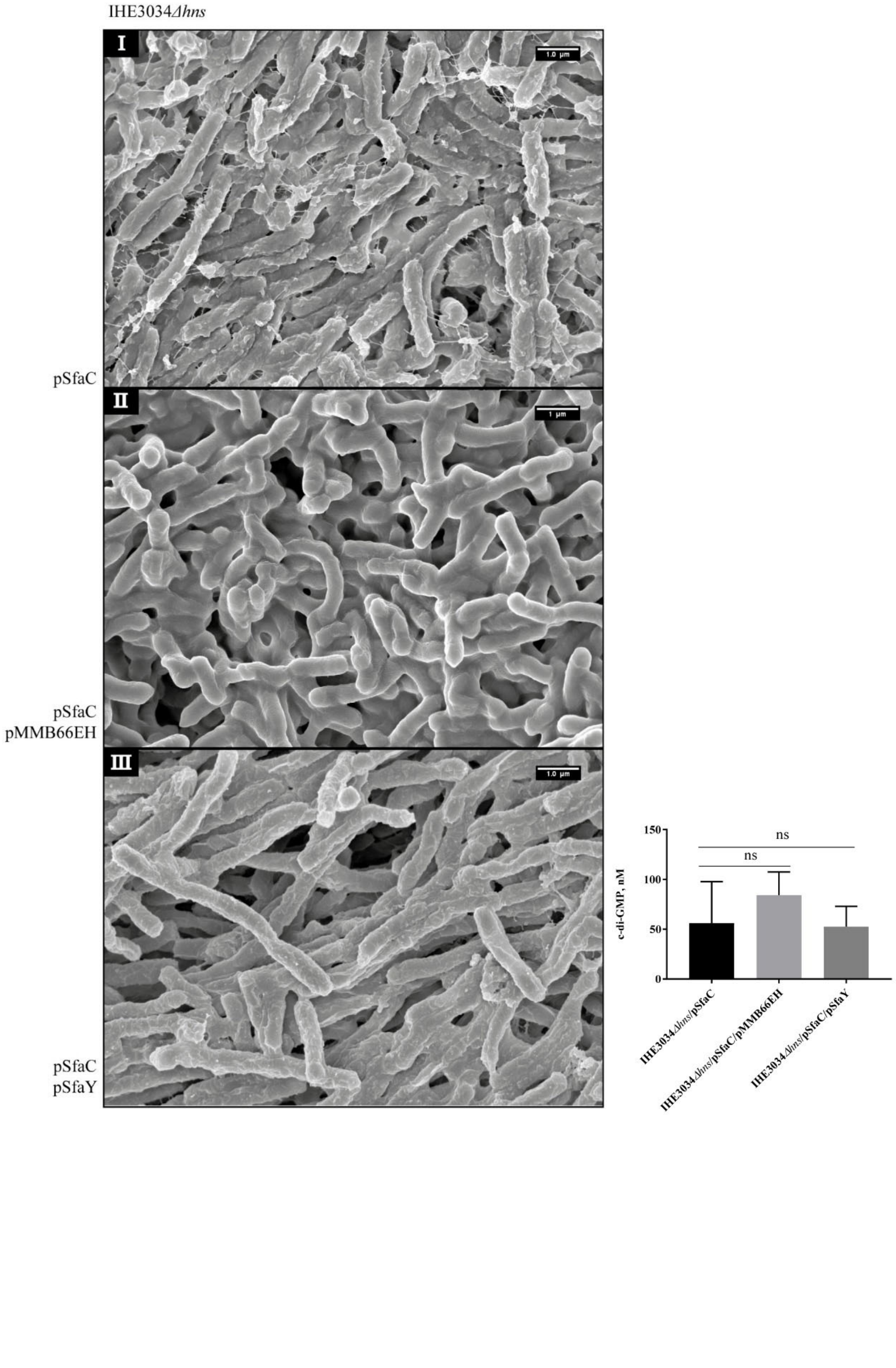

IHE3034Δhns
I
1.0 µm
pSfaC
II
1 µm
pSfaC
pMMB66EH
III
1.0 µm
pSfaC
pSfaY
c-di-GMP, nM
150
100
50
0
ns
ns
IHE3034Δhns/pSfaC
IHE3034Δhns/pSfaC/pMMB66EH
IHE3034Δhns/pSfaC/pSfaY

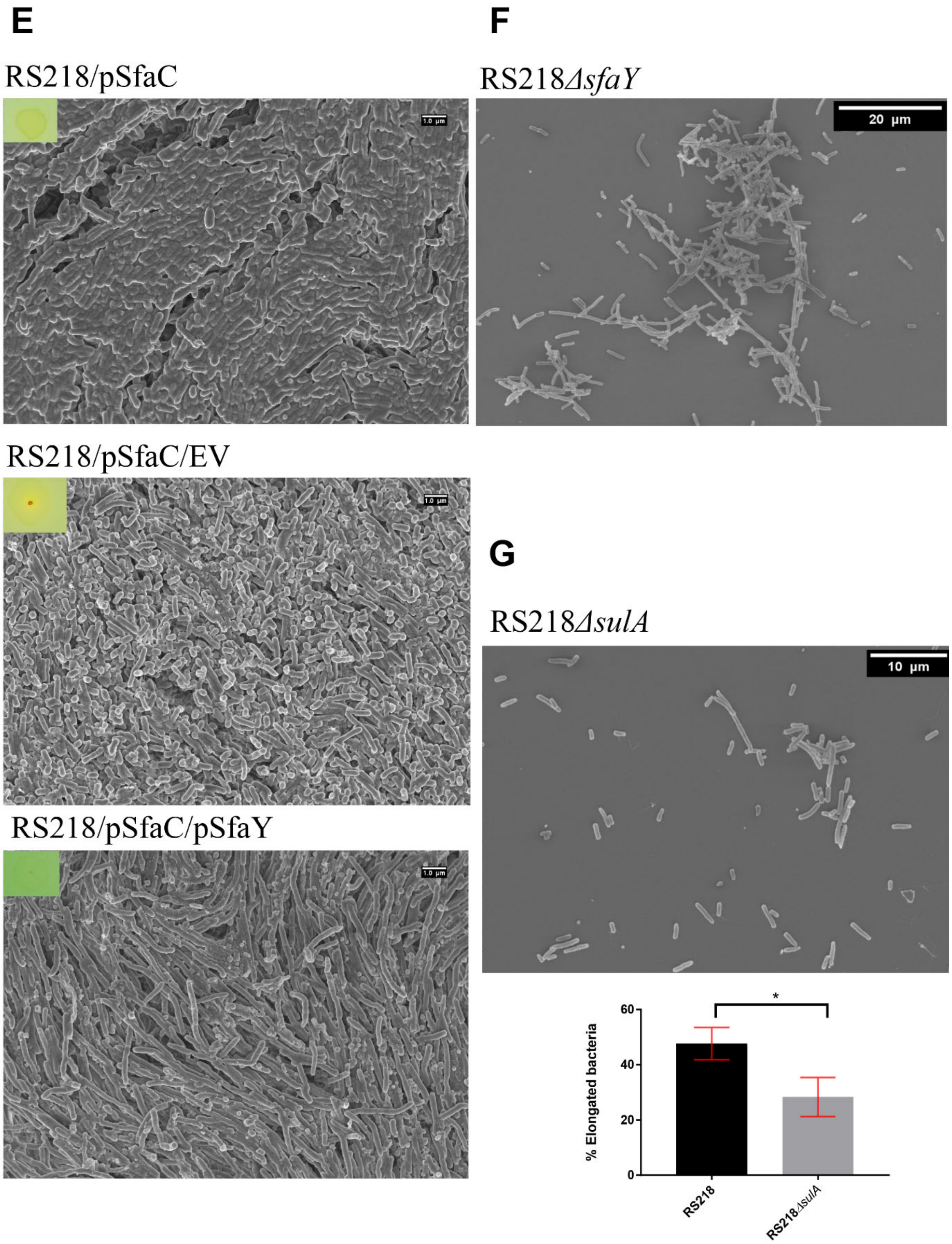
E
RS218/pSfaC
RS218/pSfaC/EV
RS218/pSfaC/pSfaY
F
RS218ΔsfaY
20 µm
G
RS218ΔsulA
10 µm
% Elongated bacteria
60
40
20
0
*
RS218
RS218ΔsulA

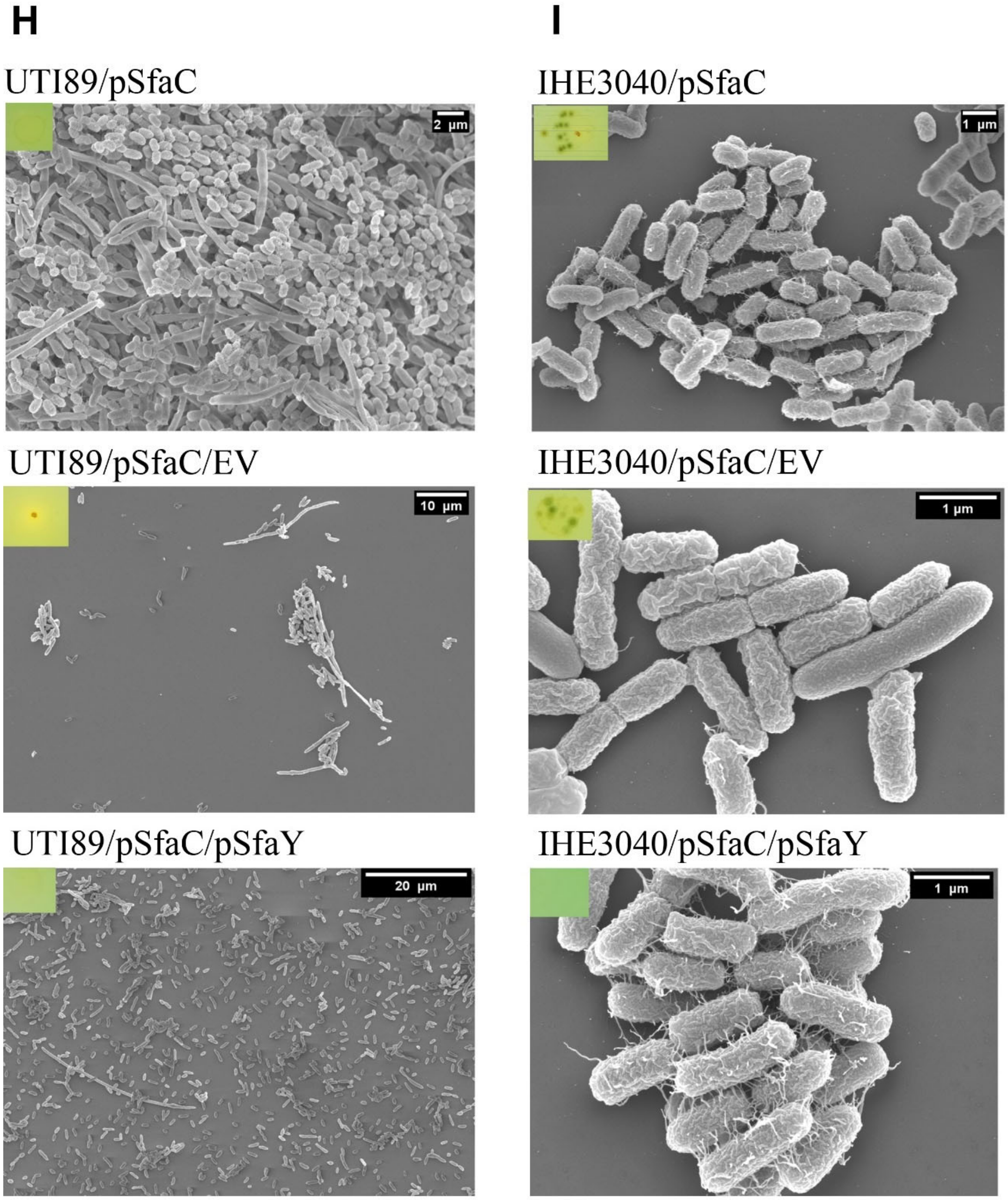
H
I
UTI89/pSfaC
2 µm
IHE3040/pSfaC
1 µm
UTI89/pSfaC/EV
10 µm
IHE3040/pSfaC/EV
1 µm
UTI89/pSfaC/pSfaY
20 µm
IHE3040/pSfaC/pSfaY
1 µm

**Figure S3. Quality assessment of the obtained metabolites from RS218 and IH3034, related to Fig. 5 and Fig. 6.**

**A.** Representative chromatograms of all the peaks obtained in the ESI (-) mode (IHE_1_N_mz_ML corresponds to a sample of IHE3034 and RS_1_N_mz_ML to a sample of RS218).

**B.** Sampling grouping of the IHE3034 (in red) and RS218 (in green) metabolites obtained from the ESI (-) mode based on partial least squares-discriminant (PLSD) and on orthogonal projections to latent structures discriminant (O-PLSD) analysis.

**C.** Discriminating metabolites from the ESI (-) mode based on the variable importance in projection (VIP) scores $\geq 1.5$.

**D.** Representative chromatograms of all the peaks obtained in the ESI (+) mode (IHE_1_N_mz_ML corresponds to a sample of IHE3034 and RS_1_N_mz_ML to a sample of RS218).

**E.** Sampling grouping of the IHE3034 (in red) and RS218 (in green) metabolites obtained from the ESI (+) mode based on partial least squares-discriminant (PLSD) and on orthogonal projections to latent structures discriminant (O-PLSD) analysis.

**F.** Discriminating metabolites from the ESI (+) mode based on the variable importance in projection (VIP) scores $\geq 1.5$.

**Fig. S3A ESI (-)**

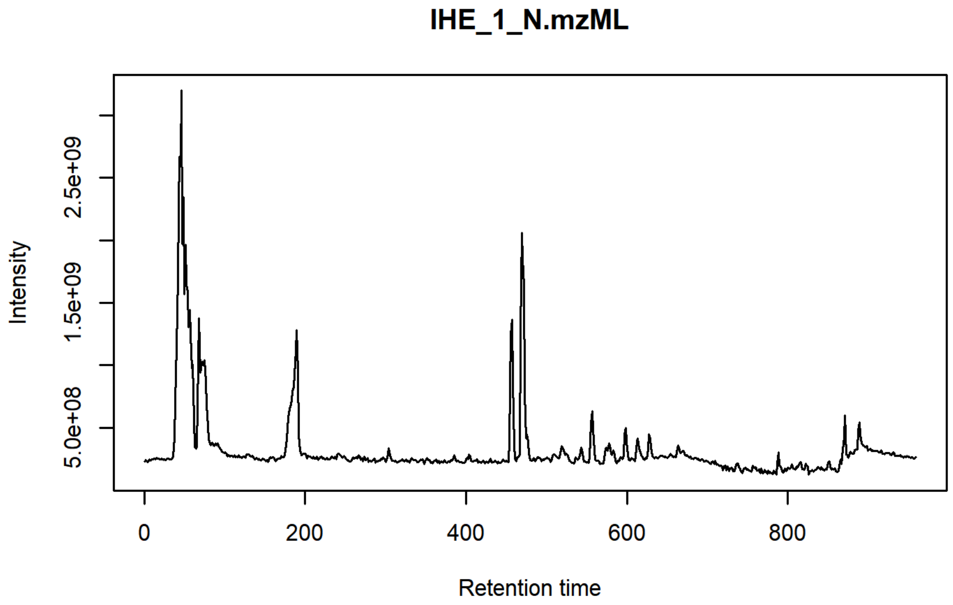
IHE_1_N.mzML
Intensity
5.0e+08
1.5e+09
2.5e+09
0
200
400
600
800
Retention time

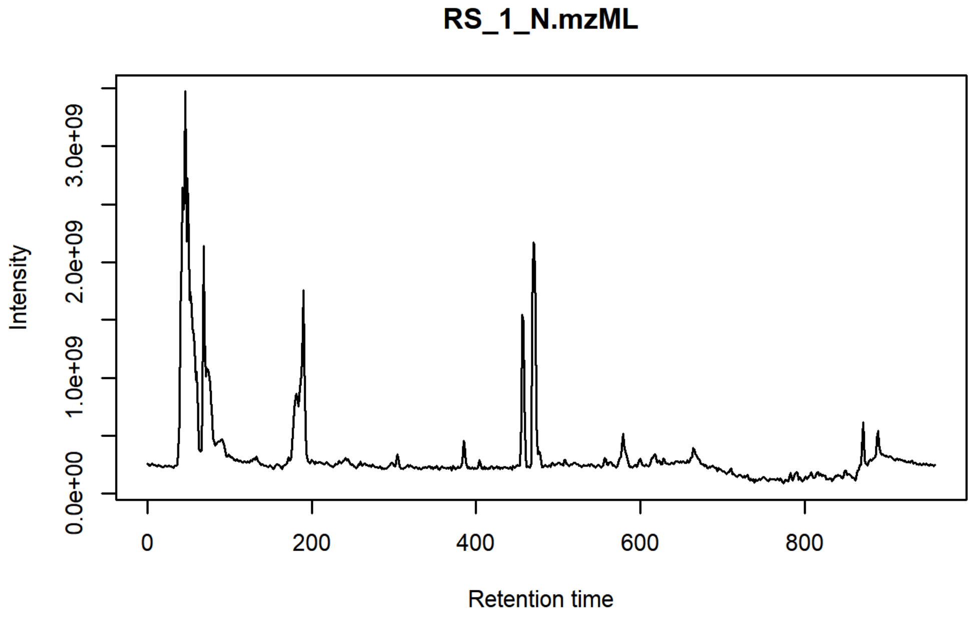
RS_1_N.mzML
Intensity
0.0e+00
1.0e+09
2.0e+09
3.0e+09
0
200
400
600
800
Retention time

**Fig. S3B. PLS-DA**

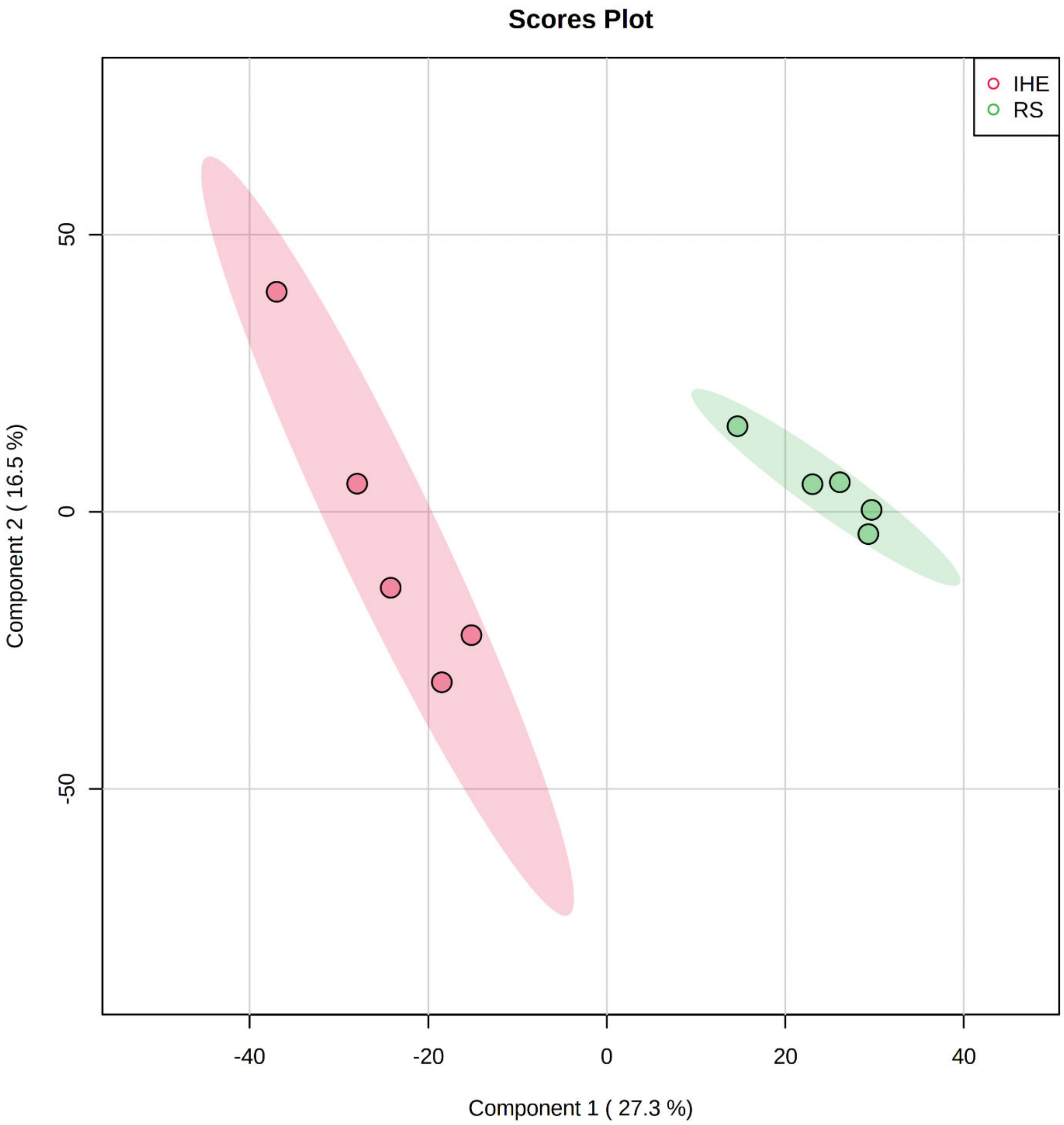

**Fig. S3B (continued) OPLS-DA**

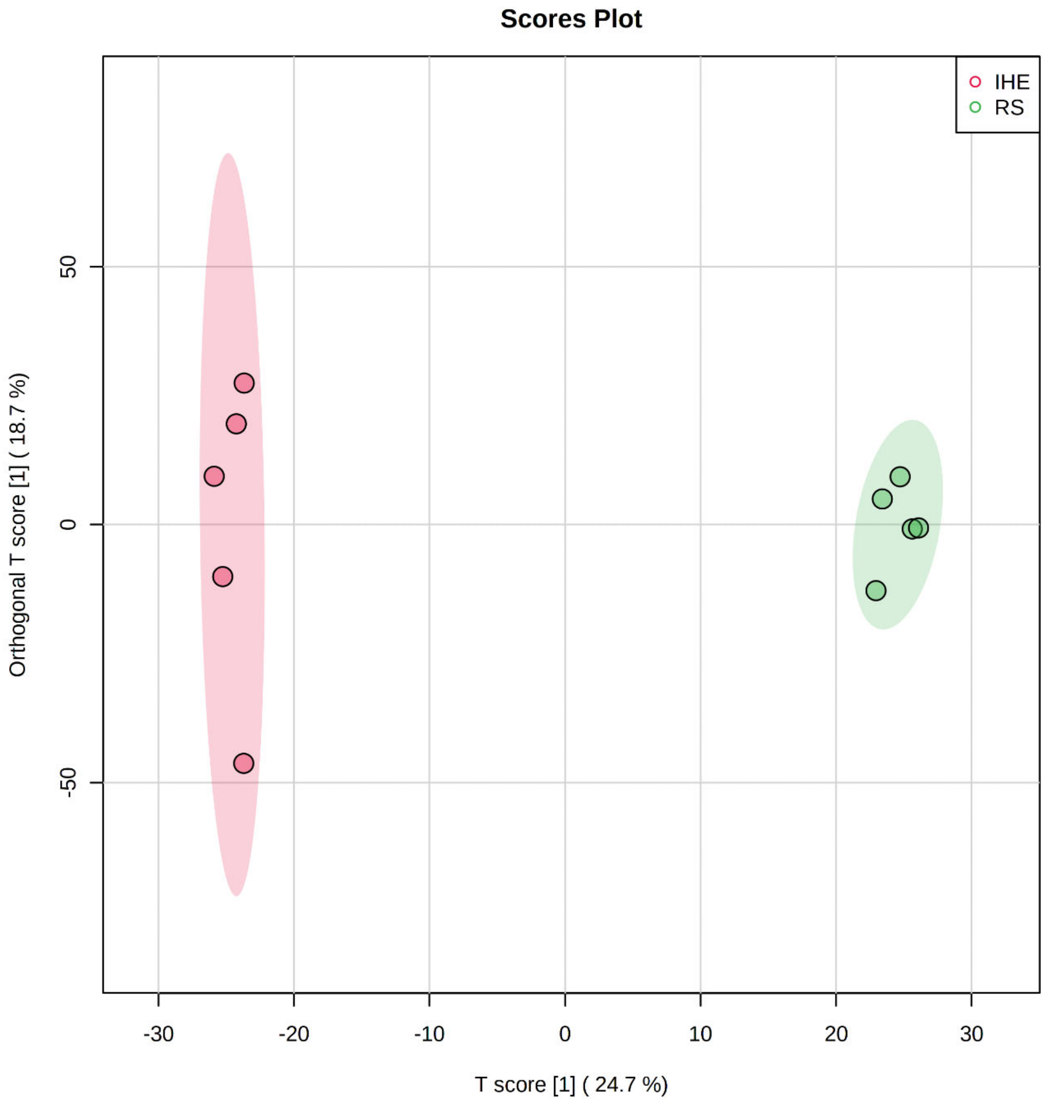

**Fig. S3C**

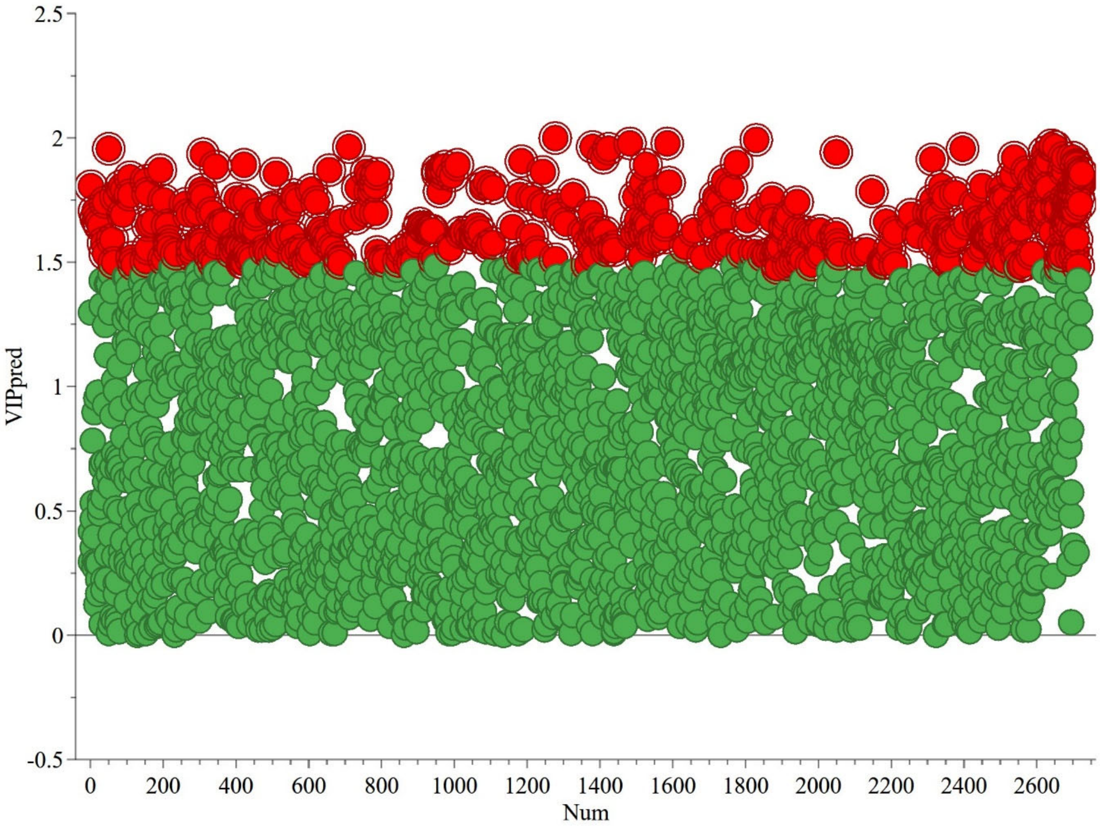


**Fig. S3D ESI (+)**

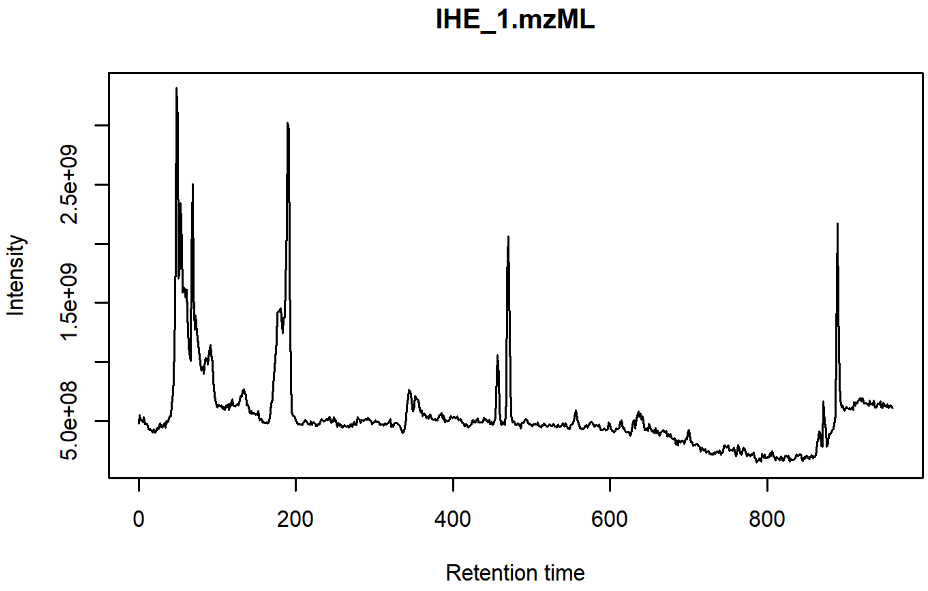

**Fig. S3D (continued)  ESI (+)**

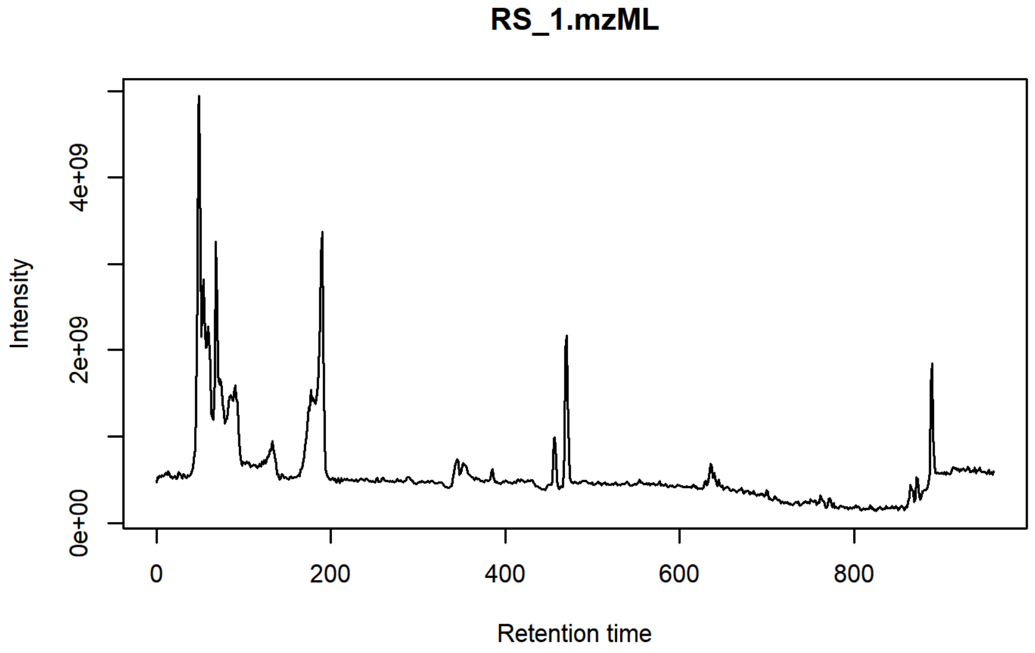

**Fig. S3E PLS-DA**

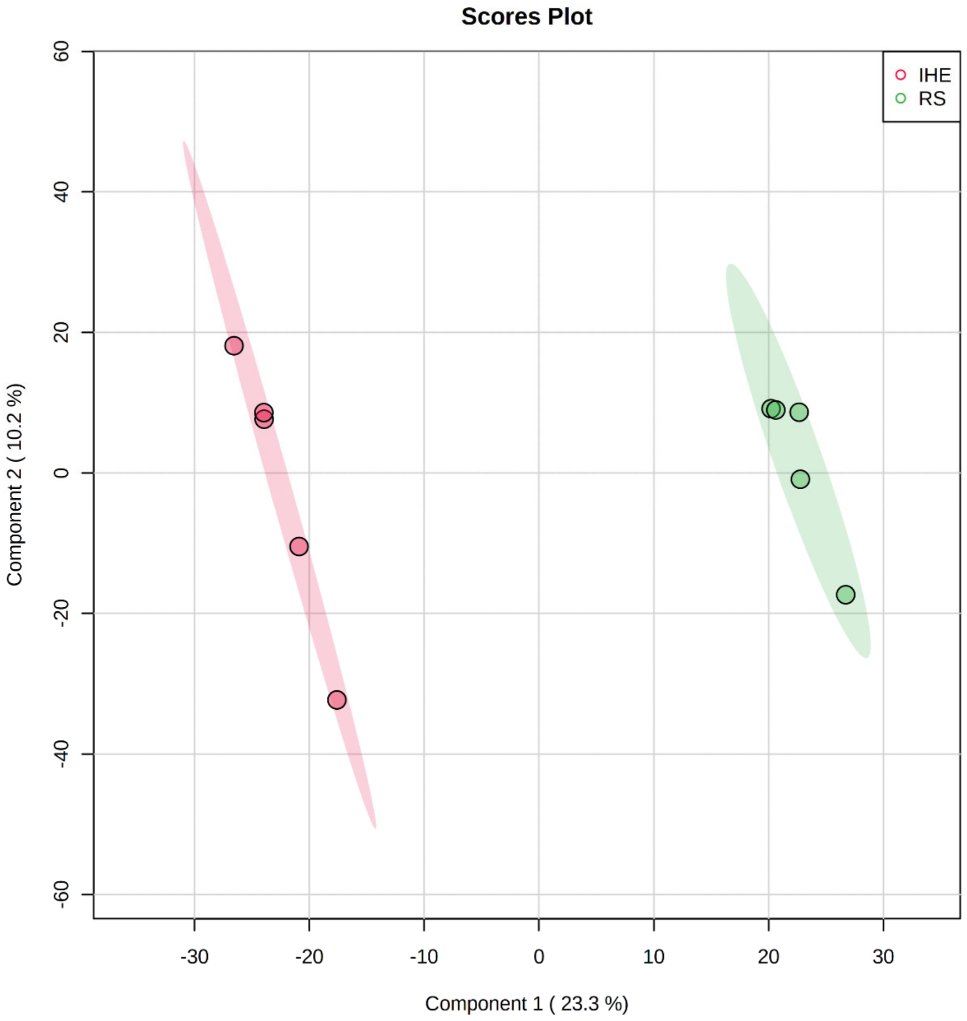

**Fig. S3E (continued)  OPLS-DA**

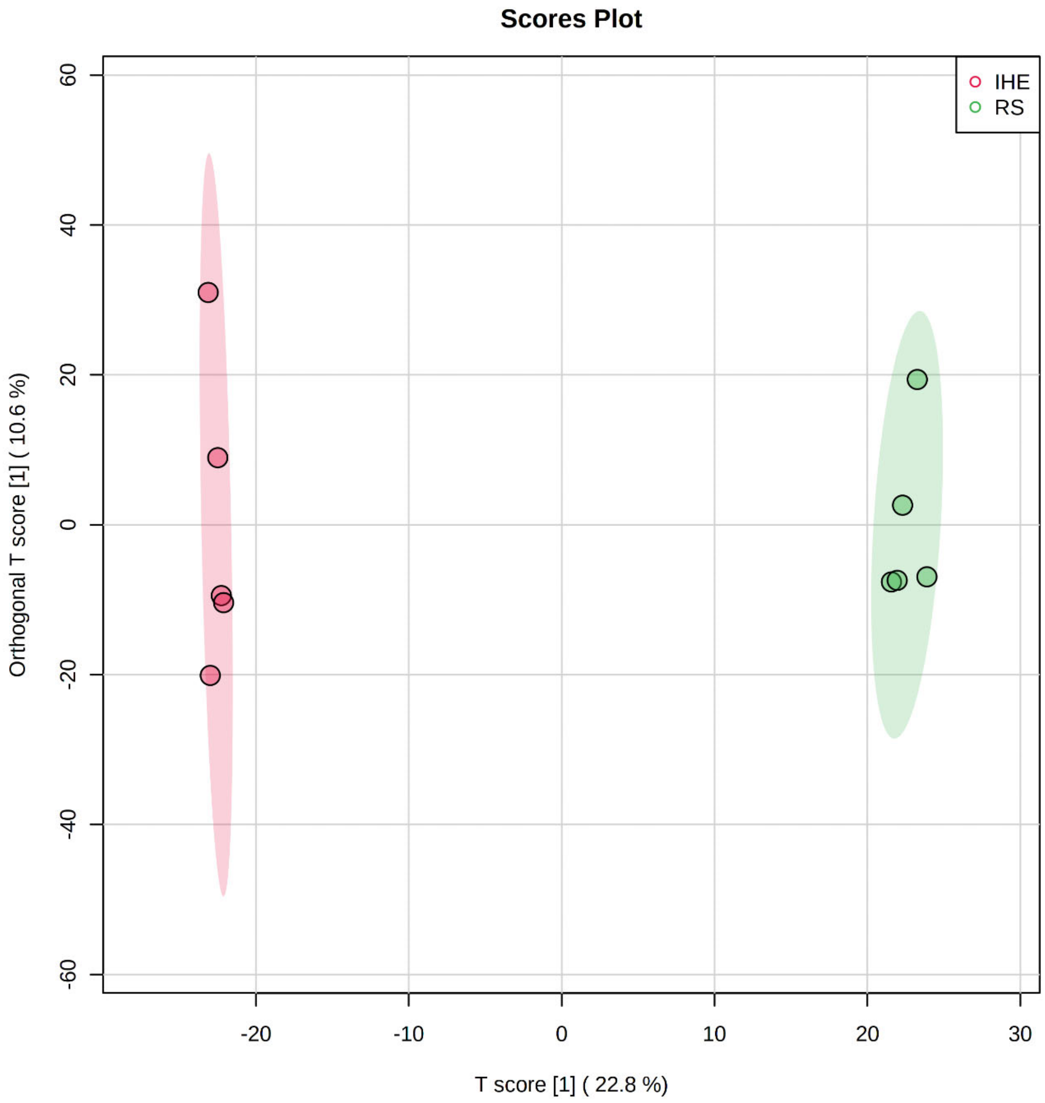

**Fig. S3F**

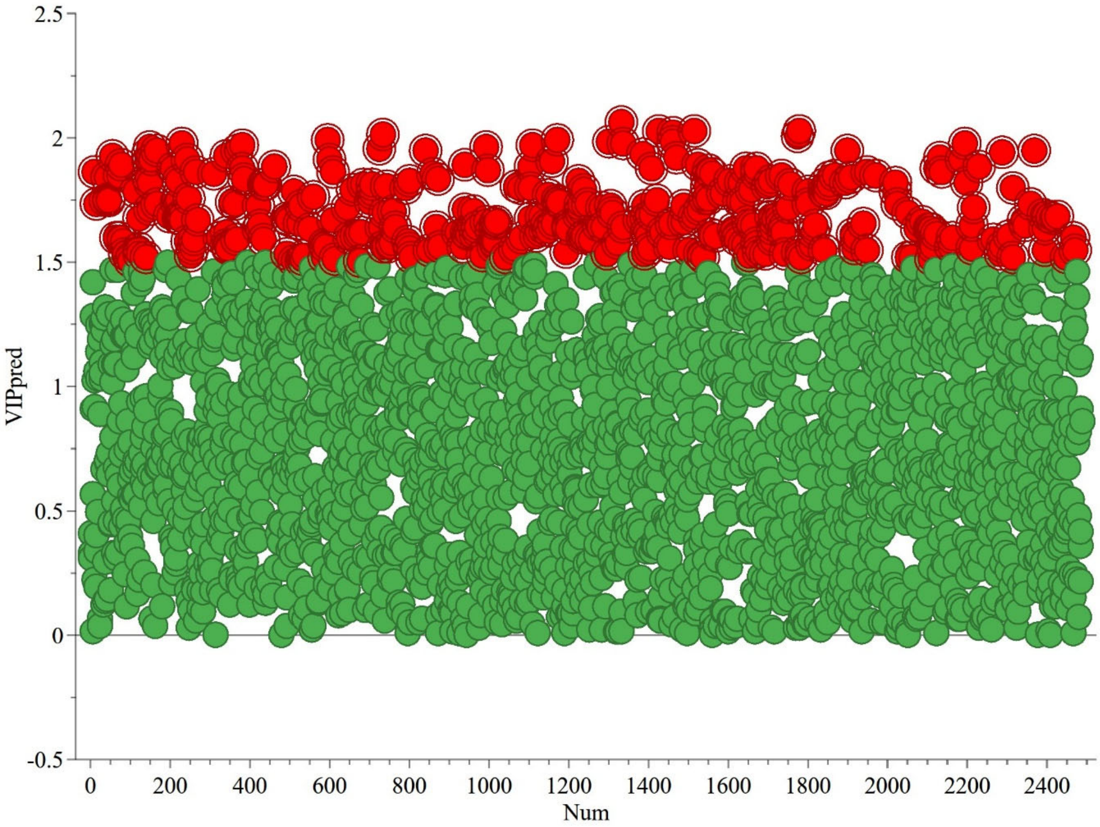
2.5
2
1.5
1
0.5
0
-0.5
VIPpred
0
200
400
600
800
1000
1200
1400
1600
1800
2000
2200
2400
Num

**Figure S4. KEGG correlation map (eco01100) of the perturbed metabolites in RS218, related to Fig. 5 and Fig. 6. The metabolic pathways identified in RS218 strain are represented as follows: nucleotide metabolism in red; cofactor metabolism in pink, amino acid metabolism in orange; energy metabolism in purple; carbohydrate metabolism in blue and lipid metabolism in green. The metabolites from the negative and positive data generated the same metabolic map.**

**Fig. S4 (continued)**

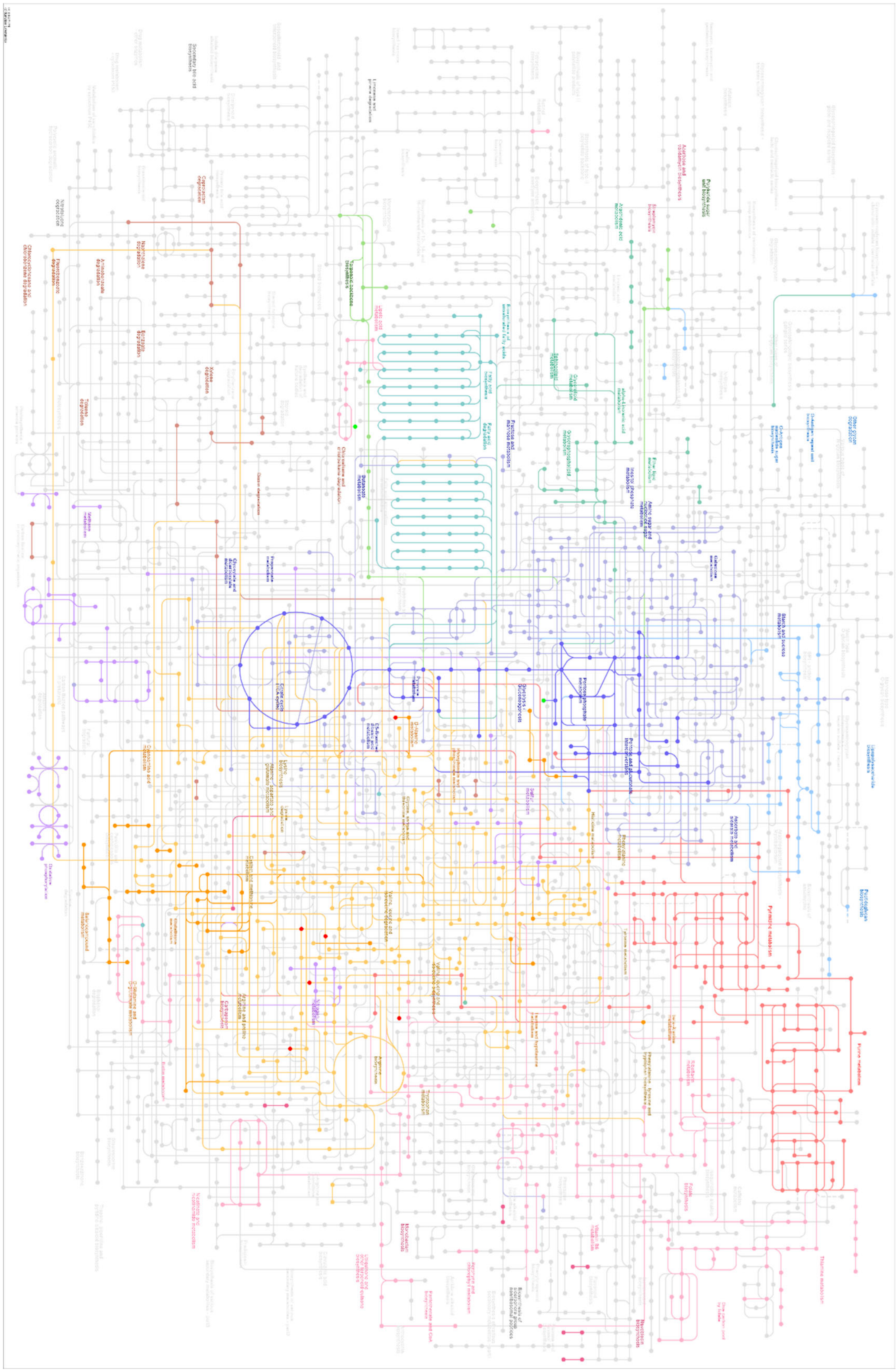

**Figure S5. Citrate uptake by *Escherichia coli*, related to Fig. 5 and Fig. 6.**

**A.** Citrate uptake by IHE3034 in the presence of 1% glucose (Koser Glc), 1% glucose and 15mM fumarate (Koser Glc Fum), 1% glucose and 15mM nitrate (Koser Glc Nit), and 1% Glc, 15mM fumarate and 15mM nitrate (Koser Glc Fum Nit).

**B.** Citrate utilization by EHEC strains incubated on Simmons' with 0.1% glucose embedded.

**C.** Citrate utilization by EAggEC strains incubated on Simmons' with 0.1% glucose embedded.

**D.** Citrate utilization by ETEC strains incubated on Simmons' with 0.1% glucose embedded.

**E.** Citrate utilization by EIEC strains incubated on Simmons' with 0.1% glucose embedded.

**F.** Citrate utilization by EPEC strains incubated on Simmons' with 0.1% glucose embedded.

**G.** Citrate utilization by AIEC strains incubated on Simmons' with 0.1% glucose embedded.

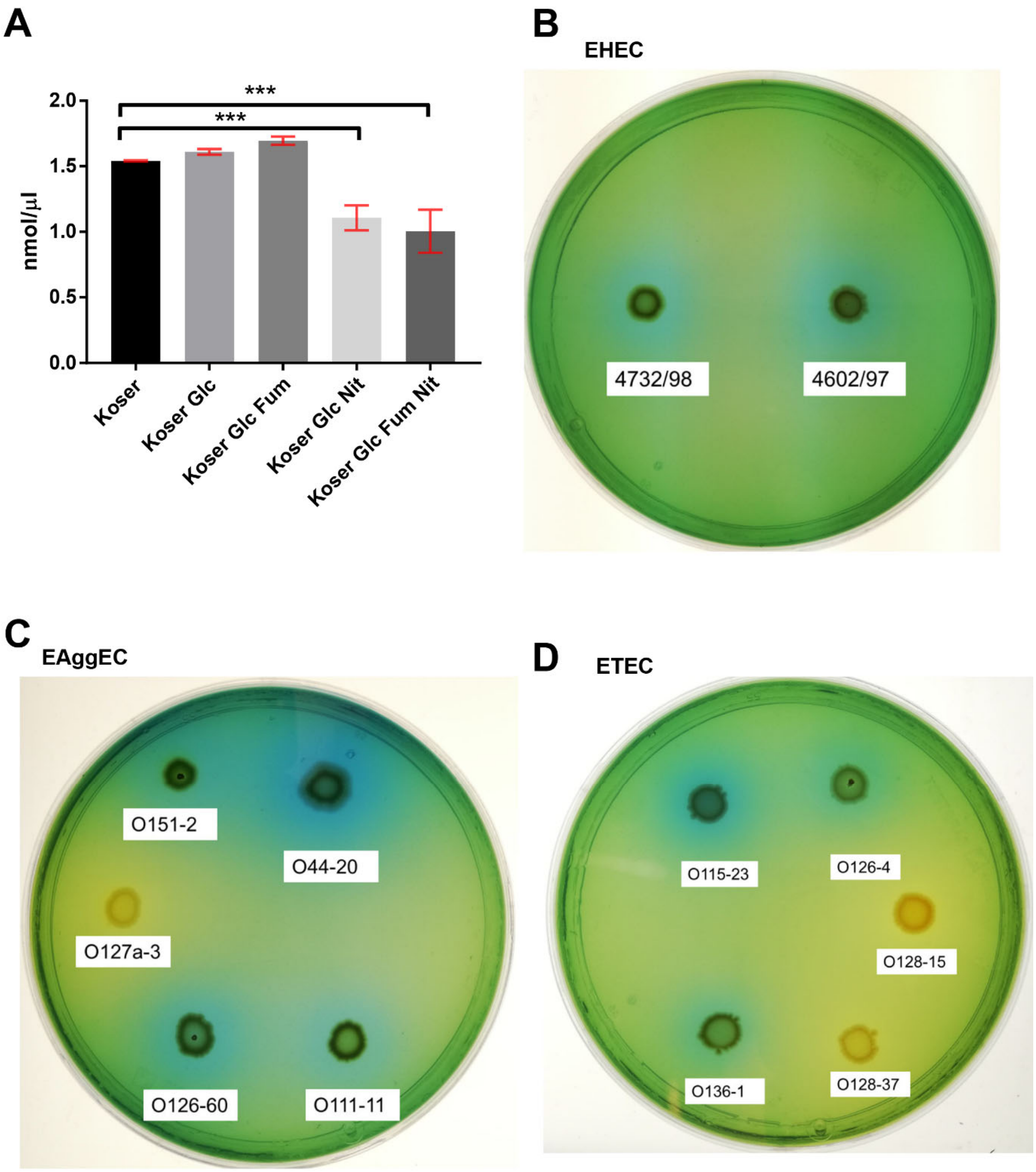
A
B
EHEC
***
***
2.0
1.5
1.0
0.5
0.0
nmol/µl
Koser
Koser Glc
Koser Glc Fum
Koser Glc Nit
Koser Glc Fum Nit
4732/98
4602/97
C
EAggEC
O151-2
O44-20
O127a-3
O126-60
O111-11
D
ETEC
O115-23
O126-4
O128-15
O136-1
O128-37

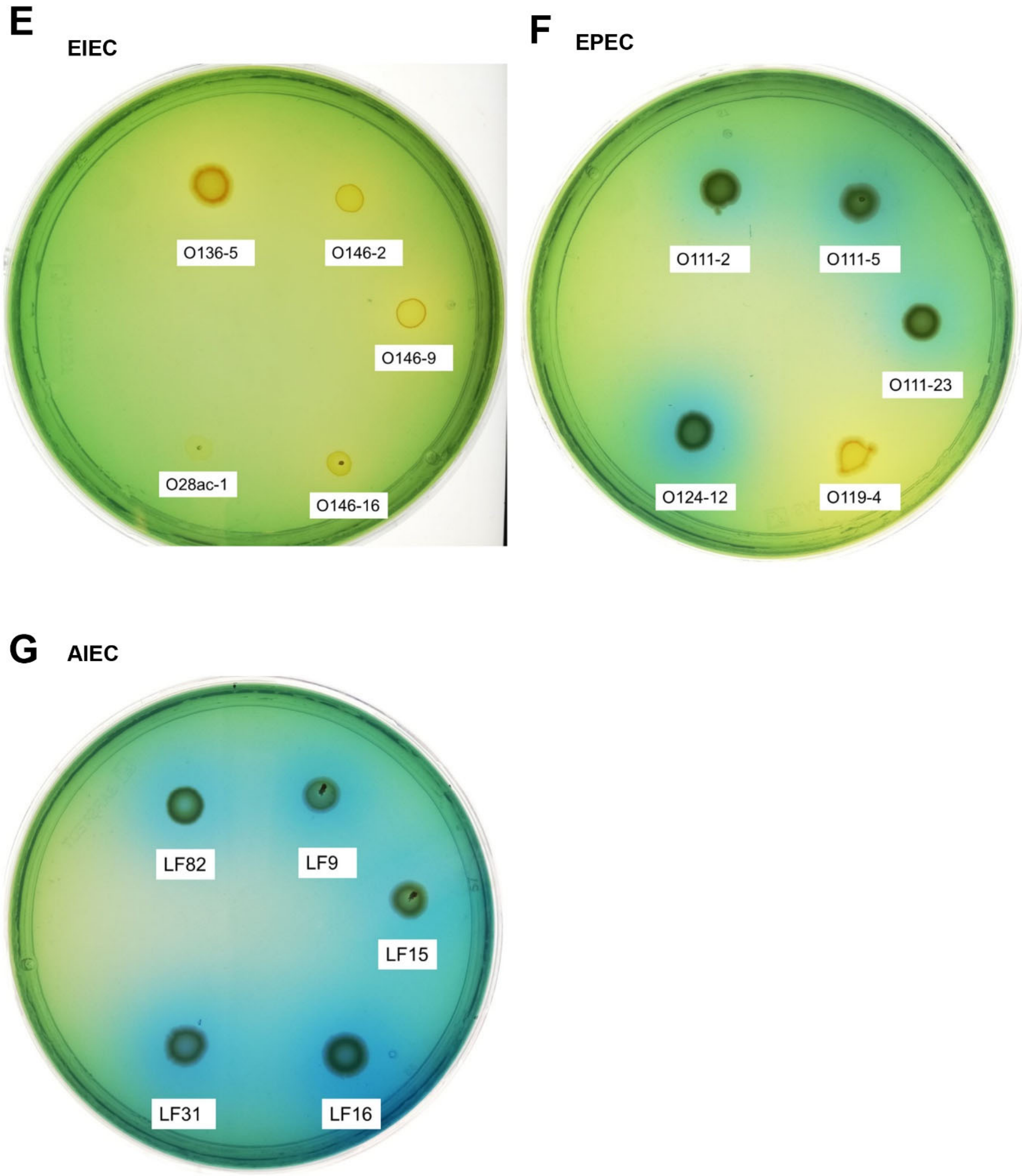
E
EIEC
O136-5
O146-2
O146-9
O28ac-1
O146-16
F
EPEC
O111-2
O111-5
O111-23
O124-12
O119-4
G
AIEC
LF82
LF9
LF15
LF31
LF16
